# Pyrrole‐Tethered Bisbenzoxazole Derivatives: Apoptosis‐Inducing Agents Targeting Breast Cancer Cells

**DOI:** 10.1111/cbdd.70078

**Published:** 2025-03-13

**Authors:** Burak Kuzu, Derya Yetkin, Ceylan Hepokur, Oztekin Algul

**Affiliations:** ^1^ Department of Pharmaceutical Chemistry, Faculty of Pharmacy Van Yuzuncu Yil University Van Türkiye; ^2^ Advance Technology Education Research and Application Centre Mersin University Mersin Türkiye; ^3^ Department of Biochemistry, Faculty of Pharmacy Sivas Cumhuriyet University Sivas Türkiye; ^4^ Department of Pharmaceutical Chemistry, Faculty of Pharmacy Mersin University Mersin Türkiye; ^5^ Department of Pharmaceutical Chemistry, Faculty of Pharmacy Erzincan Binali Yildirim University Erzincan Türkiye

**Keywords:** apoptosis, Bisbenzoxazole, cell cycle, cytotoxicity, MTT, pyrrole, synthesis

## Abstract

This study presents the design, synthesis, and biological evaluation of a series of novel pyrrole‐tethered bisbenzoxazole (PTB) derivatives as potential apoptosis‐inducing agents targeting the MCF‐7 human breast cancer cell line. The anticancer activity of these compounds was evaluated in vitro using the MTT assay, with tamoxifen serving as the reference therapeutic agent. Compounds **B8**, **B14**, and **B18** demonstrated remarkable cytotoxicity against MCF‐7 cells, exhibiting approximately 8‐fold lower IC_50_ values compared to tamoxifen, while showing minimal effects on healthy fibroblasts. Further investigations revealed that these compounds effectively induced early‐stage apoptosis and selectively arrested the cell cycle at the G1 phase in cancer cells. Gene expression analysis confirmed selective activation of the caspase‐9‐mediated apoptotic pathway in MCF‐7 cells, providing insights into their underlying molecular mechanisms. These findings highlight the promising potential of PTB derivatives as potent anticancer agents, laying the groundwork for the development of targeted therapies for breast cancer that leverage apoptosis induction for improved therapeutic outcomes.

## Graphical abstract

Design and synthesis of pyrrole‐tethered bisbenzoxazole (PTB) derivatives reveal potent anticancer activity against MCF‐7 breast cancer cells. Compounds **B8**, **B14**, and **B18** exhibit ~20‐fold lower IC_50_ values than tamoxifen, induce early‐stage apoptosis, and arrest the cell cycle at the G1 phase via caspase‐9 activation. These findings position PTB derivatives as promising candidates for targeted breast cancer therapy.

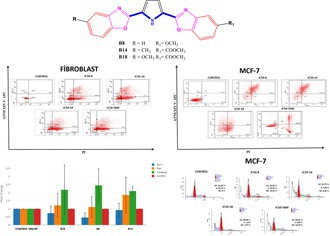

## 1 Introduction

New strategic approaches in drug design aim to selectively induce apoptosis in cancer cells while minimizing harm to normal cells, with targeted agents directed at proto‐oncogenes showing promising potential in advancing chemotherapy (Gerl and Vaux 2005; Ali et al. 2015). In this context, medicinal chemists have made exciting breakthroughs in designing cutting‐edge chemotherapy agents for targeted cancer treatment, harnessing the vast potential of nature's own compounds. Drug development often begins with compounds isolated from natural sources, and their structure–activity relationships (SAR) are studied.

**FIGURE 1:**
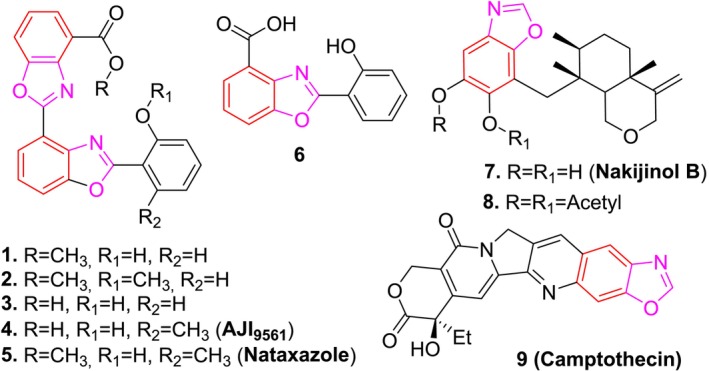
Benzoxazole derivatives with anticancer potential isolated from natural sources.

The benzoxazole ring structure offers considerable versatility, allowing for modifications that can enhance the biological activity and selectivity of these compounds (Zhang et al. 2018). These molecules can exert their anticancer effects by interacting with DNA, inhibiting enzymes, or inducing apoptosis (Abdullahi and Yeong 2024). Additionally, compounds derived from natural sources can serve as lead candidates, which are then further optimized through synthetic modifications to improve their potency, selectivity, and safety profile as therapeutic agents. For example, the bisbenzoxazole derivative UK‐1 (**1**) and its analogs (**2–3**) were obtained from *Actinomycete strains* and were determined to have antiproliferative properties in B16, HeLa, and P338 cancer cell lines (Ueki and Taniguchi 1997; Ueki et al. 1998). It has been discovered that the compounds AJI9561 (**4**) (Sato et al. 1998), nataxazole (**5**) (Sommer et al. 2008), and a similar benzoxazole derivative (**6**) (Kumar et al. 2002), which were also reported as UK‐1 analogs and isolated from s*treptomyces strains*, have anticancer and antibacterial properties. It was determined that Nakijinol B (**7**) and the diacetate derivative (**8**) obtained from *Dactylospongia elegans strains* had antiproliferative activity on SF‐268, H460, MCF‐7, and HT‐29 cancer cell lines (Ovenden et al. 2011). Finally, it has been reported that the compound camptothecin (**9**) obtained from *Camptotheca acuminata strains* has anticancer properties (Peel et al. 1995) (Figure 1). All of these pharmacologically active compounds derived from natural sources share the common feature of containing a benzoxazole ring in their structures.

**FIGURE 2:**
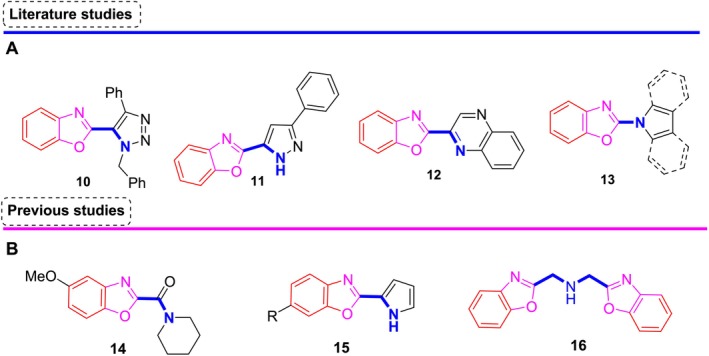
Benzoxazole derivatives with antiproliferative effects. (A) Various heterocyclic system‐tethered benzoxazole derivatives reported in the literature. (B) Benzoxazole derivatives developed in our previous studies with an antiproliferative activity profile.

Benzoxazole and isostere structures are biosteres of DNA nucleotides, and their ability to interact with DNA and exhibit high affinity for various biological receptors allows medicinal chemists to develop novel benzoxazole‐based synthetic heterocyclic compounds for targeted anticancer activities (Périgaud et al. 1992; Kuzu et al. 2024). In the literature, it is seen that benzoxazole rings, especially those developed with different synthetic strategies, have high antiproliferation effect potential in various cancer cell lines (Sattar et al. 2020). For example, benzoxazole‐triazole hybrid (**10**) (Srivastava et al. 2015) in HeLa and HepG2 cell lines (IC_50_: 5–30 μg/mL), benzoxazole‐pyrazole (**11**) (Belal and Abdelgawad 2017) in A549 and MCF‐7 cell lines (IC_50_: 0.12–0.19 μM), benzoxazole‐quinoxaline (**12**) (Desai et al. 2020) in MCF‐7 and MDA‐MB‐231 breast cancer cell lines (IC_50_: 0.53 μM and 0.50 μM, respectively), and benzoxazole‐pyrrole, ‐indole, or ‐carbazole hybrid compounds (**13**) (Hedidi et al. 2014) in A2058 melanoma cancer cells (GI: 84.3%) have been reported to exhibit high cytotoxic activity (Figure 2A).

Our research group focuses on the development of novel synthetic benzoxazole derivatives, inspired by the benzoxazole derivative UK‐1, a compound isolated from natural sources, as well as other relevant literature. In our previous studies, benzoxazole‐2‐carboxamide derivatives demonstrated significant antiproliferative activity in breast cancer cell lines. Notably, the most active compound (**14**) induced a G0/G1 phase arrest (71.2%) in MCF‐7 cells, as determined through cell cycle analysis (Kuzu, Hepokur, Alagoz, et al. 2022; Kuzu, Hepokur, Turkmenoglu, et al. 2022).

Building on these findings, we designed a new series of 2‐aryl benzoxazole derivatives based on the structure of compound (**14**) and evaluated their efficacy against A549, MCF‐7, C6, and HT‐29 cell lines using the XTT assay. The results revealed that these derivatives exhibited higher selectivity for the MCF‐7 breast cancer cell line. Among the 25 molecules tested, the pyrrol‐2‐yl derivative (**15**) emerged as the most potent, with an IC_50_ value of 4.29 μM in the MCF‐7 cell line, surpassing the activity of the reference compound 5‐fluorouracil (5‐FU; IC_50_: 5.06 μM) (Kuzu, Hepokur, Alagoz, et al. 2022; Kuzu, Hepokur, Turkmenoglu, et al. 2022). These findings highlight the promising potential of 2‐heteroaryl benzoxazole derivatives as selective agents for breast cancer treatment.

Furthermore, we explored bisbenzoxazole derivatives featuring heteroatom‐containing linker chains, designed based on the bisbenzoxazole structure of UK‐1. Among these, the compound (**16**) with a ‐CH_2_‐NH‐CH_2_‐ linker chain exhibited exceptional antiproliferative activity across multiple cancer cell lines, including A549, A498, HeLa, and HepG2, with IC_50_ values ranging from 0.56 to 2.24 μM (Ersan et al. 2020). Remarkably, compound (**16**) demonstrated a fourfold higher selectivity for the A549 lung cancer cell line compared to other tested cancer cell lines, while displaying minimal cytotoxicity against the Vero healthy cell line (Figure 2B).

Based on the findings from these studies, the structure–activity relationship (the SAR) of the synthesized compounds with respect to their antiproliferative activity can be summarized as follows:

- Conjugated system featuring a benzoxazole moiety at the second position: The placement of the benzoxazole ring at this position is pivotal in enhancing the compounds' antiproliferative potency.
- Incorporation of a carbon‐linked amine group within the benzoxazole ring: The presence of a carbon‐linked amine chain is critical for optimizing interactions with cellular targets, thereby improving the compounds' overall biological efficacy.
- Substituted bisbenzoxazole derivatives: The introduction of substitutions in bisbenzoxazole derivatives indicates that strategic structural modifications at specific positions can significantly enhance antiproliferative activity.

**FIGURE 3:**
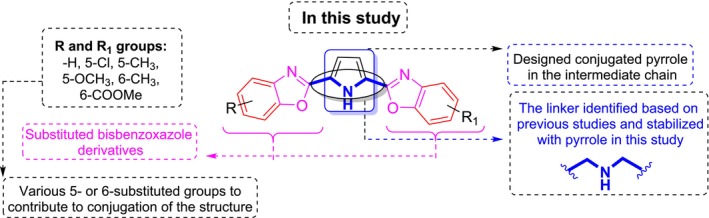
The pyrrole‐tethered bisbenzoxazole (PTB) derivatives designed in this study.

In this study, bisbenzoxazole derivatives were synthesized by linking substituted benzoxazoles through a pyrrole‐tethered structure, which stabilizes the linker ‐CH₂‐NH‐CH₂‐ chain. The conjugated and heterocyclic framework was strategically designed to optimize antiproliferative activity (Figure 3). The antiproliferative effects of the synthesized compounds were initially evaluated in MCF‐7 breast cancer cells. Compounds demonstrating the most potent antiproliferative activity were further analyzed for their effects on apoptosis and cell cycle progression using flow cytometry. Moreover, gene expression analysis related to cancer pathways was performed to elucidate the molecular mechanisms underlying their activity. These results provide valuable insights into the anticancer mechanisms of the newly developed bisbenzoxazole derivatives and hold promise for guiding the development of more effective targeted cancer therapies in drug discovery.

## 2 Results and Discussion

### 2.1 Chemistry

**FIGURE 4:**
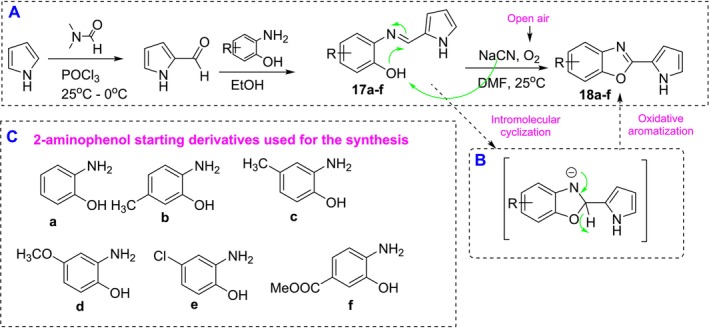
Synthesis of C‐2 pyrrole‐substituted benzoxazole derivatives. (A) General synthesis scheme. (B) Mechanistic intermediate in the formation of the benzoxazole ring. (C) Reagents used as starting materials in the synthesis of the target compounds.

Within the scope of this study, it was planned to synthesize C‐2 carbaldehyde pyrrole and then carry out the condensation reaction with 2‐aminophenol derivatives for synthesis studies. As the starting compound, pyrrole‐2‐carbaldehyde was synthesized via the Vilsmeier–Haack method in our previous studies (Kuzu, Hepokur, Alagoz, et al. 2022; Kuzu, Hepokur, Turkmenoglu, et al. 2022; Kuzu and Kuzu 2023). Schiff bases (**17a–f**) formed as a result of the condensation reaction of pyrrole‐2‐carbaldehyde with 2‐aminophenol derivatives were then reacted with NaCN to furnish benzoxazol‐2‐(pyrrol‐2‐yl) (**18a–f**) derivatives (Figure 4A). Intramolecular cyclization, which occurs as a result of the attack of phenolic OH on the Schiff base carbonyl with the help of NaCN catalyst, and the transformation of the benzoxazoline structure into the benzoxazole structure by aromatization through the oxidation reaction with the air oxygen in the environment (Figure 4B), is known in the literature (Cho et al. 2012). The 2‐aminophenol derivatives used in the planned synthesis design are presented in Figure 4C.

**FIGURE 5:**
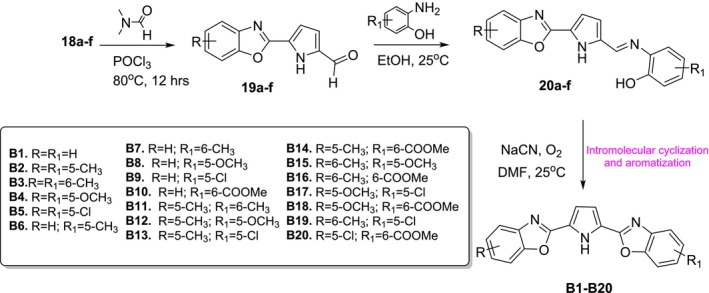
Synthesis of pyrrole‐tethered bisbenzoxazoles.

Compounds (**18a–f**) obtained from the above reaction were used as starting reagents in the second stage of the synthesis of bisbenzoxazoles. First, it was planned to make an aldehyde substitution at the 5th position of the pyrrole structure in compounds (**18a–f**). Preliminary experiments have shown that the Vilsmeier–Haack method used to obtain pyrrole‐2‐carbaldehyde does not react at room temperature at this step. It has been determined that pyrrole C‐5 carbonylation occurs when the reaction temperature is at reflux degrees. The reaction, which was optimized through preliminary trials, was completed by heating under reflux for 12 h, then neutralized with NaOH and extracted with 3 × 20 mL ethyl acetate and 50 mL water, and compounds (**19a–f**) were obtained. Then, similarly, Schiff bases (**20a–f**) were synthesized by first condensing (**19a–f**) with 2‐amino phenol derivatives and then reacting them with NaCN to furnish target PTB derivatives **B1–B20** (Figure 5).

The ^1^H‐ and ^13^C‐NMR spectra of the synthesized compounds **B1–B5**, which possess a symmetrical structure, exhibited overlapping peaks as expected. In contrast, the NMR spectra of the asymmetrical compounds **B6–B20** displayed distinct resonances. The ^1^H‐NMR spectra confirmed that all aromatic and aliphatic protons appeared at their anticipated chemical shifts, while the ^13^C‐NMR spectra showed signals consistent with the expected types and numbers of carbons for each compound. Additionally, elemental analysis for C, H, and N elements was performed. The results showed a maximum deviation of ±0.4 from the theoretically calculated values, supporting the accuracy of the synthesized compounds' structures.

### 2.2 Biological Evaluation

#### 2.2.1 Cytotoxicity Assessment

The cytotoxic effects of PTB derivatives on MCF‐7 breast cancer cells were evaluated using the MTT assay at various concentrations (100 nM, 1 μM, 10 μM, 50 μM, and 100 μM). The results, summarized in Table 1, revealed that compounds **B8**, **B14**, and **B18** significantly inhibited cell proliferation, with only 50% cell viability observed at higher concentrations. These compounds were selected for further investigation based on their potent activity.

**TABLE 1:** The IC_50_ values of PTB derivatives after 24, 48, and 72 h of MTT assay for MCF‐7 and HDFa cell lines.

Compounds	IC_50_ (μM) for MCF‐7 cell lines			IC_50_ (μM) for HDFa cell lines^a^		
	24 h	48 h	72 h	24 h	48 h	72 h
**B1**	3.89 ± 1.15	2.96 ± 1.02	3.13 ± 1.12	89.42 ± 1.23	89.93 ± 0.43	100 ≤
**B2**	4.60 ± 2.20	4.50 ± 0.23	2.06 ± 1.67	96.29 ± 1.03	94.34 ± 1.56	99.31 ± 1.03
**B3**	3.30 ± 1.96	4.38 ± 0.98	3.53 ± 0.65	100 ≤	100 ≤	100 ≤
**B4**	4.56 ± 1.56	4.19 ± 1.86	3.11 ± 0.56	97.32 ± 1.34	99.23 ± 0.32	100 ≤
**B5**	3.73 ± 1.05	2.69 ± 2.01	3.28 ± 1.32	95.32 ± 1.92	97.46 ± 2.93	100 ≤
**B6**	2.81 ± 0.76	3.09 ± 1.23	3.02 ± 0.89	98.53 ± 1.02	98.99 ± 1.76	99.08 ± 1.73
**B7**	4.08 ± 1.23	4.68 ± 0.98	2.53 ± 1.82	97.32 ± 1.06	98.48 ± 2.00	99.99 ± 1.03
**B8**	4.40 ± 0.79	1.89 ± 0.12	3.09 ± 0.21	100 ≤	100 ≤	100 ≤
**B9**	3.84 ± 1.12	3.23 ± 0.63	2.77 ± 1.23	95.39 ± 1.29	97.48 ± 1.99	101.38 ± 1.38
**B10**	3.66 ± 1.34	2.88 ± 0.45	2.44 ± 2.11	97.21 ± 1.76	99.32 ± 1.34	99.93 ± 1.65
**B11**	4.55 ± 0.09	3.49 ± 0.68	2.44 ± 1.19	95.32 ± 1.65	97.42 ± 1.47	99.43 ± 1.23
**B12**	3.88 ± 0.23	3.49 ± 0.46	3.11 ± 0.87	100 ≤	100 ≤	100 ≤
**B13**	3.43 ± 1.56	3.60 ± 0.56	3.28 ± 1.36	100 ≤	100 ≤	100 ≤
**B14**	3.28 ± 0.74	0.95 ± 1.01	3.02 ± 2.18	94.53 ± 1.37	96.33 ± 1.38	97.42 ± 2.83
**B15**	2.49 ± 1.64	8.09 ± 2.06	2.53 ± 1.56	100 ≤	100 ≤	100 ≤
**B16**	3.03 ± 1.35	2.82 ± 2.14	2.93 ± 1.02	95.33 ± 1.03	97.32 ± 1.09	99.32 ± 1.07
**B17**	3.70 ± 1.12	3.44 ± 1.12	3.03 ± 0.76	98.34 ± 1.57	98.99 ± 1.96	99.02 ± 1.00
**B18**	3.53 ± 1.35	1.30 ± 0.98	2.69 ± 0.73	98.34 ± 1.58	100 ≤	100 ≤
**B19**	2.83 ± 0.87	2.97 ± 1.12	3.42 ± 1.12	97.32 ± 1.34	97.33 ± 1.20	99.82 ± 0.89
**B20**	2.97 ± 1.13	3.25 ± 0.97	3.07 ± 2.96	95.35 ± 1.94	97.32 ± 1.43	100 ≤
**Tamoxifen**	8.93 ± 1.97	8.03 ± 1.58	7.59 ± 0.87	50.64 ± 1.74	49.72 ± 0.67	47.82 ± 0.63

^a^ In the values given in the table, IC_50_ values greater than 100 were not calculated. IC_50_ values obtained in healthy cells are shown by xCELLigence analysis and are given in the Data S1.

The MTT assay demonstrated that the PTB derivatives were effective at significantly lower concentrations than tamoxifen. After 48 h of exposure, the IC_50_ values for compounds **B8**, **B14**, and **B18** were determined to be 1.89 μM, 0.95 μM, and 1.30 μM, respectively, compared to tamoxifen's IC_50_ value of 8.03 μM (Table 1). This indicates that the derivatives exhibited cytotoxicity nearly eight times more potent than tamoxifen.

The cytotoxicity of these derivatives was also assessed in dermal fibroblast cells using xCELLigence analysis as detailed in the Supporting Information. The results indicated that the IC_50_ values of the derivatives did not adversely affect healthy fibroblast cells, highlighting their selective toxicity toward cancer cells. These findings suggest that the active compounds possess potent anticancer properties with reduced toxicity to normal cells compared to tamoxifen, a standard breast cancer treatment.

All tested compounds demonstrated significantly lower IC_50_ values compared to tamoxifen, confirming their superior antiproliferative activity against MCF‐7 cells. The structure–activity relationship (SAR) analysis based on 48‐h MTT results revealed the following insights:

##### 2.2.1.1 Symmetric vs. Asymmetric Derivatives

Symmetric bisbenzoxazole derivatives (**B1**–**B5**) exhibited lower antiproliferative activity than their asymmetric counterparts, emphasizing the importance of substituents on the benzoxazole ring. Among the symmetric derivatives, **B5**, containing the 5‐Cl substituent, showed the highest cytotoxicity, followed by the unsubstituted bis derivative **B1**. Other symmetric bisbenzoxazole derivatives (**B2**, **B3**, and **B4**) similarly exhibited low cytotoxic potential, highlighting the necessity of specific functional groups for activity enhancement.

##### 2.2.1.2 Mono‐Substituents

Among the derivatives where one benzoxazole ring is unsubstituted and the other is mono‐substituted (**B6**, **B7**, **B8**, **B9**, and **B10**), the compound **B8**, containing the 5‐OCH_3_ substituent (IC_50_: 1.89 μM), was the most effective, followed by **B10**, containing the 6‐COOMe group (IC_50_: 2.88 μM), which showed moderate activity. Within this group, **B7** (containing the 6‐CH_3_ substituent) exhibited the lowest cytotoxic effect (IC_50_: 4.68 μM), while **B6** and **B9** (containing 5‐CH_3_ and 5‐Cl, respectively) displayed moderate effects. These results underscore the importance of the position and nature of substituents in maximizing activity.

##### 2.2.1.3 Di‐Substituents

Among the derivatives containing different di‐substituents (**B15**, **B13**, **B11**, **B12**, **B17**, **B20**, **B19**, **B16**, **B18**, and **B14**), a significant increase in cytotoxicity was observed in **B14**, **B18**, and **B16**, which were formed by combining the 6‐COOMe group with 5‐CH_3_, 5‐OCH_3_, and 6‐CH_3_ substituents. These findings demonstrate that the presence of di‐substituents, particularly in combination with electron‐withdrawing groups, substantially enhances the overall cytotoxic activity and supports the potential of di‐substituted bisbenzoxazole derivatives as potent anticancer agents.

##### 2.2.1.4 Halogen Substituents (5‐Cl)

Compounds containing the 5‐Cl substituent (e.g., **B5**, **B9**, **B13**, **B17**, **B19**, and **B20**) exhibited considerable potency. The electron‐withdrawing properties of halogens likely enhance activity by modulating the electronic distribution within the molecule, thereby facilitating better interactions with target proteins and overall biological activity.

##### 2.2.1.5 Methyl Substituents (5‐CH_3_ and 6‐CH_3_ )

Compounds with the 5‐CH_3_ group generally exhibited moderate activity. For example, **B2** and **B6** showed lower IC_50_ values, while **B14** (containing the synergistic 6‐COOMe group) exhibited the highest activity. A comparison between **B6** (containing 5‐CH_3_) and **B7** (containing 6‐CH_3_) clearly demonstrated that the 5‐CH_3_ group increases cytotoxic activity. Additionally, when combined with 5‐OCH_3_ or 6‐COOMe groups, as seen in **B12**, **B15**, **B14**, and **B16**, the cytotoxic effect was notably enhanced in the 5‐CH_3_ combinations.

##### 2.2.1.6 Carboxylic Ester Substituent (6‐COOMe)

The 6‐COOMe group emerged as the most effective substituent for enhancing activity. Compound **B14** (IC_50_: 0.95 μM) exhibited the highest potency among all derivatives, likely due to the polar and electron‐withdrawing nature of the ester group, which facilitates strong hydrogen bonding and electrostatic interactions with target proteins, thereby increasing efficacy.

##### 2.2.1.7 Methoxy Substituents (5‐OCH_3_ )

Compounds with a 5‐OCH_3_ group displayed moderate activity, although **B18** (IC_50_: 1.30 μM) demonstrated higher potency than other derivatives in this category. This suggests that while the 5‐OCH_3_ group is moderately active on its own, its activity can be significantly enhanced when combined with other substituents.

In conclusion, the SAR analysis highlights the crucial role of the 6‐COOMe group in enhancing antiproliferative activity, primarily due to its polar and electron‐withdrawing characteristics, which facilitate strong interactions with target proteins. The 5‐CH_3_ and 5‐OCH_3_ groups further enhance activity when combined with 6‐COOMe, while the 5‐Cl and 6‐CH_3_ groups provide moderate contributions. Based on these findings, compounds **B8**, **B14**, and **B18** have been identified as the most promising candidates for further studies due to their potent anticancer properties and selective cytotoxicity toward breast cancer cells. These results underscore the importance of considering the type and position of substituents in optimizing the therapeutic potential of bisbenzoxazole derivatives.

### 2.3 Detection of Apoptosis in MCF‐7 and HDFa Cells via Flow Cytometry Using Annexin V/Propidium Iodide Staining

Apoptosis, a form of programmed cell death triggered by both internal and external signals, is a critical target in cancer therapy. Strategies utilizing nanoparticles, drug precursor syntheses, and novel therapeutics aim to induce apoptosis in cancer cells or reduce the presence of malignant cells. In this context, we conducted a preliminary in vitro cytotoxicity study on 20 pyrrole‐tethered bisbenzoxazole derivatives, determining the IC_50_ values and exposure times for three selected compounds at their lowest concentrations.

**FIGURE 6:**
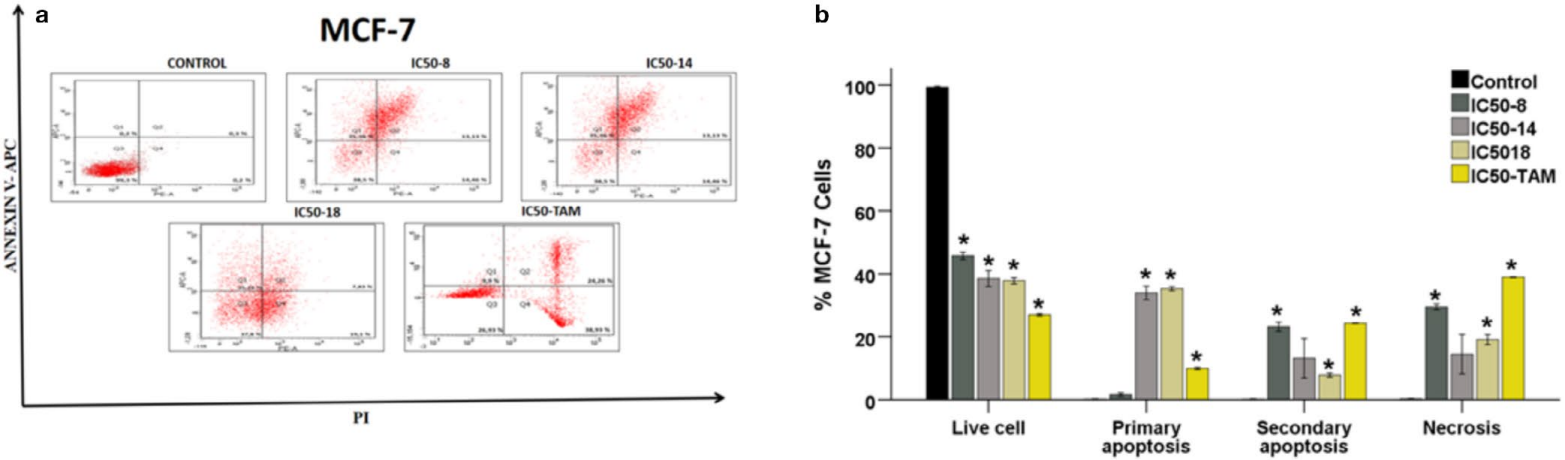
(a) Apoptosis and necrosis analysis of MCF‐7 cells was evaluated in flow cytometry after therapy for 48 h. Apoptosis data; live cells (Q3), early apoptosis (Q1), late apoptosis (Q2), and necrosis (Q4) populations were identified by plotting Annexin V staining intensity versus PI staining intensity. (b) Mean values of live cells, primary apoptosis, secondary apoptosis, and necrosis for % MCF‐7 Cells. Statistical significance values: When compared to the Control: *p ≤ 0.05.

The results indicated that compounds **B8**, **B14**, and **B18** demonstrated the highest efficacy at the 48‐h mark, with IC_50_ values of 1.89, 0.95, and 1.30 μM, respectively. Flow cytometry analysis was performed to evaluate the percentages of apoptosis for these compounds compared to tamoxifen. MCF‐7 cell suspensions treated with each compound for 48 h were stained using an apoptosis detection kit and subsequently analyzed using flow cytometry. Analysis of the flow cytometry results revealed a significant reduction in the percentage of viable cells across all experimental groups compared to the control group (*p* < 0.05 for all comparisons). Notably, the percentage of primary apoptosis in MCF‐7 cells significantly increased in all treated groups relative to the control (*p* < 0.05). Furthermore, secondary apoptosis was significantly elevated in the groups treated with Compound **B8**, **B18**, and tamoxifen compared to the control (*p* < 0.05). In contrast, necrosis percentages exhibited significant changes in all groups except for the **B14** group when compared to the control (*p* < 0.05) (Figure 6a,b).

It is important to note that effective cancer therapeutics should induce cell death primarily through apoptosis rather than necrosis. The PTB derivatives demonstrated a capacity to increase apoptosis rates in cancer cells while decreasing necrosis percentages relative to tamoxifen. These findings suggest that these derivatives effectively inhibit cancer cell proliferation by promoting higher rates of apoptosis.

**FIGURE 7:**
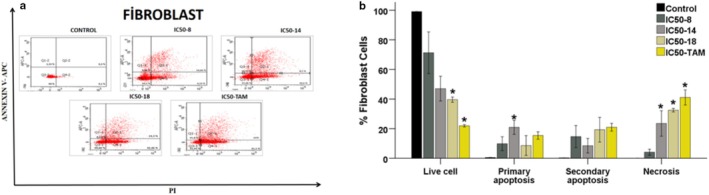
(a) Apoptosis and necrosis analysis of Fibroblast cells (HDF) was evaluated in flow cytometry after therapy for 48 h. Apoptosis data; live cells (Q3), early apoptosis (Q1), late apoptosis (Q2), and necrosis (Q4) populations were identified by plotting Annexin V staining intensity versus PI staining intensity. (b) Mean values of live cells, primary apoptosis, secondary apoptosis, and necrosis for % Fibroblast Cells. Statistical significance values: When compared to the Control: *p ≤ 0.05.

Subsequently, flow cytometry analysis was conducted on HDFa cells. Cells were treated with the same concentrations of Compound **B8**, **B14**, and **B18**, as well as Tamoxifen, for 48 h. Following staining with the apoptosis detection kit and analysis via flow cytometry (Figure 7a,b), results indicated a significant decrease in the average number of viable cells in the tamoxifen and **B18** treatment groups compared to the control. Additionally, a notable increase in the percentage of necrosis was observed in these groups (*p* < 0.05 for all comparisons). Importantly, the IC_50_ values of the PTB derivatives were found to be less damaging to healthy cells compared to tamoxifen. These results support the potential of these derivatives as promising anticancer agents with reduced toxicity to normal cells.

### 2.4 Demonstration of Cell Cycle Phases in MCF‐7 and HDFa Cells by Flow Cytometry Using Propidium Iodide

In the flow cytometry analysis, cell cycle kits facilitated the removal of cytoskeletal and nuclear proteins by disrupting lipid components of the cell membrane using detergents. This process resulted in the fragmentation of RNA by enzymatic activity. Consequently, we were able to identify abnormal DNA distributions and cell cycle phase profiles in the MCF‐7 breast cancer cell line.

**FIGURE 8:**
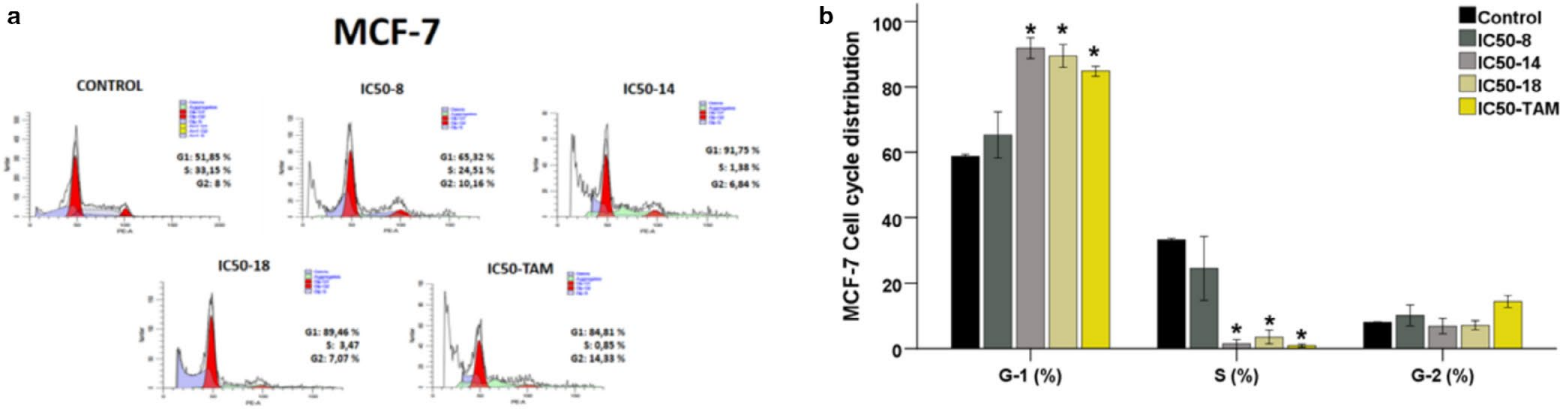
(a) Cell‐cycle analysis of MCF‐7 cells was evaluated in flow cytometry after therapy for 48 h. (b) Mean values of %MCF‐7 Cell cycle distribution. Statistical significance values: When compared to the Control: *p ≤ 0.05.

MCF‐7 cells were seeded in a 6‐well plate at a density of 1 × 10^6^ cells and incubated for 24 h. Following incubation, the culture medium was replaced, and the cells were treated for 48 h with **B8** (IC_50_ = 1.89 μM), **B14** (IC_50_ = 0.95 μM), **B18** (IC_50_ = 1.30 μM), and Tamoxifen (IC_50_ = 8.03 μM). MCF‐7 cells were then trypsinized, stained, and analyzed using a BD FACSAria III flow cytometer (BD Biosciences, Bedford, MA, USA) with FACS Diva Software and ModFit LT. Analysis of the flow cytometry data revealed that, when comparing the average cell cycle distributions of the **B14**, **B18**, and Tamoxifen treatment groups to the control group, there was a significant increase in the G1 phase and a corresponding decrease in the S phase (*p* < 0.05 for all comparisons). No significant changes were observed in the G2 phase averages (Figure 8a,b).

These findings suggest that while the **B14**, **B18**, and Tamoxifen treatment groups do not impact normal cell division, they may inhibit cell proliferation in breast cancer cells by inducing cell cycle arrest in the G1 phase.

### 2.5 Q‐PCR Analysis

**FIGURE 9:**
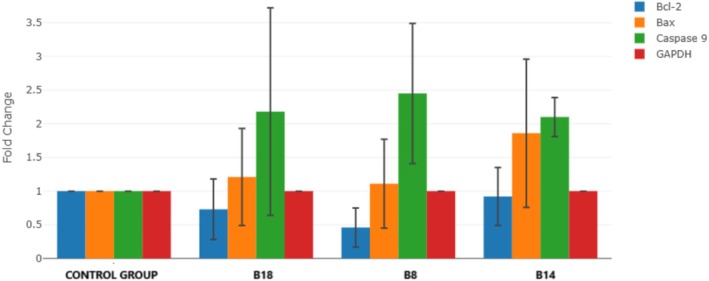
Gene expression levels in breast cancer with MCF‐7 cells. Statistical analyses and graphics were performed with GraphPad Prism 8.4.3 software. RT‐qPCR expression data analysis used the ∆∆*C*_t_ (∆∆Cq) method with calculations performed using the Data Analysis Center provided by Qiagen (2024). The *p*‐values for each gene in both control and experimental groups were determined using Student's *t*‐test.

Apoptosis genes Bcl‐2, Bax, TP53, and Caspase‐9 gene levels were analyzed in breast cancer cell lines (MCF‐7 cell lines) (Figure 9).

The gene expression levels of the three compounds with high IC_50_ values were examined, and it was observed that all three compounds were active in the caspase‐9 pathway. The synthesized compounds were found to be effective for breast cancer, and these compounds act by activating the caspase‐9 pathway.

## 3 Experimental Section

### 3.1 Chemistry

#### 3.1.1 General

All solvents, reactants, and catalysts used in this study are of analytical purity. As synthesis starting materials, 2‐aminophenol (Fluka), 2‐amino‐4‐chlorophenol (Abcr), 2‐amino‐4‐methylphenol (Acros), 2‐amino‐4‐methoxyphenol (Abcr), 2‐amino‐5‐methylphenol (Abcr), 4‐amino‐3‐hydroxy‐benzoic acid (Acros), and phosphoryl chloride (Merck) were used. As solvents, catalysts, and co‐reactants, methanol, ethanol, H_2_SO_4_ (Sulfuric acid, 98%), 1,4‐dioxane, acetic acid, THF (tetrahydrofuran), DCM (dichloromethane), 1,4‐dioxane, DMF (N'N′‐dimethylformamide), NaCN (sodium cyanide), n‐hexane, ethyl acetate, anhydrous MgSO_4_ (magnesium sulfate), and NaHCO_3_ (sodium bicarbonate) were used. ^1^H‐ and ^13^C‐NMR spectra were recorded on a Varian‐Agilent Inova instrument (400 and 100 MHz, respectively) using Me_4_Si as the internal standard. Melting points were determined on a Stuart Melting Point (SMP30) analyzer using open glass capillaries. Column chromatography was performed on silica gel (60 mesh, Silycycle). Commercially available materials were used without further purification. The analyses of the C, H, and N elements of the compounds were made with the LECO 932 CHNS (St. Joseph, MI, USA) elemental analysis device. Analysis results have a maximum deviation of ±0.4 from the calculated theoretical values.

#### 3.1.2 Synthesis of 5/6‐Substituted Benzoxazole‐2‐(Pyrrole‐2‐Il) Derivatives

(4/5)‐Substituted‐2‐aminophenol derivatives (**a–f**) (5 mmol) were dissolved in 10 mL of ethanol. A solution of 7 mmol 2‐pyrrolecarboxaldehyde in 5 mL of ethanol was added dropwise to the main reaction solution. The reaction medium was stirred at room temperature for 6 h. After it was determined by TLC that the starting material was completely converted into the product, the reaction was terminated. The obtained crude product (**17a–f**) was dissolved in 10 mL DMF after the solvent was evaporated, and a catalytic amount of NaCN was added. It was stirred at room temperature for approximately 4 h. The reaction was terminated after being controlled by the TLC method. After the reaction was completed, it was extracted with 3 × 15 mL ethyl acetate and 30 mL water and purified by column chromatography in the appropriate n‐hexane/ethyl acetate mobile phase (Kuzu, Hepokur, Alagoz, et al. 2022; Kuzu, Hepokur, Turkmenoglu, et al. 2022).

#### 3.1.3 Synthesis of 5‐(5/6‐Substituted‐Benzo[d]Oxazol‐2‐Yl)‐1H‐Pyrrole‐2‐Carbaldehyde Derivatives

5/6‐substituted benzoxazol‐2‐(pyrrol‐2‐yl) derivatives (**18a–f**) (1 mmol) obtained in the previous step were dissolved in 4 mL of dry DMF, and 1.2 mmol of phosphoryl chloride (POCl_3_) was added. It has been determined that pyrrole C‐5 carbonylation occurs when the reaction temperature is at reflux level. The reaction, optimized by preliminary experiments, was heated under reflux for 12 h and then neutralized with NaOH. The reaction solution was extracted with 3 × 20 mL ethyl acetate, 50 mL of water, and used without purification for the next step.

#### 3.1.4 Synthesis of Target Pyrrol‐Tethered Bisbenzoxazole Derivatives

5‐(5/6‐substituted‐benzo[d]oxazol‐2‐yl)‐1H‐pyrrole‐2‐carbaldehyde derivatives (**19a–f**) (1 mmol) obtained in the previous step were dissolved in 5 mL ethanol. A solution of 1 mmol of 4/5‐substituted‐2‐aminophenol derivatives (**a–f**) in 5 mL of ethanol was added dropwise to the main reaction solution. The reaction medium was stirred at room temperature for 6 h. After it was determined by TLC that the starting material was completely converted into the product, the reaction was terminated. After removing the solvent from the obtained crude product (**20a–f**), it was dissolved in 10 mL DMF, and a catalytic amount of NaCN was added. It was stirred at room temperature for approximately 4 h. The reaction was terminated after being controlled by the TLC method. After the reaction was completed, it was extracted with 3 × 15 mL ethyl acetate and 30 mL water and purified with the help of column chromatography in the appropriate n‐hexane/ethyl acetate mobile phase.

##### 2,5‐Bis(Benzo[d]Oxazol‐2‐Yl)‐1H‐Pyrrole (B1)

Yellow solid, M.p.: 208°C–209°C, Yield: 81%. ^1^H NMR (400 MHz, CDCl_3_) *δ* 10.70 (bs, 1H, ‐NH), 7.71–7.66 (m, 2H, Ar‐H), 7.51–7.47 (m, 2H, Ar‐H), 7.35–7.30 (m, 4H, Ar‐H), 7.16 (d, *J* = 2.5 Hz, 2H, Ar‐H). ^13^C NMR (100 MHz, CDCl_3_) *δ* 156.5, 150.3, 141.8, 125.0, 124.8, 123.6, 119.7, 114.1, 110.4. Anal. calcd. for C_18_H_11_N_3_O_2_: C: 71.75; H: 3.68; N: 13.95; Found: C: 71.79; H: 3.65; N: 13.91.

##### 2,5‐Bis(5‐Methylbenzo[d]Oxazol‐2‐Yl)‐1H‐Pyrrole (B2)

Light yellow solid, M.p.: 126°C–127°C, Yield: 89%. ^1^H NMR (400 MHz, CDCl_3_) *δ* 10.56 (bs, 1H, ‐NH), 7.48–7.44 (m, 2H, Ar‐H), 7.36 (d, *J* = 8.3 Hz, 2H, Ar‐H), 7.14–7.09 (m, 4H, Ar‐H), 2.46 (s, 6H, ‐CH_3_(x2)). ^13^C NMR (100 MHz, CDCl_3_) *δ* 156.6, 148.5, 141.9, 134.6, 126.0, 123.6, 119.5, 113.9, 109.7, 21.4. Anal. calcd. for C_20_H_15_N_3_O_2_: C: 72.94; H: 4.59; N: 12.76; Found: C: 72.89; H: 4.63; N: 12.80.

##### 2,5‐Bis(6‐Methylbenzo[d]Oxazol‐2‐Yl)‐1H‐Pyrrole (B3)

Light yellow solid, M.p.: 207°C–208°C, Yield: 92%. ^1^H NMR (400 MHz, CDCl_3_) *δ* 10.43 (bs, 1H, ‐NH), 7.56 (d, *J* = 8.1 Hz, 2H, Ar‐H), 7.32 (m, 2H, Ar‐H), 7.17–7.13 (m, 2H, Ar‐H), 7.12 (d, *J* = 2.5 Hz, 2H, Ar‐H), 2.50 (s, 6H, ‐CH_3_(x2)). ^13^C NMR (100 MHz, CDCl_3_) *δ* 156.0, 150.5, 139.5, 135.5, 126.0, 123.5, 118.9, 113.8, 110.6, 21.8. Anal. calcd. for C_20_H_15_N_3_O_2_: C: 72.95; H: 4.60; N: 12.78; Found: C: 72.90; H: 4.65; N: 12.83.

##### 2,5‐Bis(5‐Methoxybenzo[d]Oxazol‐2‐Yl)‐1H‐Pyrrole (B4)

Green solid, M.p.: 155°C–156°C, Yield: 85%. ^1^H NMR (400 MHz, CDCl_3_) *δ* 10.43 (bs, 1H, ‐NH), 7.39 (d, *J* = 8.8 Hz, 2H, Ar‐H), 7.17 (d, *J* = 2.5 Hz, 2H, Ar‐H), 7.17 (d, *J* = 2.5 Hz, 2H, Ar‐H), 6.92 (dd, *J* = 2.5 Hz, *J* = 8.8 Hz, 2H, Ar‐H), 3.86 (s, 6H, ‐OCH_3_(x2)). ^13^C NMR (100 MHz, CDCl_3_) *δ* 157.6, 157.2, 144.9, 142.6, 123.6, 113.9, 113.4, 110.5, 102.7, 55.9. Anal. calcd. for C_20_H_15_N_3_O_4_: C: 66.48; H: 4.38; N: 12.17; Found: C: 66.72; H: 4.35; N: 12.13.

##### 2,5‐Bis(5‐Chlorobenzo[d]Oxazol‐2‐Yl)‐1H‐Pyrrole (B5)

White solid, M.p.: 277°C–278°C, Yield: 93%. ^1^H NMR (400 MHz, CDCl_3_) *δ* 10.33 (bs, 1H, ‐NH), 7.70 (dd, *J* = 0.5 Hz, *J* = 2.1 Hz, 2H, Ar‐H), 7.48 (dd, *J* = 0.5 Hz, *J* = 8.6 Hz, 2H, Ar‐H), 7.32 (dd, *J* = 2.1 Hz, *J* = 8.6 Hz, 2H, Ar‐H), 7.2 (d, *J* = 2.5 Hz, 2H, Ar‐H). ^13^C NMR (100 MHz, CDCl_3_) *δ* 157.5, 148.9, 142.9, 130.4, 125.4, 123.4, 119.7, 114.6, 111.2. Anal. calcd. for C_18_H_9_Cl_2_N_3_O_2_: C: 58.40; H: 2.45; N: 11.35; Found: C: 58.37; H: 2.51; N: 11.40.

##### 2‐(5‐(Benzo[d]Oxazol‐2‐Yl)‐1H‐Pyrrol‐2‐Yl)‐5‐Methylbenzo[d]Oxazole (B6)

Light brown solid, M.p.: 168°C–169°C, Yield: 94%. ^1^H NMR (400 MHz, CDCl_3_) *δ* 10.59 (bs, 1H, ‐NH), 7.71–7.66 (m, 1H, Ar‐H), 7.51–7.48 (m, 1H, Ar‐H), 7.47–7.45 (m, 1H, Ar‐H), 7.36 (d, *J* = 8.2 Hz, 1H, Ar‐H), 7.34–7.30 (m, 2H, Ar‐H), 7.17–7.10 (m, 3H, Ar‐H), 2.46 (s, 3H, ‐CH_3_). ^13^C NMR (100 MHz, CDCl_3_) *δ* 156.6, 156.5, 150.3, 148.5, 141.9, 141.8, 134.7, 126.1, 125.0, 124.8, 123.8, 123.4, 119.6, 119.5, 114.1, 113.9, 110.4, 109.7, 21.5. Anal. calcd. for C_19_H_13_N_3_O_2_: C: 72.37; H: 4.16; N: 13.33; Found: C: 72.41; H: 4.20; N: 13.28.

##### 2‐(5‐(Benzo[d]Oxazol‐2‐Yl)‐1H‐Pyrrol‐2‐Yl)‐6‐Methylbenzo[d]Oxazole (B7)

Light yellow solid, M.p.: 211°C–212°C, Yield: 83%. ^1^H NMR (400 MHz, CDCl_3_) *δ* 10.56 (bs, 1H, ‐NH), 7.71–7.67 (m, 1H, Ar‐H), 7.55 (dd, *J* = 8.1 Hz, 1H, Ar‐H), 7.52–7.48 (m, 1H, Ar‐H), 7.34–7.31 (m, 2H, Ar‐H), 7.30–7.28 (m, 1H, Ar‐H), 7.16–7.12 (m, 3H, Ar‐H), 2.49 (s, 3H, ‐CH_3_). ^13^C NMR (100 MHz, CDCl_3_) *δ* 156.5, 156.0, 150.5, 150.2, 141.8, 139.6, 135.5, 126.0, 124.9, 124.8, 123.8, 123.3, 119.6, 119.0, 114.1, 113.8, 110.6, 110.4, 21.7. Anal. calcd. for C_19_H_13_N_3_O_2_: C: 72.37; H: 4.16; N: 13.33; Found: C: 72.42; H: 4.24; N: 13.35.

##### 2‐(5‐(Benzo[d]Oxazol‐2‐Yl)‐1H‐Pyrrol‐2‐Yl)‐5‐Methoxybenzo[d]Oxazole (B8)

Light yellow solid, M.p.: 165°C–166°C, Yield: 86%. ^1^H NMR (400 MHz, CDCl_3_) *δ* 10.74 (bs, 1H, ‐NH), 7.71–7.67 (m, 1H, Ar‐H), 7.53–7.49 (m, 1H, Ar‐H), 7.38 (d, *J* = 8.8 Hz, 1H, Ar‐H), 7.35–7.30 (m, 2H, Ar‐H), 7.18–7.10 (m, 3H, Ar‐H), 6.91 (dd, *J* = 2.5 Hz, *J* = 8.8 Hz, 1H, Ar‐H), 3.85 (s, 3H, ‐OCH_3_). ^13^C NMR (100 MHz, CDCl_3_) *δ* 157.6, 157.2, 156.5, 150.2, 144.9, 142.6, 141.7, 125.0, 124.8, 123.7, 123.5, 119.6, 114.2, 113.9, 113.5, 110.5, 110.4, 102.7, 55.9. Anal. calcd. for C_19_H_13_N_3_O_3_: C: 68.88; H: 3.95; N: 12.68; Found: C: 68.93; H: 4.01; N: 12.65.

##### 2‐(5‐(Benzo[d]Oxazol‐2‐Yl)‐1H‐Pyrrol‐2‐Yl)‐5‐Chlorobenzo[d]Oxazole (B9)

Yellow solid, M.p.: 222°C–223°C, Yield: 75%. ^1^H NMR (400 MHz, CDCl_3_) *δ* 10.41 (bs, 1H, ‐NH), 7.73–7.69 (m, 1H, Ar‐H), 7.68–7.65 (m, 1H, Ar‐H), 7.56–7.52 (m, 1H, Ar‐H), 7.43 (dd, *J* = 0.5 Hz, *J* = 8.6 Hz, 1H, Ar‐H), 7.37–7.34 (m, 2H, Ar‐H), 7.30 (dd, *J* = 2.1 Hz, *J* = 8.6 Hz, 1H, Ar‐H), 7.16 (d, *J* = 2.6 Hz, 2H, Ar‐H). ^13^C NMR (100 MHz, CDCl_3_) *δ* 157.6, 156.3, 150.3, 148.9, 143.0, 141.6, 130.3, 125.2, 125.1, 124.9, 123.9, 123.0, 119.7, 119.6, 114.6, 114.2, 111.1, 110.5. Anal. calcd. for C_18_H_10_ClN_3_O_2_: C: 64.39; H: 3.00; N: 12.52; Found: C: 64.36; H: 3.04; N: 12.58.

##### Methyl 2‐(5‐(Benzo[d]Oxazol‐2‐Yl)‐1H‐Pyrrol‐2‐Yl)benzo[d]Oxazole‐6‐Carboxylate (B10)

White solid, M.p.: 220°C–221°C, Yield: 69%. ^1^H NMR (400 MHz, CDCl_3_) *δ* 10.58 (bs, 1H, ‐NH), 8.20–8.18 (m, 1H, Ar‐H), 8.08 (dd, *J* = 1.5 Hz, *J* = 8.3 Hz, 1H, Ar‐H), 7.73–7.68 (m, 2H, Ar‐H), 7.57–7.52 (m, 1H, Ar‐H), 7.37–7.33 (m, 2H, Ar‐H), 7.22 (dd, *J* = 2.5 Hz, *J* = 4.0 Hz, 1H, Ar‐H), 7.17 (dd, *J* = 2.5 Hz, *J* = 4.0 Hz, 1H, Ar‐H), 3.97 (s, 3H, ‐COOMe). ^13^C NMR (100 MHz, CDCl_3_) *δ* 166.50, 158.7, 156.2, 150.3, 150.0, 145.8, 141.6, 127.0, 126.7, 125.2, 124.9, 124.3, 123.0, 119.7, 119.1, 115.2, 114.3, 112.0, 110.5, 52.3. Anal. calcd. for C_20_H_13_N_3_O_4_: C: 66.85; H: 3.65; N: 11.69; Found: C: 66.90; H: 3.59; N: 11.73.

##### 6‐Methyl‐2‐(5‐(5‐Methylbenzo[d]Oxazol‐2‐Yl)‐1H‐Pyrrol‐2‐Yl)benzo[d]Oxazole (B11)

Light brown solid, M.p.: 185°C–187°C, Yield: 90%. ^1^H NMR (400 MHz, CDCl_3_) *δ* 10.70 (bs, 1H, ‐NH), 7.52 (d, *J* = 8.2 Hz, 1H, Ar‐H), 7.45–7.42 (m, 1H, Ar‐H), 7.32 (d, *J* = 8.2 Hz, 1H, Ar‐H), 7.25–7.23 (m, 1H, Ar‐H), 7.12–7.08 (m, 4H, Ar‐H), 2.47 (s, 3H, ‐CH_3_), 2.43 (s, 3H, ‐CH_3_). ^13^C NMR (100 MHz, CDCl_3_) *δ* 156.6, 156.0, 150.5, 148.5, 142.0, 139.6, 135.4, 134.6, 126.0, 125.9, 123.7, 123.5, 119.5, 118.9, 113.9, 113.7, 110.5, 109.7, 21.7, 21.4. Anal. calcd. for C_20_H_15_N_3_O_2_: C: 72.95; H: 4.60; N: 12.78; Found: C: 72.91; H: 4.66; N: 12.82.

##### 5‐Methoxy‐2‐(5‐(5‐Methylbenzo[d]Oxazol‐2‐Yl)‐1H‐Pyrrol‐2‐Yl)benzo[d]Oxazole (B12)

Dark yellow solid, M.p.: 133°C–134°C, Yield: 88%. ^1^H NMR (400 MHz, CDCl_3_) *δ* 10.43 (bs, 1H, ‐NH), 7.49–7.46 (m, 1H, Ar‐H), 7.39 (d, *J* = 8.8 Hz, 2H, Ar‐H), 7.18 (d, *J* = 2.5 Hz, 1H, Ar‐H), 7.15–7.11 (m, 3H, Ar‐H), 6.92 (dd, *J* = 2.5 Hz, *J* = 8.8 Hz, 1H, Ar‐H), 3.86 (s, 3H, ‐OCH_3_), 2.47 (s, 3H, ‐CH_3_). ^13^C NMR (100 MHz, CDCl_3_) *δ* 157.6, 157.2, 156.5, 148.5, 144.9, 142.7, 142.0, 134.7, 126.1, 123.6, 123.5, 119.6, 113.9, 113.8, 113.4, 110.5, 109.8, 102.7, 55.9, 21.5. Anal. calcd. for C_20_H_15_N_3_O_3_: C: 69.56; H: 4.38; N: 12.17; Found: C: 69.60; H: 4.42; N: 12.21.

##### 5‐Chloro‐2‐(5‐(5‐Methylbenzo[d]Oxazol‐2‐Yl)‐1H‐Pyrrol‐2‐Yl)benzo[d]Oxazole (B13)

White solid, M.p.: 224°C–225°C, Yield: 87%. ^1^H NMR (400 MHz, CDCl_3_) *δ* 10.43 (bs, 1H, ‐NH), 7.65 (d, *J* = 2.1 Hz, 1H, Ar‐H), 7.48–7.46 (m, 1H, Ar‐H), 7.40 (t, *J* = 8.0 Hz, 2H, Ar‐H), 7.28 (dd, *J* = 2.1 Hz, *J* = 8.6 Hz, 1H, Ar‐H), 7.15–7.11 (m, 3H, Ar‐H), 2.47 (s, 3H, ‐CH_3_). ^13^C NMR (100 MHz, CDCl_3_) *δ* 157.7, 156.3, 148.9, 148.5, 143.0, 141.8, 134.8, 130.3, 126.3, 125.2, 124.2, 122.9, 119.6, 119.5, 114.6, 113.9, 111.0, 109.8, 21.5. Anal. calcd. for C_19_H_12_ClN_3_O_2_: C: 65.24; H: 3.46; N: 12.01; Found: C: 65.29; H: 3.51; N: 11.98.

##### Methyl 2‐(5‐(5‐Methylbenzo[d]Oxazol‐2‐Yl)‐1H‐Pyrrol‐2‐Yl)benzo[d]Oxazole‐6‐Carboxylate (B14)

White solid, M.p.: 215°C–217°C, Yield: 74%. ^1^H NMR (400 MHz, CDCl_3_) *δ* 10.59 (bs, 1H, ‐NH), 8.17 (dd, *J* = 0.6 Hz, *J* = 1.5 Hz, 1H, Ar‐H), 8.06 (dd, *J* = 1.5 Hz, *J* = 8.3 Hz, 1H, Ar‐H), 7.68 (dd, *J* = 0.6 Hz, *J* = 8.3 Hz, 1H, Ar‐H), 7.49–7.46 (m, 1H, Ar‐H), 7.39 (d, *J* = 8.3 Hz, 1H, Ar‐H), 7.21–7.18 (m, 1H, Ar‐H), 7.16–7.11 (m, 2H, Ar‐H), 3.96 (s, 3H, ‐COOMe), 2.46 (s, 3H, ‐CH_3_). ^13^C NMR (100 MHz, CDCl_3_) *δ* 166.5, 158.8, 156.3, 149.9, 148.5, 145.8, 141.8, 134.8, 126.9, 126.6, 126.3, 124.5, 122.8, 119.6, 119.0, 115.1, 114.0, 111.9, 109.8, 52.3, 21.4. Anal. calcd. for C_21_H_15_N_3_O_4_: C: 67.56; H: 4.05; N: 12.25; Found: C: 67.61; H: 4.09; N: 12.20.

##### 5‐Methoxy‐2‐(5‐(6‐Methylbenzo[d]Oxazol‐2‐Yl)‐1H‐Pyrrol‐2‐Yl)benzo[d]Oxazole (B15)

Purple solid, M.p.: 148°C, Yield: 89%. ^1^H NMR (400 MHz, CDCl_3_) *δ* 10.76 (bs, 1H, ‐NH), 7.55 (d, *J* = 8.1 Hz, 1H, Ar‐H), 7.38 (d, *J* = 8.8 Hz, 1H, Ar‐H), 7.32–7.29 (m, 1H, Ar‐H), 7.17–7.11 (m, 4H, Ar‐H), 6.91 (dd, *J* = 2.5 Hz, *J* = 8.8 Hz, 1H, Ar‐H), 3.86 (s, 3H, ‐OMe), 2.49 (s, 3H, ‐CH_3_). ^13^C NMR (100 MHz, CDCl_3_) *δ* 157.6, 157.3, 156.1, 150.5, 144.9, 142.6, 139.5, 135.5, 126.0, 123.7, 123.5, 118.9, 114.0, 113.9, 113.4, 110.6, 110.5, 102.7, 55.9, 29.7. Anal. calcd. for C_20_H_15_N_3_O_3_: C: 69.56; H: 4.38; N: 12.17; Found: C: 69.62; H: 4.40; N: 12.19.

##### Methyl 2‐(5‐(6‐Methylbenzo[d]Oxazol‐2‐Yl)‐1H‐Pyrrol‐2‐Yl)benzo[d]Oxazole‐6‐Carboxylate (B16)

White solid, M.p.: 238°C–239°C, Yield: 87%. ^1^H NMR (400 MHz, CDCl_3_) *δ* 10.54 (bs, 1H, ‐NH), 8.19 (dd, *J* = 0.6 Hz, *J* = 1.5 Hz, 1H, Ar‐H), 8.08 (dd, *J* = 1.5 Hz, *J* = 8.3 Hz, 1H, Ar‐H), 7.70 (dd, *J* = 0.6 Hz, *J* = 8.3 Hz, 1H, Ar‐H), 7.58 (d, *J* = 8.1 Hz, 1H, Ar‐H), 7.34 (dt, *J* = 0.6 Hz, *J* = 1.4 Hz, 1H, Ar‐H), 7.21 (dd, *J* = 2.5 Hz, *J* = 4.0 Hz, 1H, Ar‐H), 7.17 (ddd, *J* = 0.6 Hz, *J* = 1.5 Hz, *J* = 8.1 Hz, 1H, Ar‐H), 7.14 (dd, *J* = 2.5 Hz, *J* = 4.0 Hz, 1H, Ar‐H), 3.97 (s, 3H, ‐COOMe), 2.50 (s, 3H, ‐CH_3_). ^13^C NMR (100 MHz, CDCl_3_) *δ* 166.5, 158.8, 155.8, 150.6, 150.0, 145.8, 139.4, 135.8, 126.9, 126.7, 126.1, 124.5, 122.7, 119.0, 115.1, 113.9, 112.0, 110.7, 52.3, 30.9. Anal. calcd. for C_21_H_15_N_3_O_4_: C: 67.56; H: 4.05; N: 12.25; Found: C: 67.58; H: 4.01; N: 12.29.

##### 5‐Chloro‐2‐(5‐(5‐Methoxybenzo[d]Oxazol‐2‐Yl)‐1H‐Pyrrol‐2‐Yl)benzo[d]Oxazole (B17)

Light yellow solid, M.p.: 204°C–205°C, Yield: 89%. ^1^H NMR (400 MHz, CDCl_3_) *δ* 10.53 (bs, 1H, ‐NH), 7.65 (dd, *J* = 0.4 Hz, *J* = 2.1 Hz, 1H, Ar‐H), 7.41 (t, *J* = 8.6 Hz, 2H, Ar‐H), 7.28 (dd, *J* = 2.1 Hz, *J* = 8.6 Hz, 1H, Ar‐H), 7.17 (d, *J* = 2.5 Hz, 1H, Ar‐H), 7.15–7.10 (m, 2H, Ar‐H), 6.92 (dd, *J* = 2.6 Hz, *J* = 8.6 Hz, 1H, Ar‐H), 3.86 (s, 3H, ‐OCH_3_). ^13^C NMR (100 MHz, CDCl_3_) *δ* 157.7, 157.6, 157.0, 148.9, 144.9, 143.0, 142.6, 130.3, 125.2, 124.1, 122.9, 119.6, 114.6, 113.9, 113.6, 111.0, 110.5, 102.7, 55.9. Anal. calcd. for C_19_H_12_ClN_3_O_3_: C: 62.39; H: 3.31; N: 11.49; Found: C: 62.43; H: 3.32; N: 11.54.

##### Methyl 2‐(5‐(5‐Methoxybenzo[d]Oxazol‐2‐Yl)‐1H‐Pyrrol‐2‐Yl)benzo[d]Oxazole‐6‐Carboxylate (B18)

Light yellow solid, M.p.: 240°C–241°C, Yield: 81%. ^1^H NMR (400 MHz, *d*_*6*_‐DMSO) *δ* 8.22–8.20 (m, 1H, Ar‐H), 8.01 (dd, *J* = 1.6 Hz, *J* = 8.3 Hz, 1H, Ar‐H), 7.84 (d, *J* = 8.3 Hz, 1H, Ar‐H), 7.63 (d, *J* = 8.9 Hz, 1H, Ar‐H), 7.29 (d, *J* = 2.5 Hz, 1H, Ar‐H), 7.23 (d, *J* = 4.0 Hz, 1H, Ar‐H), 7.14 (d, *J* = 4.0 Hz, 1H, Ar‐H), 6.98 (dd, *J* = 2.5 Hz, *J* = 8.9 Hz, 1H, Ar‐H), 3.89 (s, 3H, ‐COOMe), 3.82 (s, 3H, ‐OMe). ^13^C NMR (100 MHz, *d*_*6*_‐DMSO) *δ* 166.3, 159.4, 157.7, 157.4, 149.9, 146.2, 144.7, 142.9, 126.7, 126.6, 125.1, 123.4, 119.6, 116.2, 115.0, 113.8, 111.9, 111.3, 103.3, 56.3, 52.9. Anal. calcd. for C_21_H_15_N_3_O_5_: C: 64.78; H: 3.88; N: 10.79; Found: C: 64.83; H: 3.92; N: 10.80.

##### 5‐Chloro‐2‐(5‐(6‐Methylbenzo[d]Oxazol‐2‐Yl)‐1H‐Pyrrol‐2‐Yl)benzo[d]Oxazole (B19)

Light yellow solid, M.p.: 227°C–228°C, Yield: 79%. ^1^H NMR (400 MHz, CDCl_3_) *δ* 10.36 (bs, 1H, ‐NH), 7.68–7.67 (m, 1H, Ar‐H), 7.58 (d, *J* = 8.1 Hz, 1H, Ar‐H), 7.46–7.43 (m, 1H, Ar‐H), 7.36–7.34 (m, 1H, Ar‐H), 7.30 (dd, *J* = 0.4 Hz, *J* = 8.6 Hz, 1H, Ar‐H), 7.19–7.16 (m, 1H, Ar‐H), 7.15–7.12 (m, 2H, Ar‐H), 2.51 (s, 3H, ‐CH_3_). ^13^C NMR (100 MHz, CDCl_3_) *δ* 157.7, 155.8, 150.6, 148.9, 143.0, 139.5, 135.8, 130.3, 126.1, 125.2, 124.2, 122.8, 119.6, 119.0, 114.6, 113.8, 111.1, 110.7, 21.8. Anal. calcd. for C_19_H_12_ClN_3_O_2_: C: 65.24; H: 3.46; N: 12.01; Found: C: 65.27; H: 3.52; N: 12.05.

##### Methyl 2‐(5‐(5‐Chlorobenzo[d]Oxazol‐2‐Yl)‐1H‐Pyrrol‐2‐Yl)benzo[d]Oxazole‐6‐Carboxylate (B20)

Yellow solid, M.p.: 257°C–259°C, Yield: 71%. ^1^H NMR (400 MHz, *d*_*6*_‐DMSO) *δ* 8.22–8.19 (m, 1H, Ar‐H), 8.01 (dd, *J* = 1.6 Hz, *J* = 8.4 Hz, 1H, Ar‐H), 7.87–7.82 (m, 2H, Ar‐H), 7.77 (d, *J* = 8.7 Hz, 1H, Ar‐H), 7.43 (dd, *J* = 2.1 Hz, *J* = 8.7 Hz, 1H, Ar‐H), 7.23 (d, *J* = 4.0 Hz, 1H, Ar‐H), 7.19 (d, *J* = 4.0 Hz, 1H, Ar‐H), 3.89 (s, 3H, ‐COOMe). ^13^C NMR (100 MHz, *d*_*6*_‐DMSO) *δ* 167.1, 166.2, 159.2, 158.1, 149.9, 149.0, 146.1, 143.3, 129.6, 126.8, 125.7, 124.4, 123.9, 119.7, 119.5, 116.2, 115.7, 112.5, 112.0, 52.9. Anal. calcd. for C_20_H_12_ClN_3_O_4_: C: 61.00; H: 3.07; N: 10.67; Found: C: 60.97; H: 3.10; N: 10.72.

### 3.2 Biological Experiments

#### 3.2.1 Materials and Methods

The human dermal fibroblast (HDFa) and human breast cancer cells (MCF‐7) were obtained from the American Type Culture Collection (ATCC; Manassas, VA, USA). Cells were cultured in RPMI medium (MCF‐7) and DMEM medium (HDFa) was supplemented with 10% FBS, 1% penicillin–streptomycin, 1% amphotericin, and 2.5% L‐glutamine at 37°C in a 5% CO_2_ incubator.

#### 3.2.2 Cell Viability

MTT Method. The MTT method is used to detect and quantify living organisms cultured with the method colorimetrically. This method relies on intact mitochondria cleaving the tetrazolium ring of the MTT dye. The MTT substance is absorbed and reduced to a colorful, water‐insoluble formazan by a mitochondria‐dependent reaction. Cell viability is measured based on the MTT reduction property, and the density of dye obtained from MTT analysis indicates the number of viable cells. In this study, cell viability was assessed using the MTT (0.5 mg/mL) solution that was added to each well. The IC_50_ value was calculated by applying the MTT cell proliferation method in the MCF‐7 (ATCC HTB‐22) cell line treated with 20 molecules of Pyrrole‐Tethered Bisbenzoxazole Derivatives (100 nM,1 μM, 10 μM, 50 μM, and 100 μM). After adding properties to cultures, they were kept at 37°C for 24 h and overnight. Then, at 48 and 72 h after applying the molecules, 10 μL of MTT was added to each well and incubated under similar conditions for 4 h. Absorbance spectrophotometry was performed at a wavelength of 570 nm using the IC_50_ = [(control absorbents‐sample absorbents)/control absorbents] × 100 calculation method (Khorsandi et al. 2017).

xCELLigence Real‐Time Cell Analysis. The xCELLigence system (Real‐Time Cell Analyzer (RTCA) Dual Plate (DP) (Roche Diagnostics GmbH, Penzberg)) was used for real‐time monitoring of cell viability without the need for labeling. A total of 1 × 10^4^ HDFa cells were seeded into each well of the E‐plate, and the cell proliferation curve was monitored for 24 h. After that, different IC_50_ values of derivatives **B8**, **B14**, **B18**, and Tamoxifen were added to the E‐plate system, and the cells were monitored in real‐time for 72 h. The Cell Index (CI) value was automatically measured every hour for each well using RTCA Software (version 1.2.1) (Yetkin et al. 2021; Yıldırım et al. 2022).

#### 3.2.3 Demonstration of Apoptosis Levels in MCF‐7 Breast Cancers and Fibroblast Cells by Flow Cytometry

The assessment of apoptosis through flow cytometry is a valuable method for obtaining quantitative results to determine the levels of apoptosis and necrosis in cells. In our study, the 48th‐hour apoptosis levels of three synthases with the lowest IC_50_ values (PTB derivatives **B8**, **B14**, and **B18**) were evaluated. To assess apoptosis levels, MCF‐7 cells and healthy fibroblast cells were incubated with these synthases for 48 h. After this incubation period, the cells were treated with Annexin‐V APC and PE propidium iodide (PI) dyes to examine apoptosis levels. Then, to examine the levels of apoptosis, the cells were incubated at a concentration of 2 × 10^5^ cells/mL with 5 μL Annexin‐V APC and 10 μL PI dyes in 100 μL Binding buffer for 15 min at room temperature. Samples were then washed twice in PBS, and the BD FACS ARIA III flow cytometry device was used to determine the percentage of apoptotic cells. (Ayaz et al. 2022).

#### 3.2.4 Determination of Cell Cycle Stages by Flow Cytometry

The cell cycle kit (MCE USA‐HY‐K1071‐50 T) works on the principle of dissolving the lipids in the cell membrane with the help of detergents. This kit was used to identify cells with abnormal DNA and cell cycle phase distributions in a breast cancer cell line. It was analyzed by removing the cytoskeleton and nuclear proteins with the help of trypsin and digesting the cellular RNA by enzymes. Cells were seeded at 1 × 10^6^ in a 6‐well plate and incubated for 24 h. IC_50_ concentrations were then applied to the cells and incubated for 48 h. Cells were removed by adding trypsin to adherent cells. They were centrifuged at 1000 g for 3–5 min at 4°C, and then the supernatant was discarded. One milliliter of prechilled PBS was added to resuspend the cells, centrifuged at 1000 g for 3–5 min at 4°C, and detected via BD FACSAria III flow cytometer (BD Biosciences, Bedford, MA, USA). Percentage data were obtained using FACS Diva Software and ModFit LT (Şimşek et al. 2020).

#### 3.2.5 RT‐PCR Test

Total RNA was isolated from MCF‐7 cells treated with (IC50 values) B8, 14, and 18. The RNeasy Mini Kit (QIAGEN, catalog no: 74104) was used for total RNA isolation. Cells treated with the vehicle (0.1% DMSO) for 48 h were collected as the control, and total RNA was isolated. In this study, the expressions of Bcl‐2, Bax, and caspase 3 genes in MCF‐7 cells treated with IC50 values of B8, 14, and 18 were analyzed using the RT‐PCR method. For the quantification of total RNA samples previously isolated from cell lines, the RNAs were analyzed using ultraviolet–visible spectroscopy (UV‐VIS) (Maestro NANO). Total RNA concentration (ng/μL) and purity (A260/A280) of all samples were evaluated. cDNA synthesis was performed from total RNA following the kit protocol (RNase Inh. High Capacity, catalog no: C03‐01‐20). To standardize the RNA concentration for the reverse transcription reaction, each sample stored at −80°C was adjusted to 100 ng/μL using nuclease‐free water. The cDNA synthesis reaction consisted of 2 μL of 10× reaction buffer, 1 μL of dNTP mixture, 2 μL of random hexamers, 1 μL of reverse transcriptase, 0.5 μL of RNase inhibitor, 3.5 μL of RNase‐free water, and 10 μL of total RNA, resulting in a final volume of 20 μL. This mixture was then subjected to incubation in the Bio‐Rad Thermo Cycler at 25°C for 10 min, 37°C for 120 min, and 85°C for 5 min. For the RT‐PCR reaction, the RT‐PCR SYBR‐Green MasterMix kit was used along with the following optimized primers: BCl‐2, Bax, TP53, and caspase‐3. The GAPDH gene was used as an internal control for gene expression analysis. For the RT‐PCR step, each reaction was replicated three times for both groups. Reaction components were prepared as follows: 6 μL of nuclease‐free water and 10 μL of SYBR Green Mastermix (A.B.T., Cat. No: Q03‐02‐01), 2 μL of cDNA, 1 μL of forward primer, and 1 μL of reverse primer. The reaction mixtures were plated and subjected to incubation using the Roche LightCycler 96 with the following protocol: Initial incubation for 5 min at 95°C for 1 cycle; 40 cycles of 15 s at 95°C and 1 min at 60°C for 2‐step amplification; Melting curve analysis was conducted in 1 cycle with 10 s at 95°C, 1 min at 65°C, and 97°C for 1 s to determine specificity. In the RT‐PCR step, reactions were repeated three times for both cell lines. The analysis of RT‐PCR expression data was based on the ∆∆*C*_t_ (∆∆Cq) method using the Data Analysis Center provided by Qiagen (2024).

## 4 Conclusion

In summary, the design and synthesis of PTB derivatives, inspired by both natural and synthetic molecular frameworks, have demonstrated remarkable anticancer potential against the MCF‐7 breast cancer cell line. Comparative analysis with tamoxifen revealed that compounds **B8**, **B14**, and **B18** exhibited significantly superior antiproliferative efficacy. Among these, the 6‐COOMe substituent emerged as a pivotal determinant of activity, leveraging its polar and electron‐withdrawing properties to facilitate strong interactions with target proteins through hydrogen bonding and electrostatic interactions.

The synergistic effect of the 5‐CH_3_ and 6‐COOMe groups, exemplified by compound **B14**, highlighted the importance of strategic substituent positioning, culminating in the lowest IC_50_ value among all synthesized derivatives. Mechanistic studies confirmed that these compounds effectively induced apoptosis in MCF‐7 cells by arresting the cell cycle at the G1 phase. Furthermore, gene expression analyses revealed significant upregulation of caspase 9 and key alterations in cancer‐related genes, including Bcl‐2, Bax, and GAPDH, implicating the caspase 9‐mediated apoptotic pathway as a central mechanism of action.

Overall, this study underscores the therapeutic potential of PTB derivatives as promising candidates for next‐generation anticancer agents. These findings not only highlight their efficacy in breast cancer treatment but also provide valuable insights into the design of targeted therapies that exploit apoptosis induction for enhanced clinical outcomes.

## Conflicts of Interest

The authors declare no conflicts of interest.

## Supporting information


Data S1.

## Data Availability

The data that supports the findings of this study are available in Data S1.
