# Supplementary material for: Pyrrole‐Tethered Bisbenzoxazole Derivatives: Apoptosis‐Inducing Agents Targeting Breast Cancer Cells
Source: Chem Biol Drug Des. 2025 Mar 13;105(3):e70078. doi: 10.1111/cbdd.70078 (PMC11905336; doi:10.1111/cbdd.70078)
Supplement: Supplementary file 1 — Data S1. [file CBDD-105-e70078-s001.doc]

**Pyrrole-Tethered Bisbenzoxazole Derivatives as Apoptosis-Inducing Agents for Breast Cancer Cells**

**Burak KUZU^1*^, Derya YETKİN^2^, Ceylan HEPOKUR^3^, Öztekin ALGÜL^4,5*^**

^1^Department of Pharmaceutical Chemistry, Faculty of Pharmacy, Van Yuzuncu Yil University, Van, 65080, Türkiye

^2^Advance Technology Education Research and Application Centre, Mersin University, Mersin, 33169, Türkiye

^3^Department of Biochemistry, Faculty of Pharmacy, Sivas Cumhuriyet University, Sivas, 58140, Türkiye

^4^Department of Pharmaceutical Chemistry, Faculty of Pharmacy, Mersin University, Mersin, 33169, Türkiye

^5^Department of Pharmaceutical Chemistry, Faculty of Pharmacy, Erzincan Binali Yildirim University, Erzincan, 24100, Türkiye

*Authors for correspondence: [burakkuzu@yyu.edu.tr](mailto:burakkuzu@yyu.edu.tr) and [oztekinalgul@mersin.edu.tr](mailto:oztekinalgul@mersin.edu.tr)

**Supplementary Material**

| **Contents** | **Pages** |
| --- | --- |
| 1. Synthesis of methyl 4-amino-3-hydroxybenzoate as a synthesis starting point | **2** |
| 1. Synthesis of pyrrole-2-carbaldehyde | **2** |
| 1. ^1^H- and ^13^C-NMR spectrum copies of compounds B1-B20 | **3** |
| 1. Analysis of apoptosis and necrosis of MCF-7 and fibroblast (HDF) cells (48h) | **23** |
| 1. Cell-cycle analysis of MCF-7 and fibroblast (HDF) cells (48h) | **24** |
| 1. A time-dependent graph of cell index values of B8 in MCF-7 | **25** |
| 1. A time-dependent plot of B8, 14, 18 and Tamoxifen IC_50_ values in HDFa cells at 72 hrs | **25** |
| 1. Table S1. Forward and Reverse Primer PCR Sequences for Real-time PCR | **26** |
| 1. References | **26** |

1. **Synthesis of methyl 4-amino-3-hydroxybenzoate**

The 4-amino-3-hydroxy-benzoic acid (5 mmol) was dissolved in 10 ml of methanol and refluxed for 12 hours under the catalyst of a few drops of concentrated H_2_SO_4_. After the completion of the reaction was checked with TLC, the reaction was terminated and cooled to room temperature. It was neutralized with 50 ml of water and 1N NaHCO_3_ until pH:7.5. The resulting aqueous phase was extracted with 3x15 ml of ethyl acetate. The organic phase was separated and the solvent was evaporated and purified by column chromatography in the appropriate n-hexane/ethylacetate (5/1) mobile phase.**^1^**

1. **Synthesis of pyrrole-2-carbaldehyde**

Anhydrous *N,N*-dimethylformamide (2.32 g, 30.0 mmol) in an ice bath was slowly treated with POCl_3_ (3.38 g, 30.0 mmol). Then, the reaction mixture was stirred for 30 min at this temperature. Afterward, pyrrole (1.25 g, 18.4 mmol) was added to the reaction mixture. The mixture was stirred for 6 h and then quenched with cold water. A solution of NaOH (5 g) in deionized H_2_O (15 ml) was added, and the resulting solution was stirred for 1 h. The reaction mixture was extracted with EtOAc (4 × 25 ml). The extracts were washed with brine (6 × 15 ml), dried over MgSO_4_, and evaporated. Separation by column chromatography eluted with hexane:ethyl acetate (3:1) gave successively compounds 7a and 7b. The characteristics of the products match the literature.^2^

1. **^1^H- and ^13^C-NMR spectrum copies of compounds**


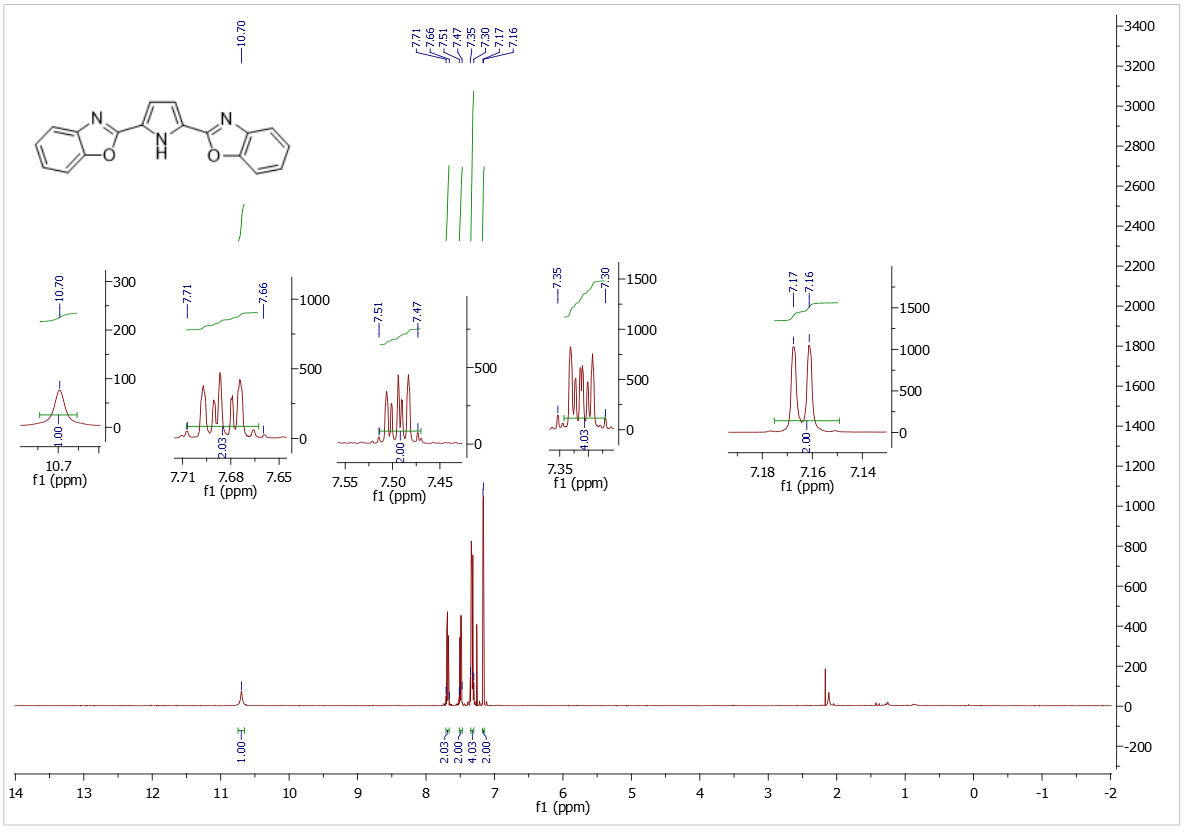


**Figure S1.** ^1^H-NMR Spectrum of compound **B1**


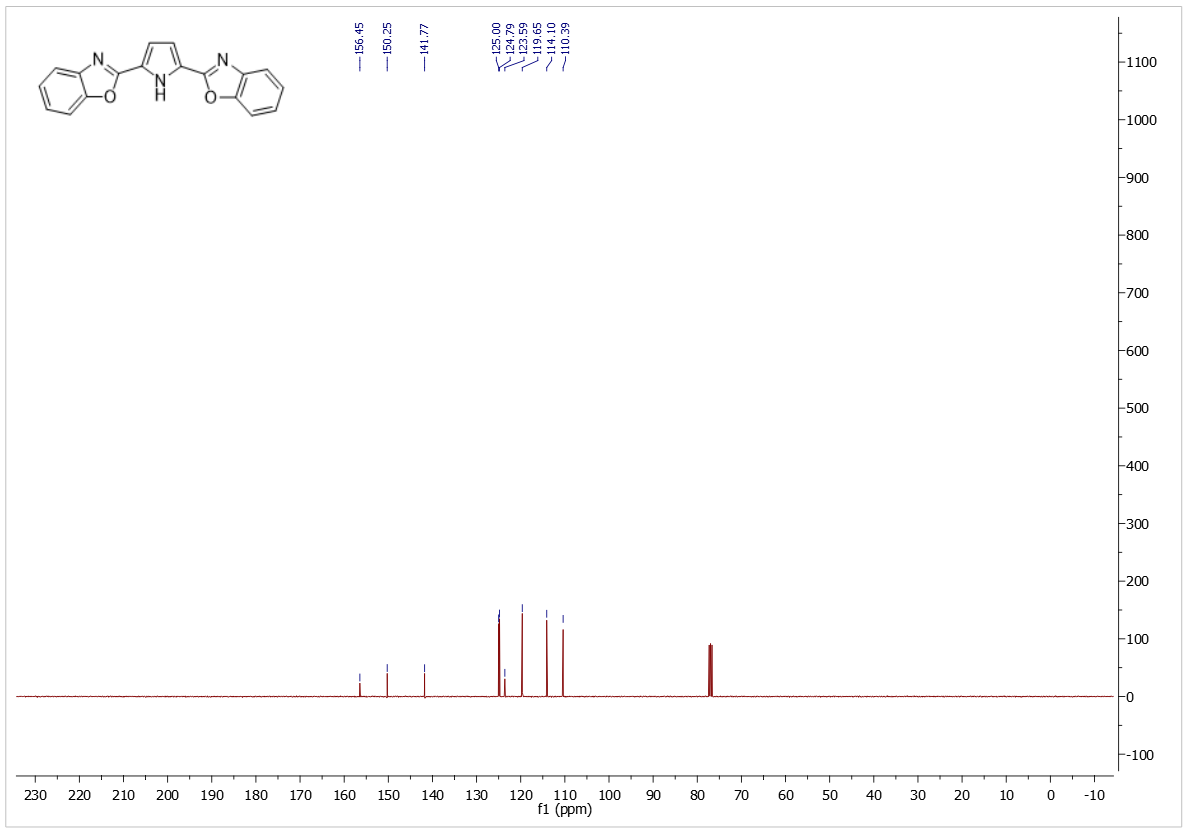


**Figure S2.** ^13^C-NMR Spectrum of compound **B1**


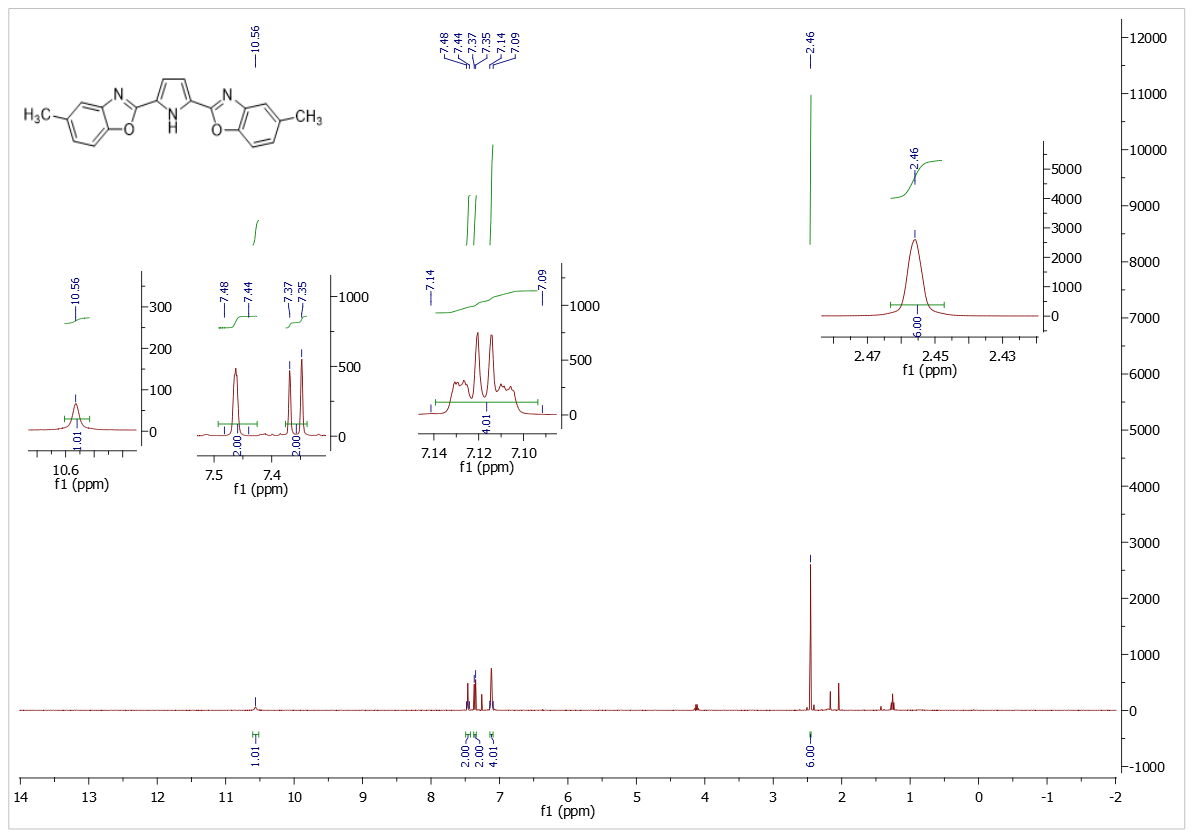


**Figure S3.** ^1^H-NMR Spectrum of compound **B2**


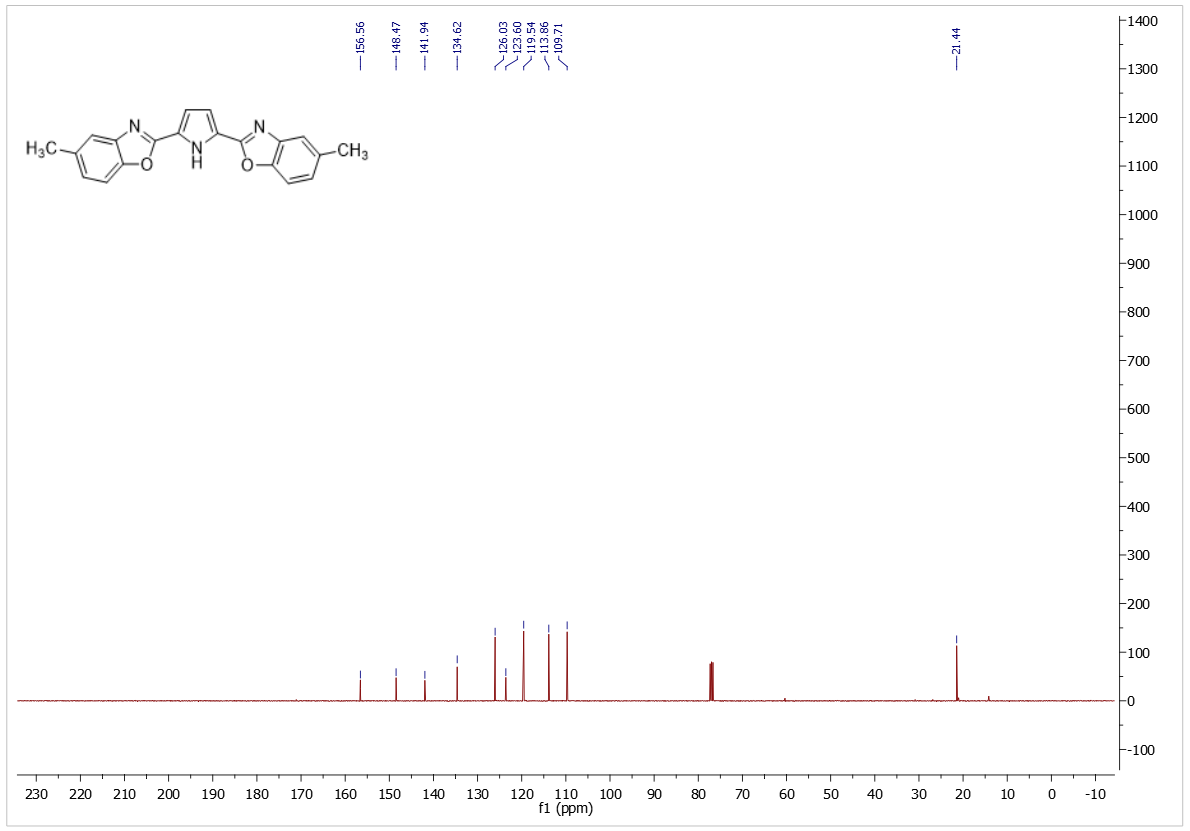


**Figure S4.** ^13^C-NMR Spectrum of compound **B2**


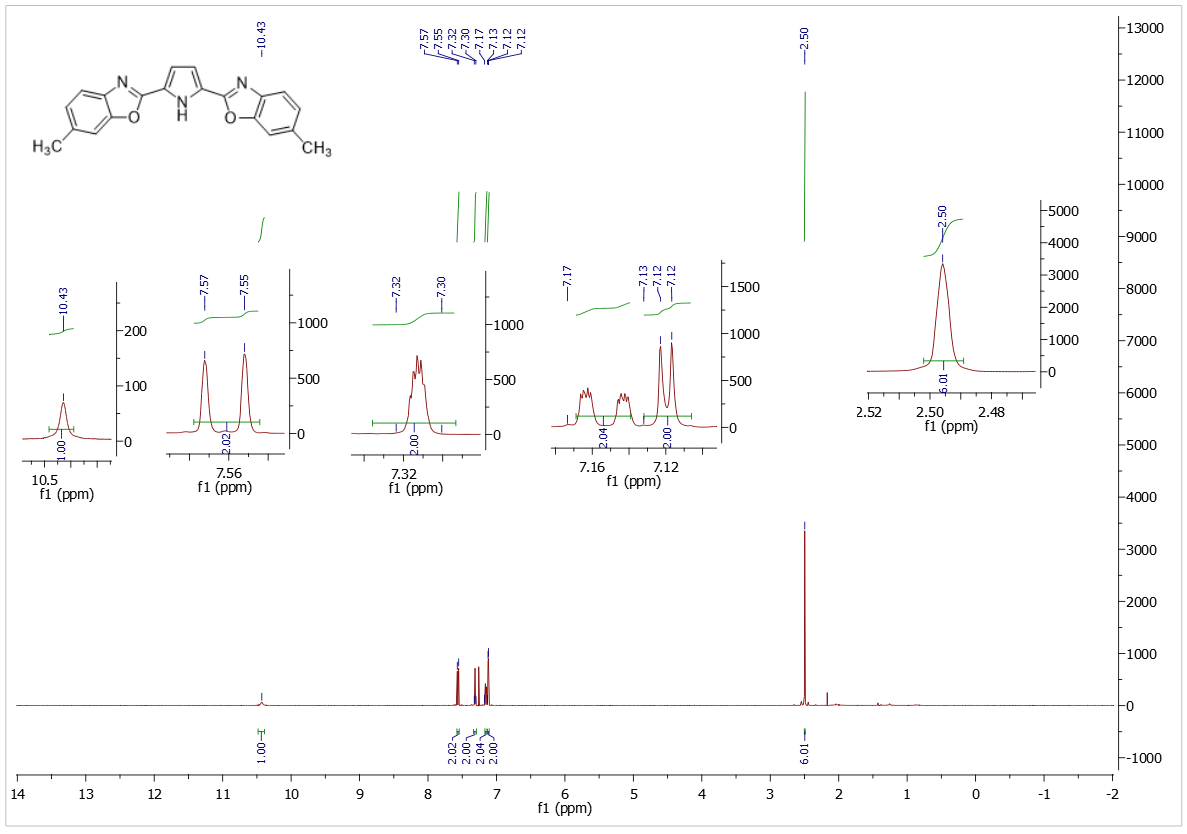


**Figure S5.** ^1^H-NMR Spectrum of compound **B3**


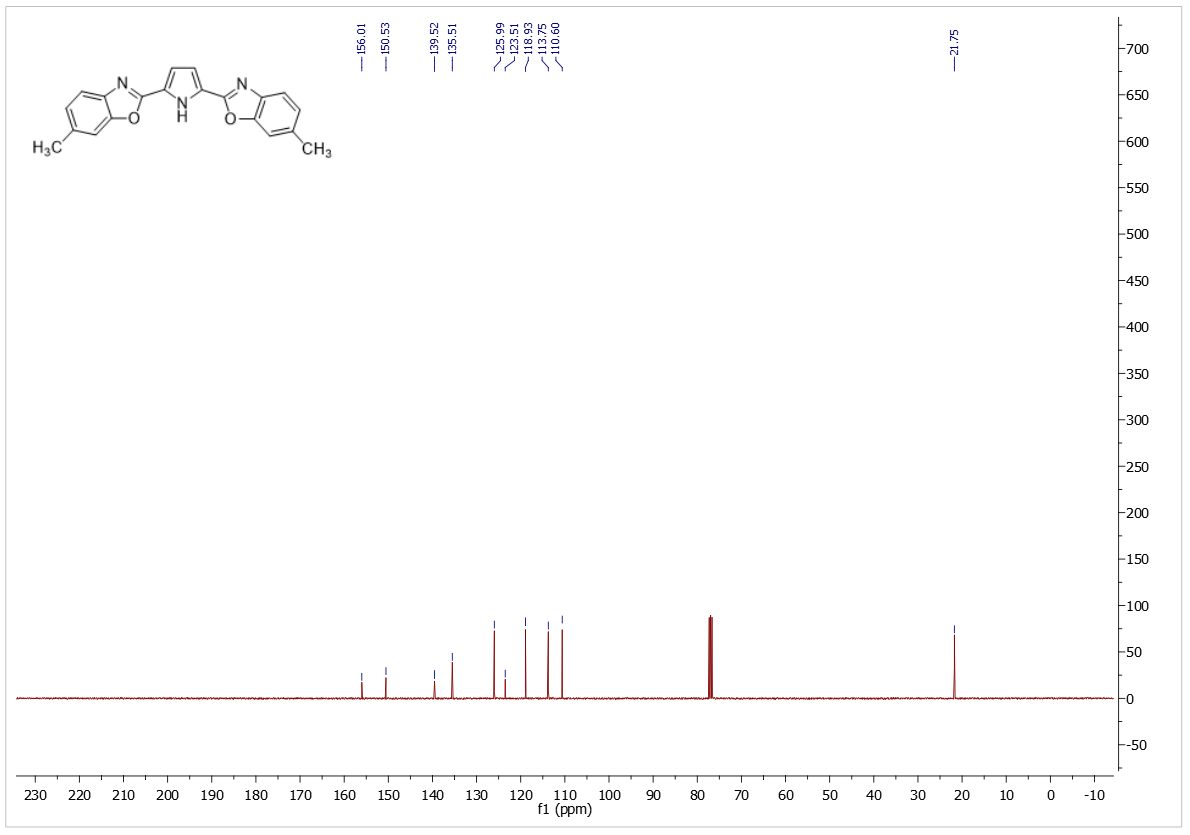


**Figure S6.** ^13^C-NMR Spectrum of compound **B3**


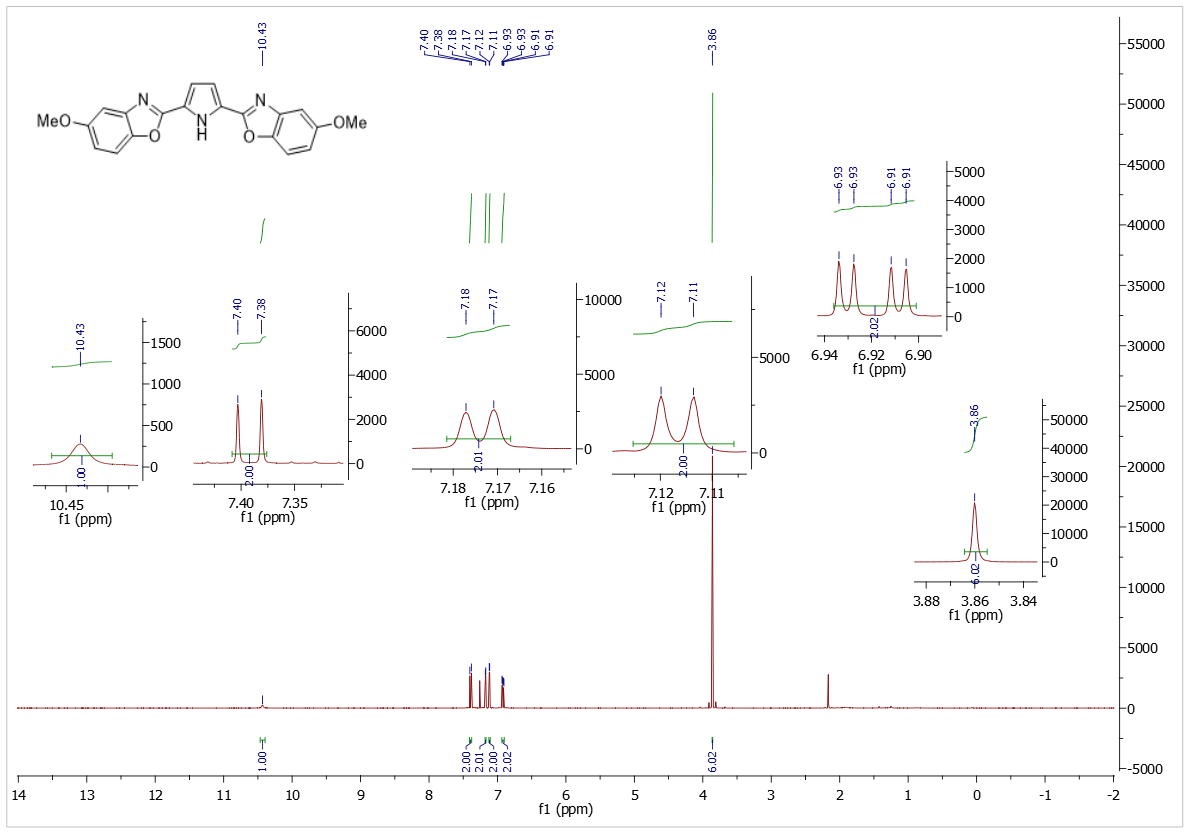


**Figure S7.** ^1^H-NMR Spectrum of compound **B4**


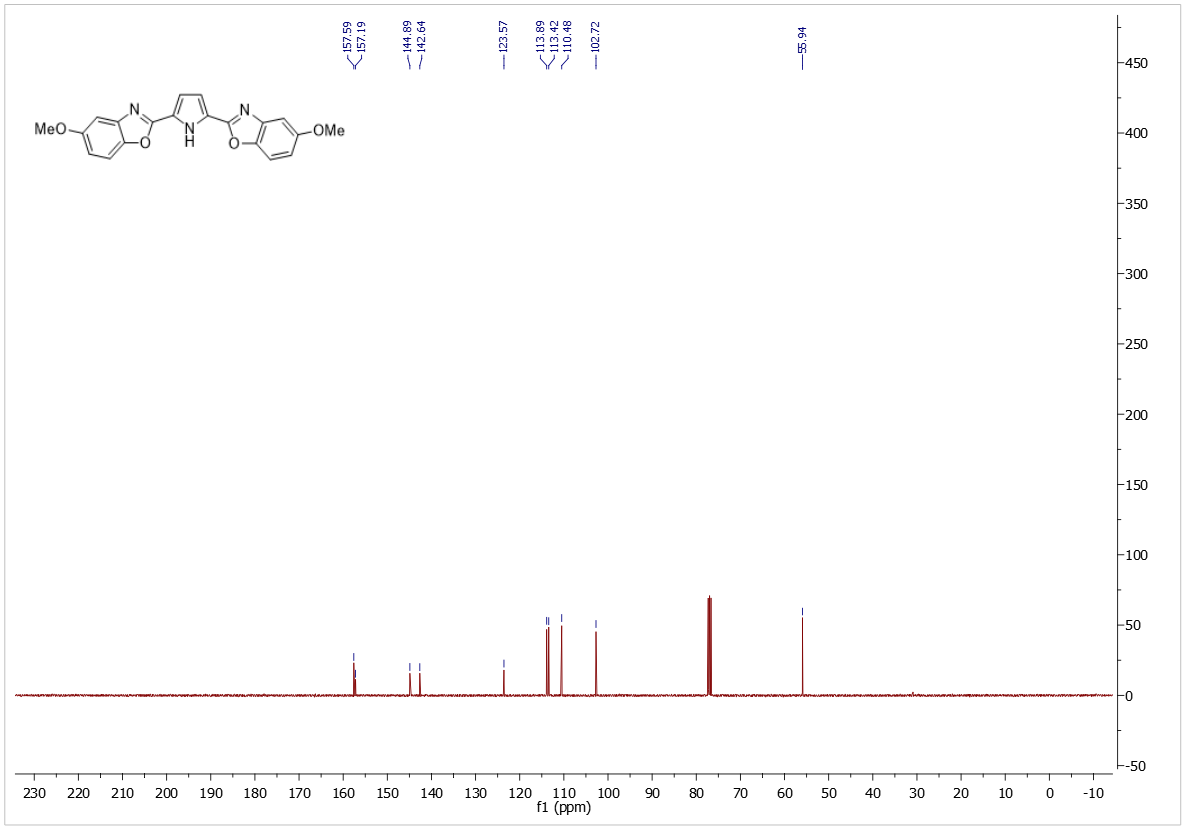


**Figure S8.** ^13^C-NMR Spectrum of compound **B4**


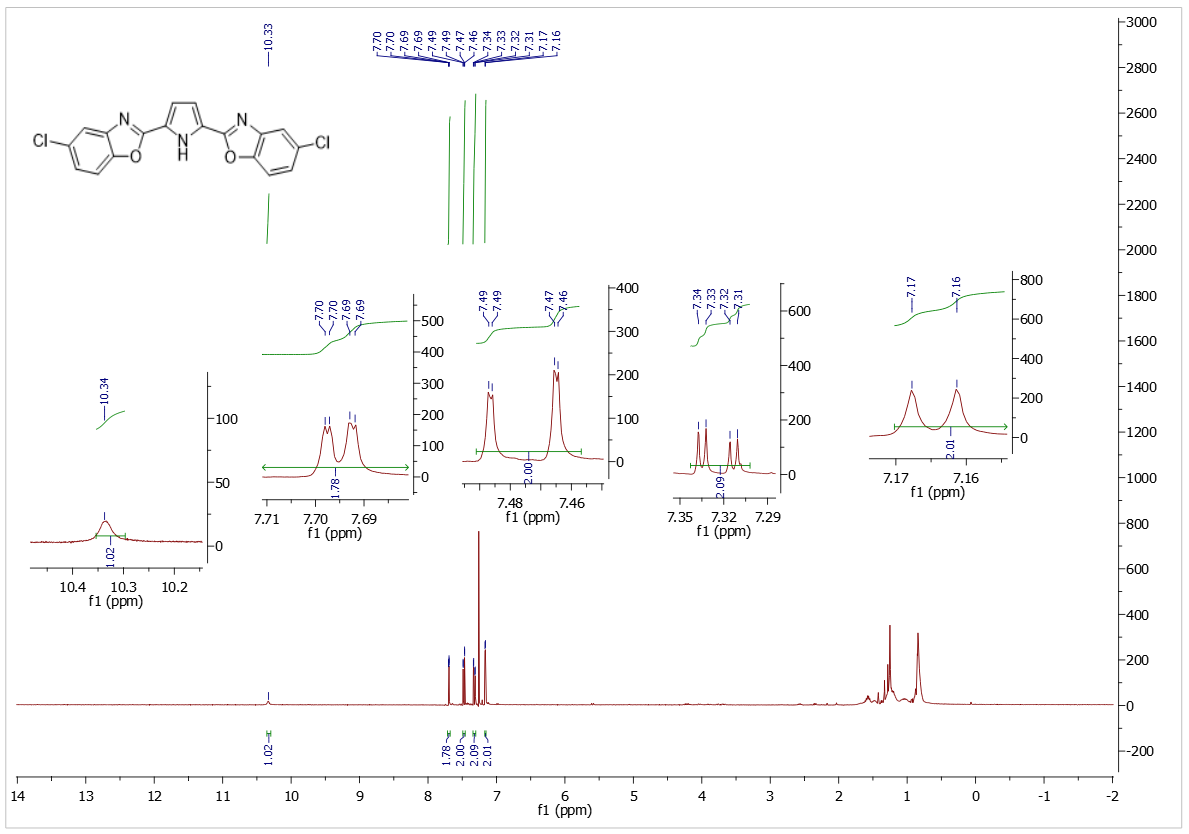


**Figure S9.** ^1^H-NMR Spectrum of compound **B5**


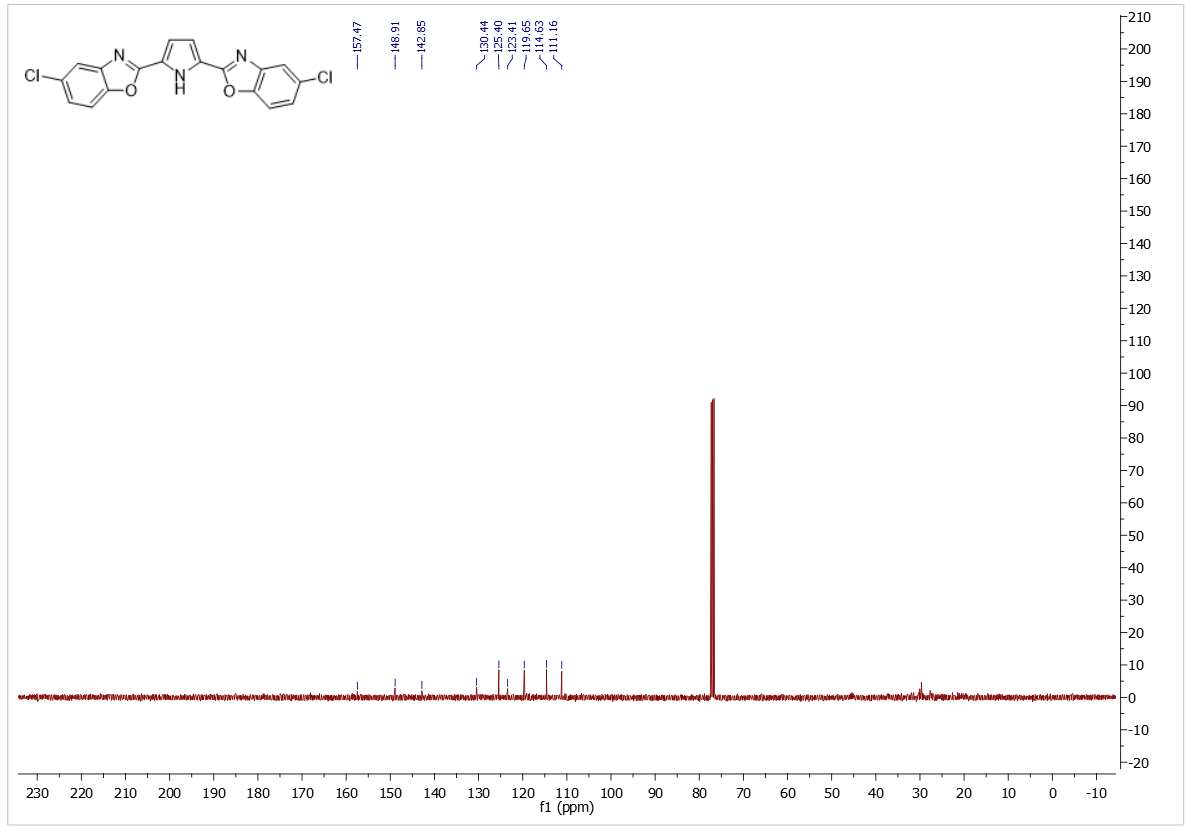


**Figure S10.** ^13^C-NMR Spectrum of compound **B5**


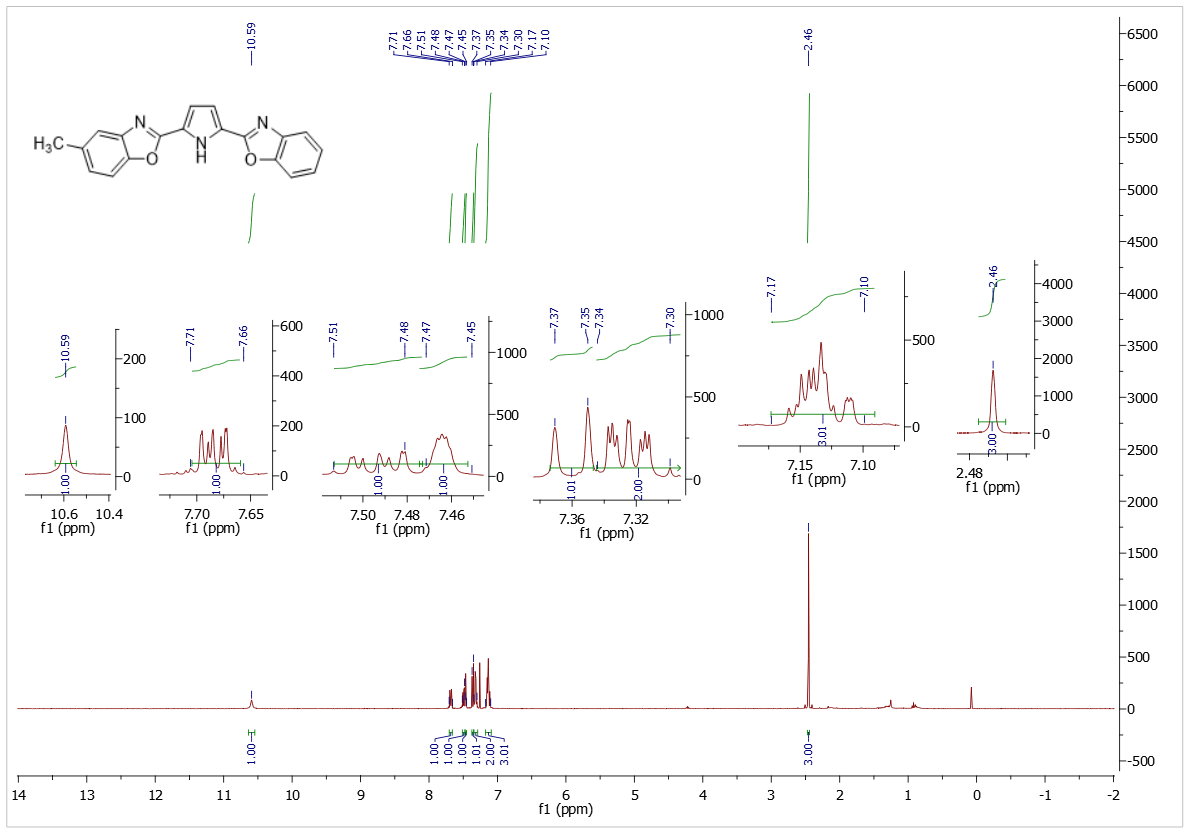


**Figure S11.** ^1^H-NMR Spectrum of compound **B6**


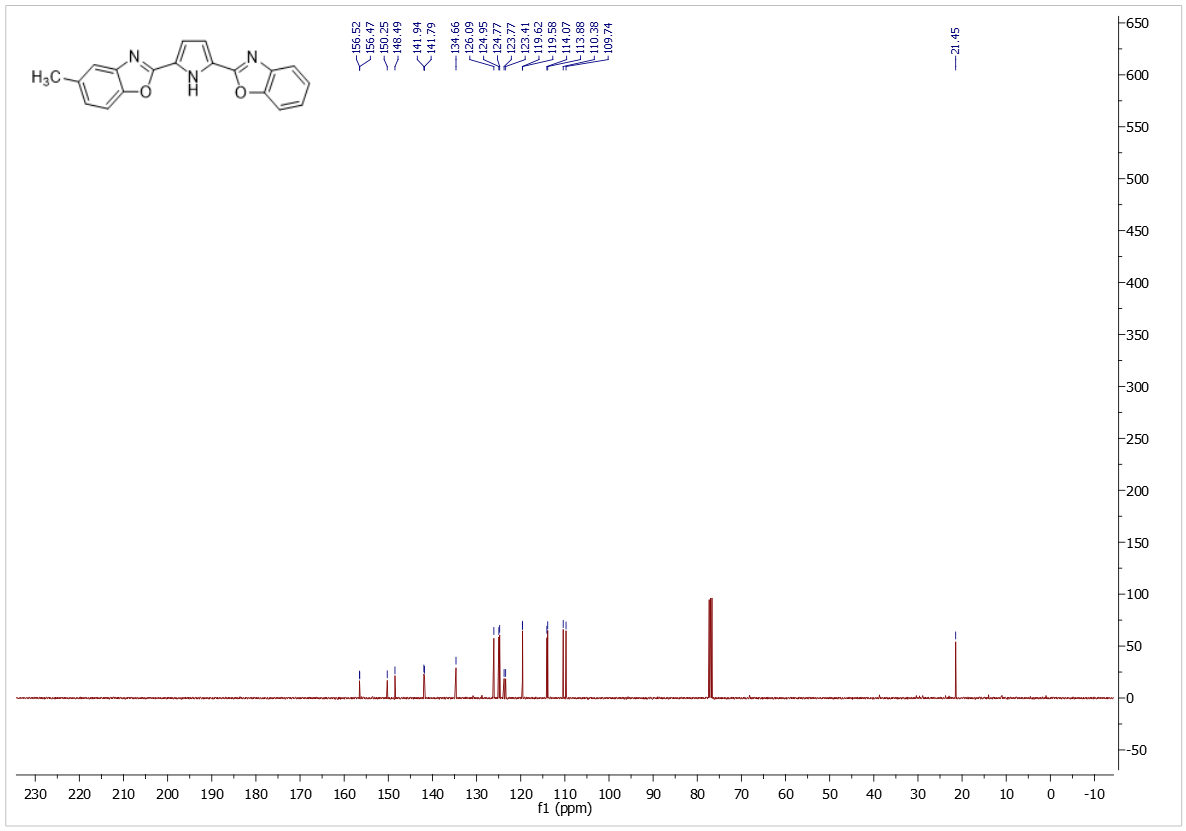


**Figure S12.** ^13^C-NMR Spectrum of compound **B6**


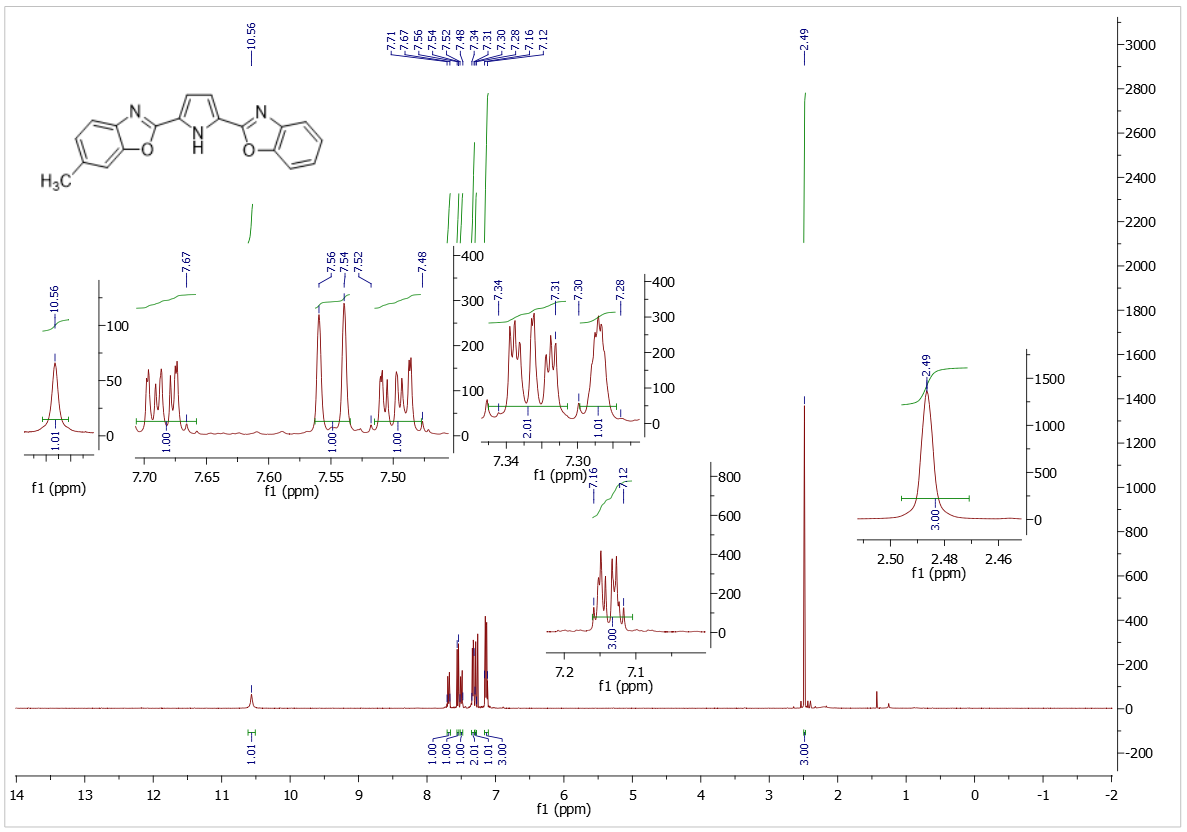


**Figure S13.** ^1^H-NMR Spectrum of compound **B7**


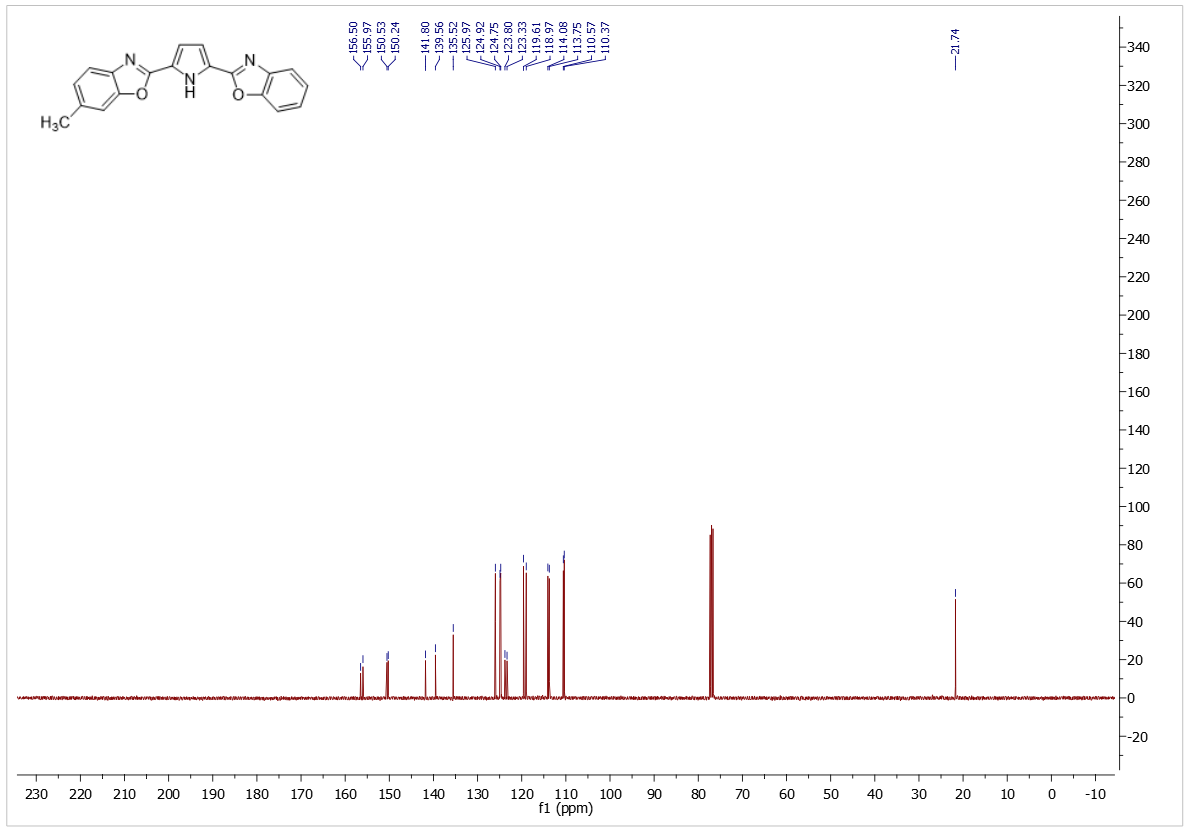


**Figure S14.** ^13^C-NMR Spectrum of compound **B7**


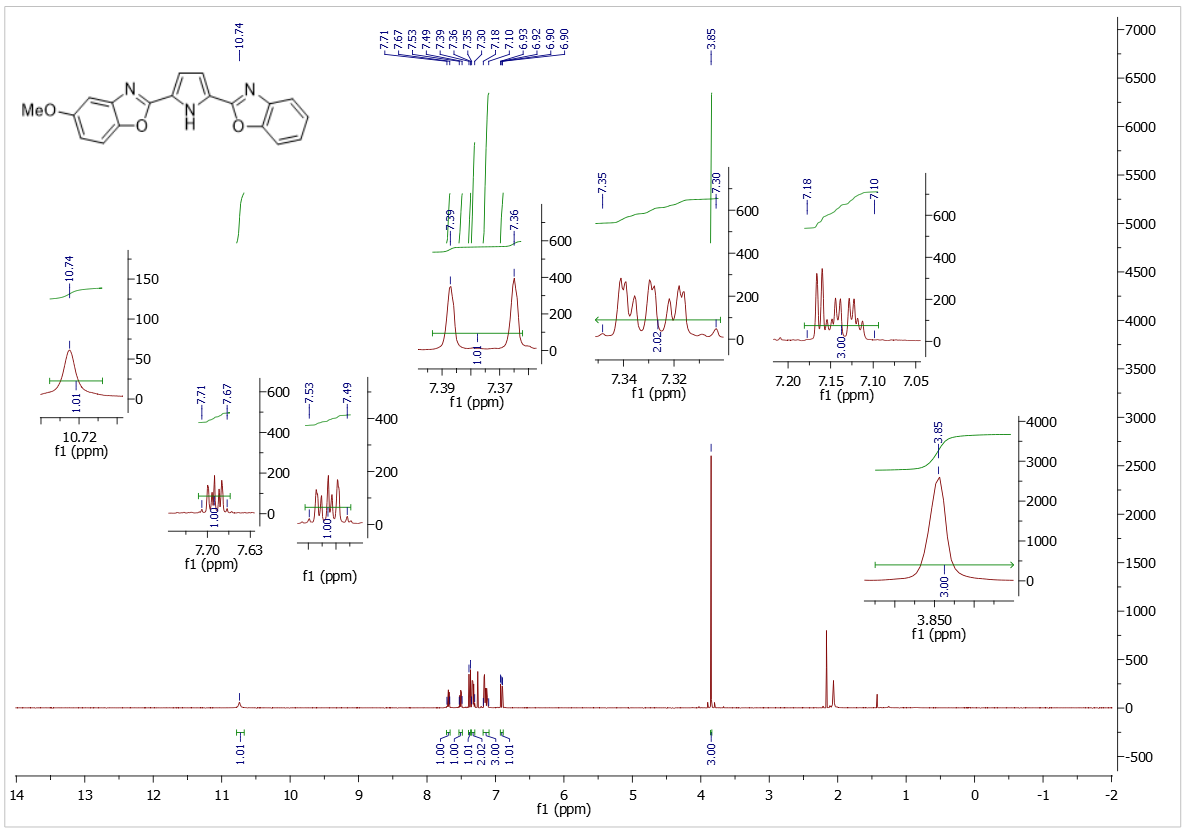


**Figure S15.** ^1^H-NMR Spectrum of compound **B8**


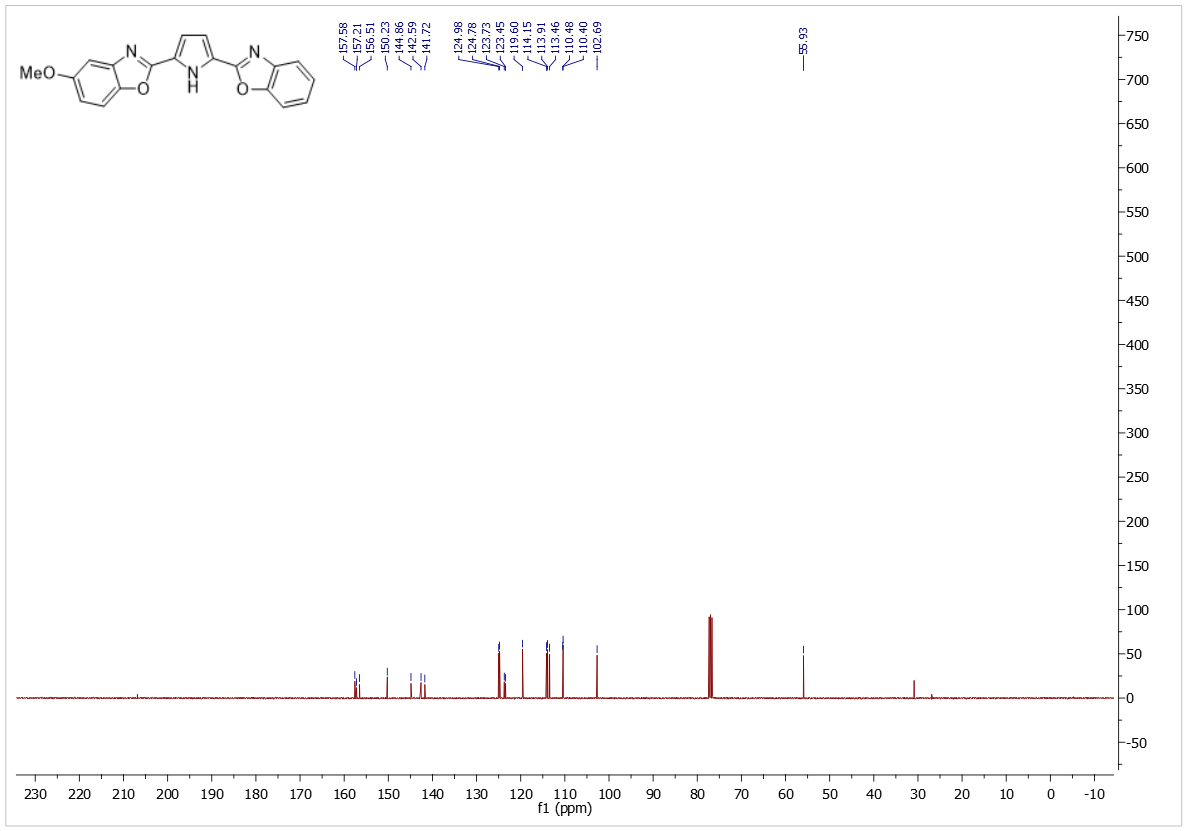


**Figure S16.** ^13^C-NMR Spectrum of compound **B8**


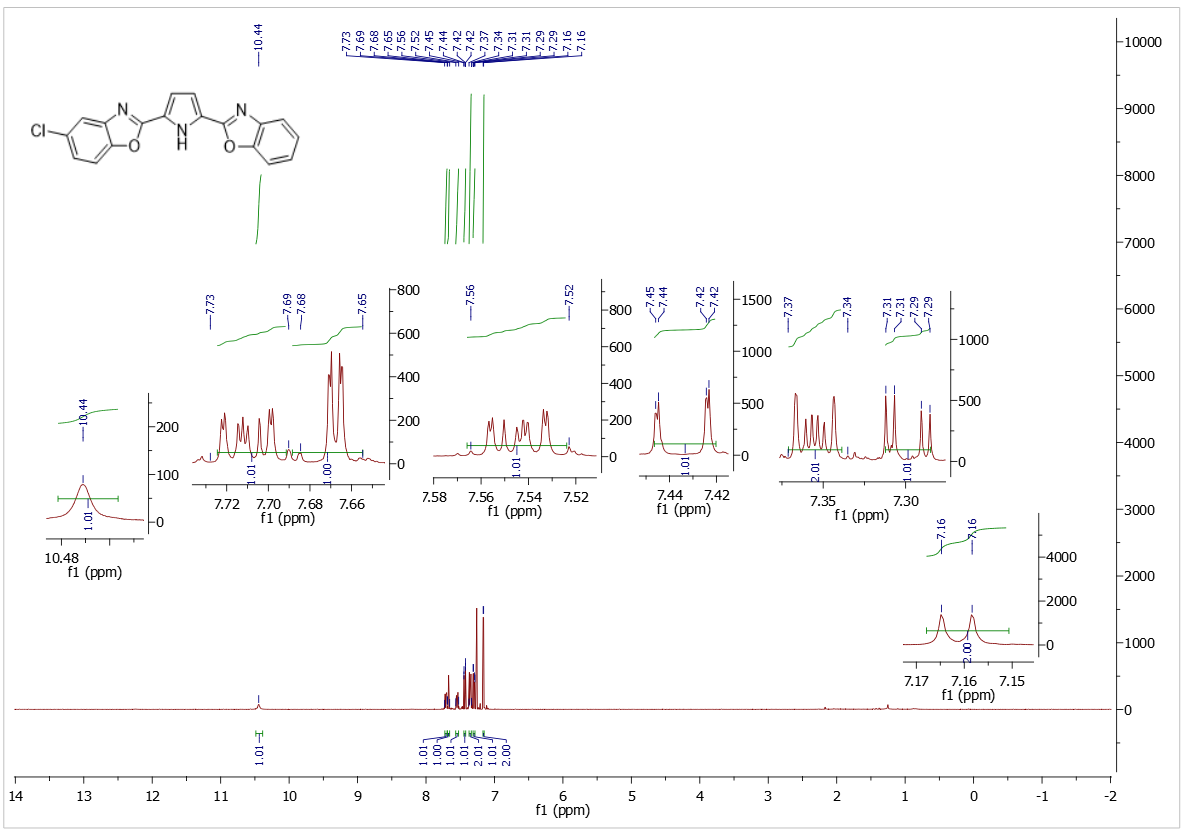


**Figure S17.** ^1^H-NMR Spectrum of compound **B9**


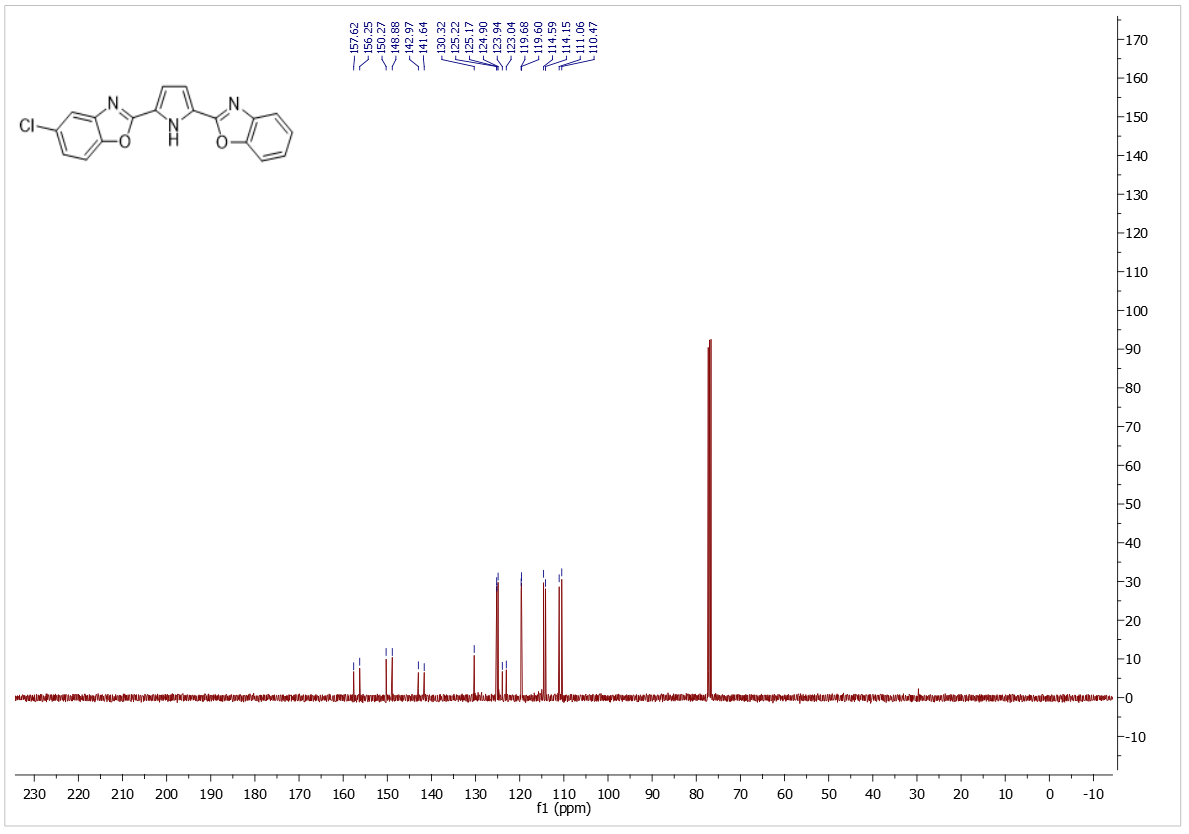


**Figure S18.** ^13^C-NMR Spectrum of compound **B9**


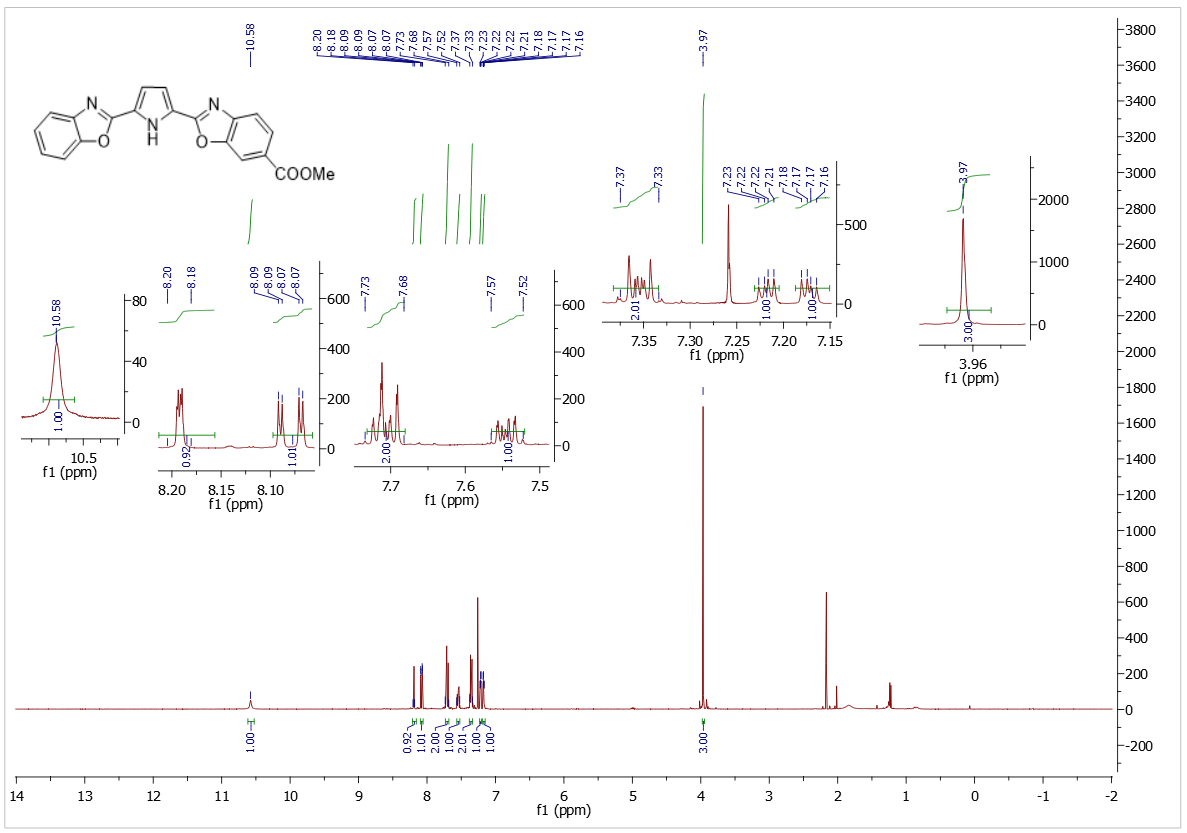


**Figure S19.** ^1^H-NMR Spectrum of compound **B10**


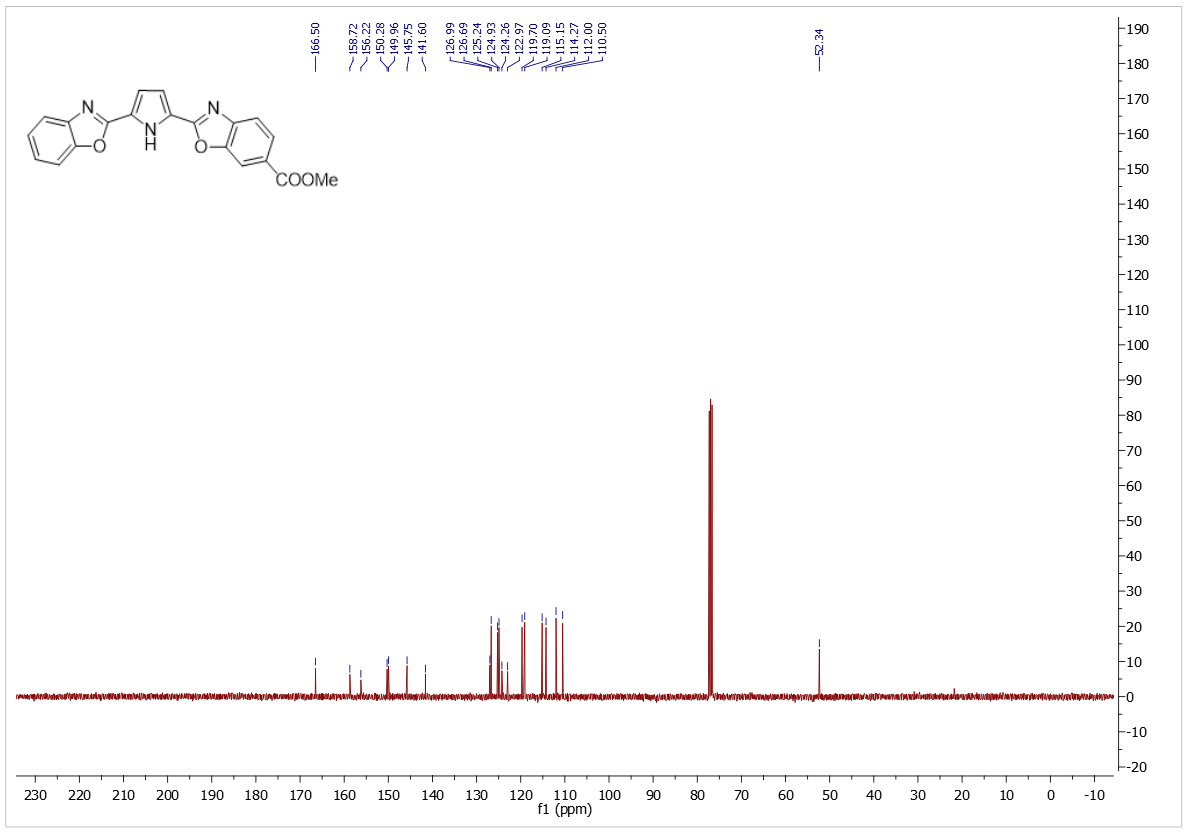


**Figure S20.** ^13^C-NMR Spectrum of compound **B10**


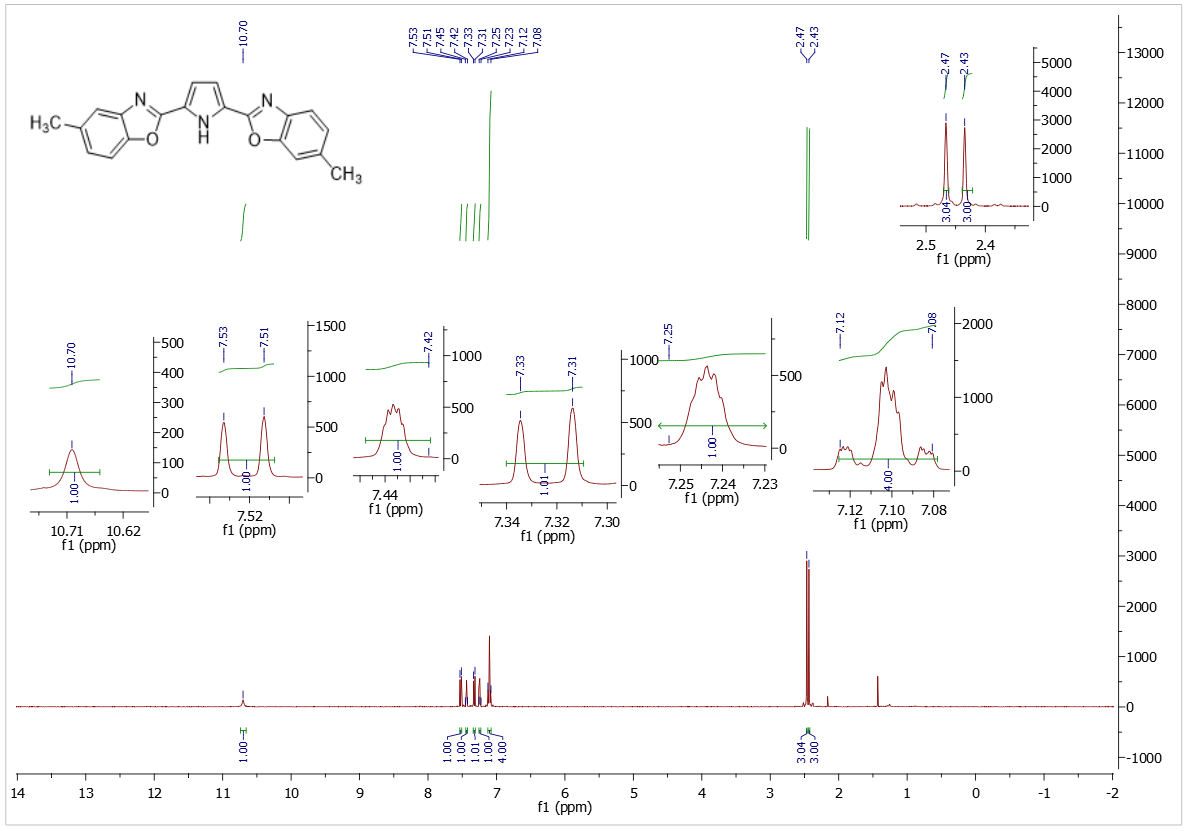


**Figure S21.** ^1^H-NMR Spectrum of compound **B11**


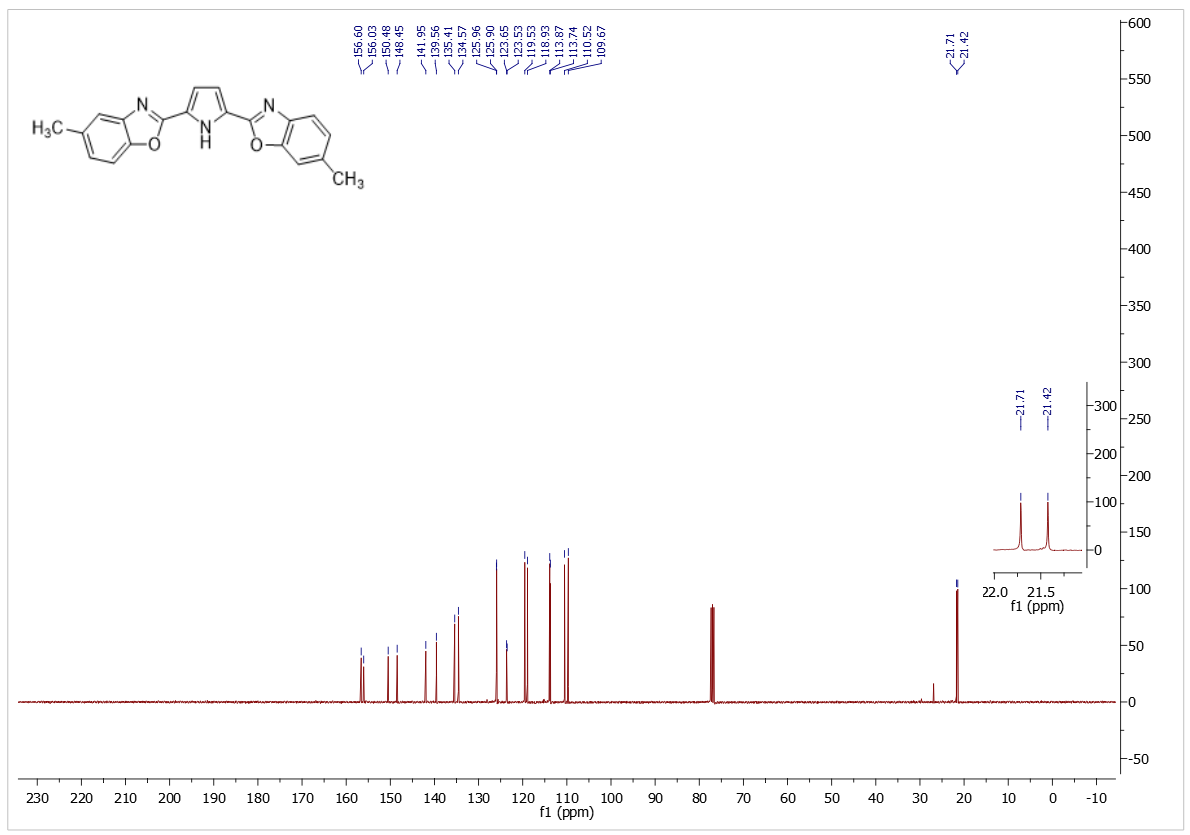


**Figure S22.** ^13^C-NMR Spectrum of compound **B11**


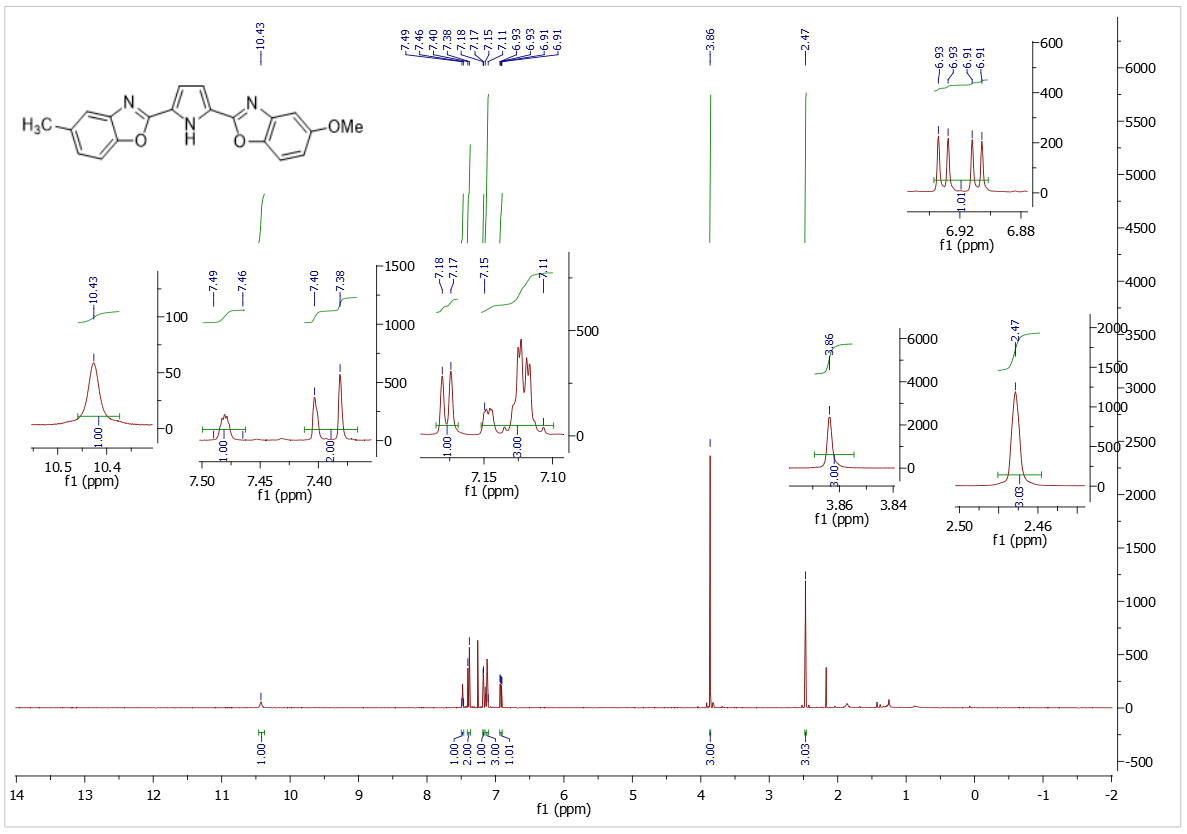


**Figure S23.** ^1^H-NMR Spectrum of compound **B12**


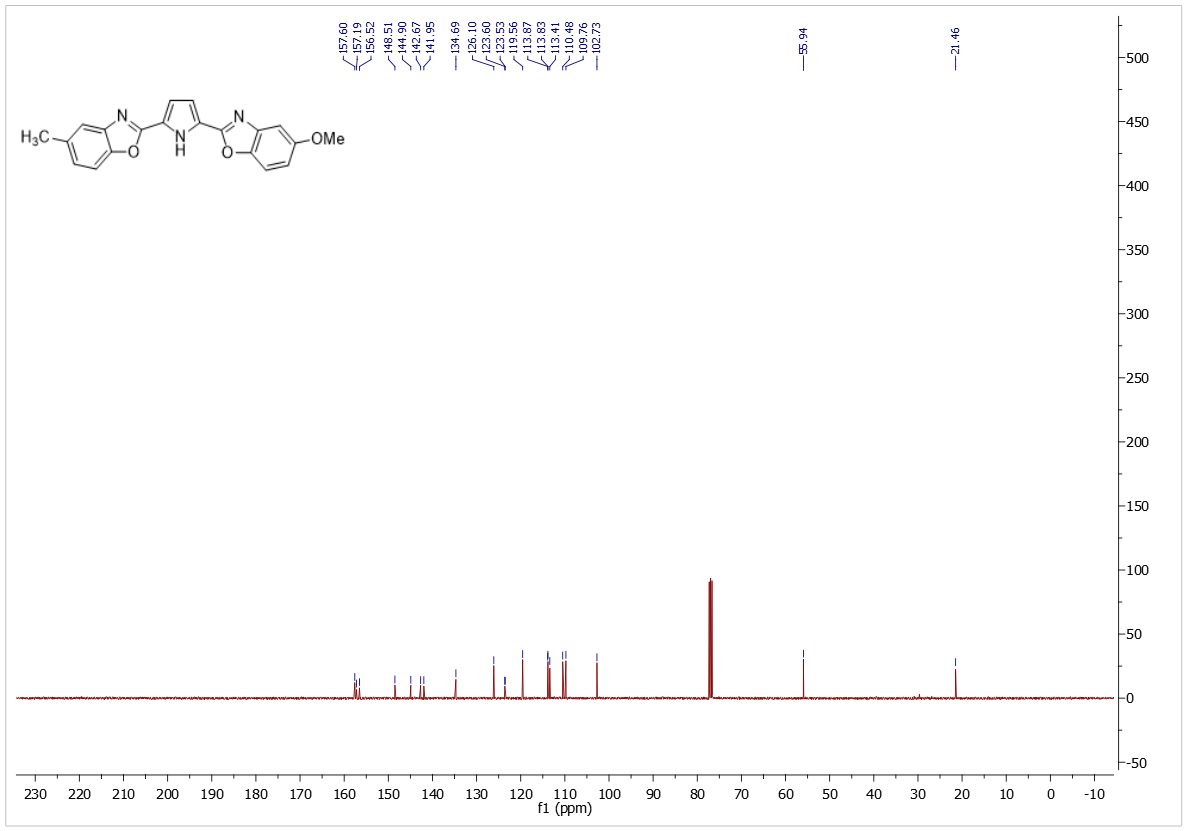


**Figure S24.** ^13^C-NMR Spectrum of compound **B12**


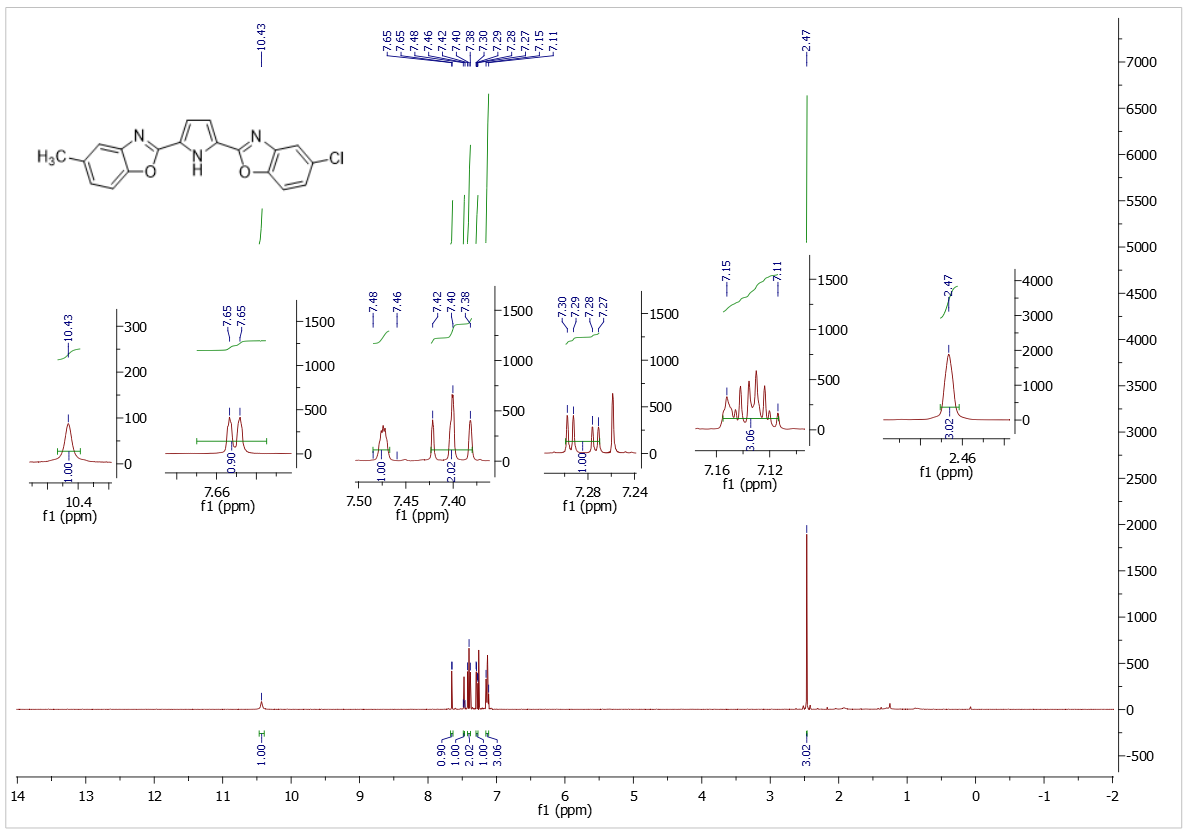


**Figure S25.** ^1^H-NMR Spectrum of compound **B13**


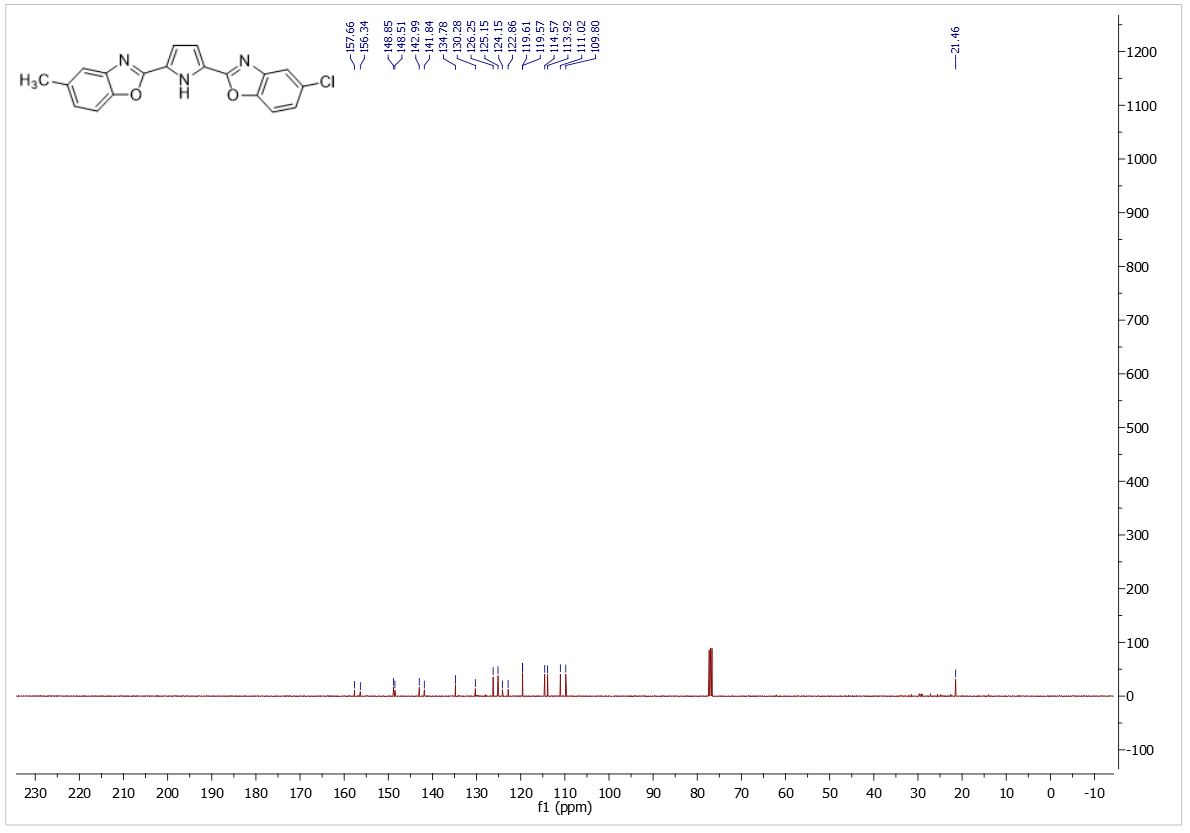


**Figure S26.** ^13^C-NMR Spectrum of compound **B13**


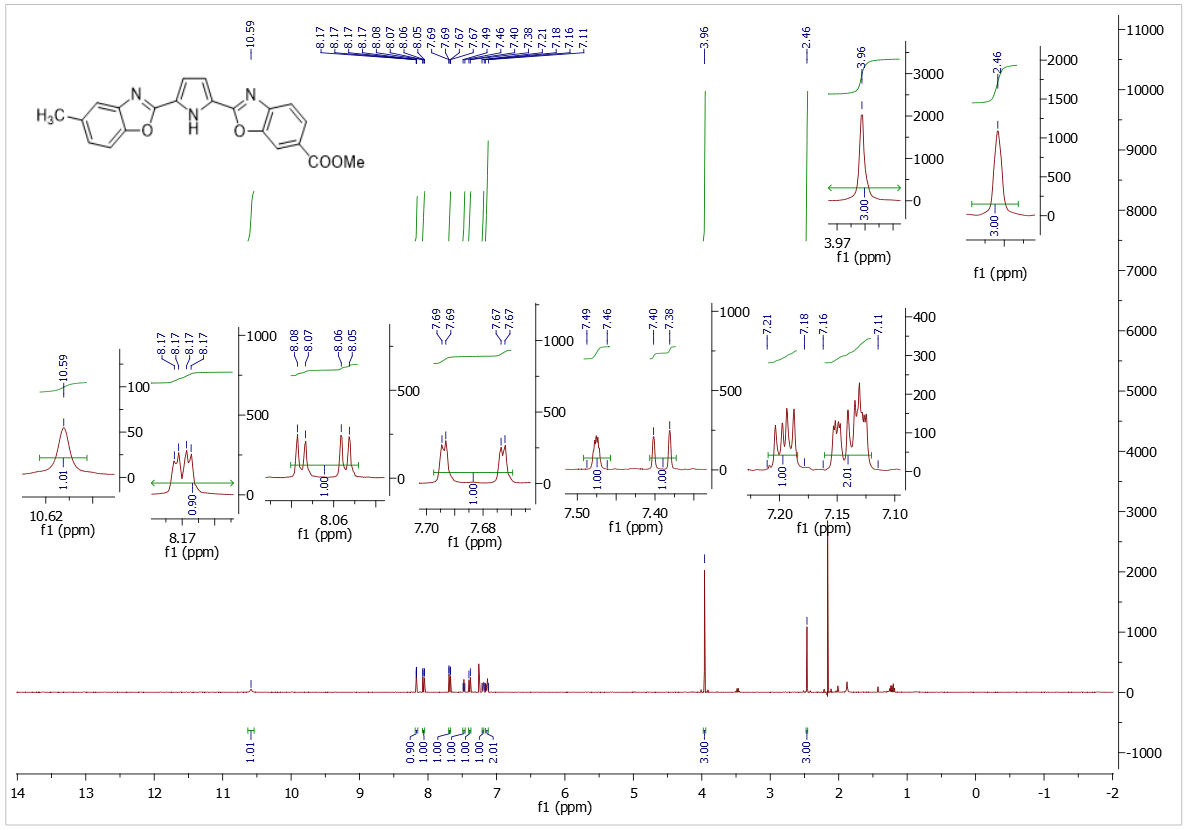


**Figure S27.** ^1^H-NMR Spectrum of compound **B14**


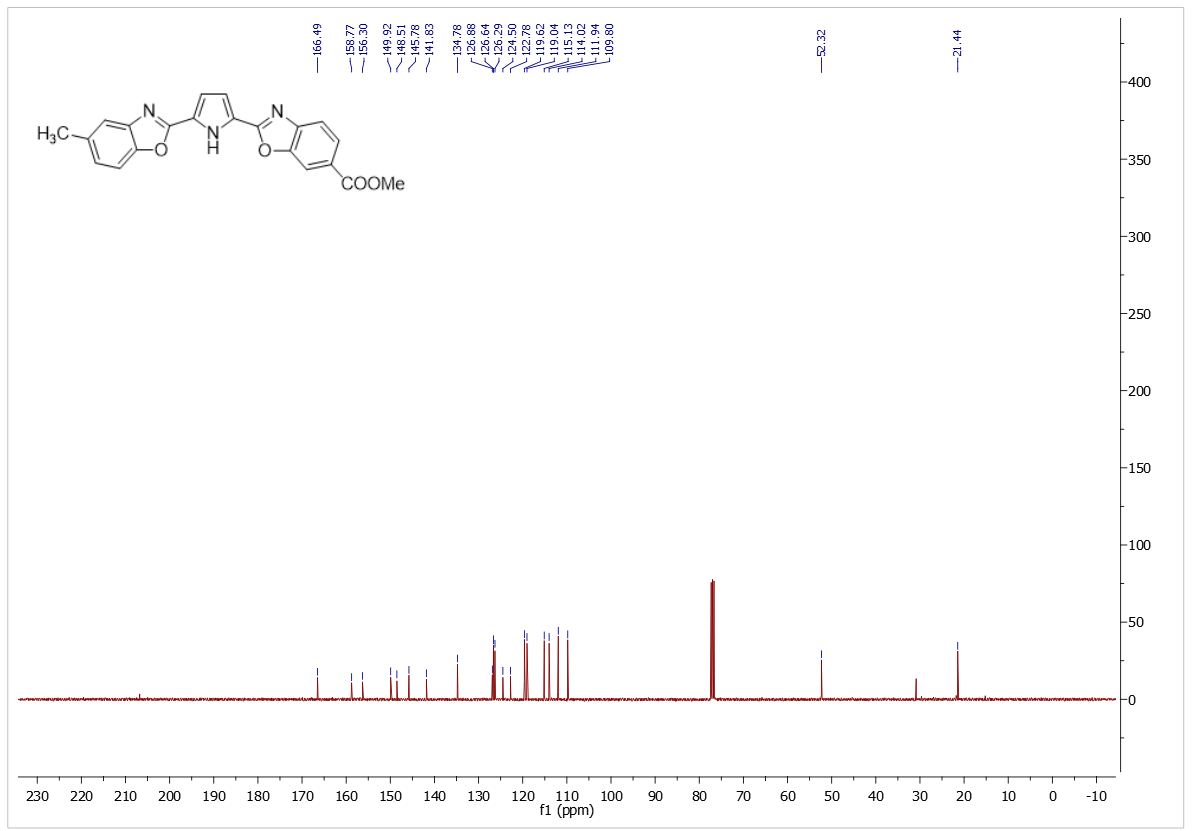


**Figure S28.** ^13^C-NMR Spectrum of compound **B14**


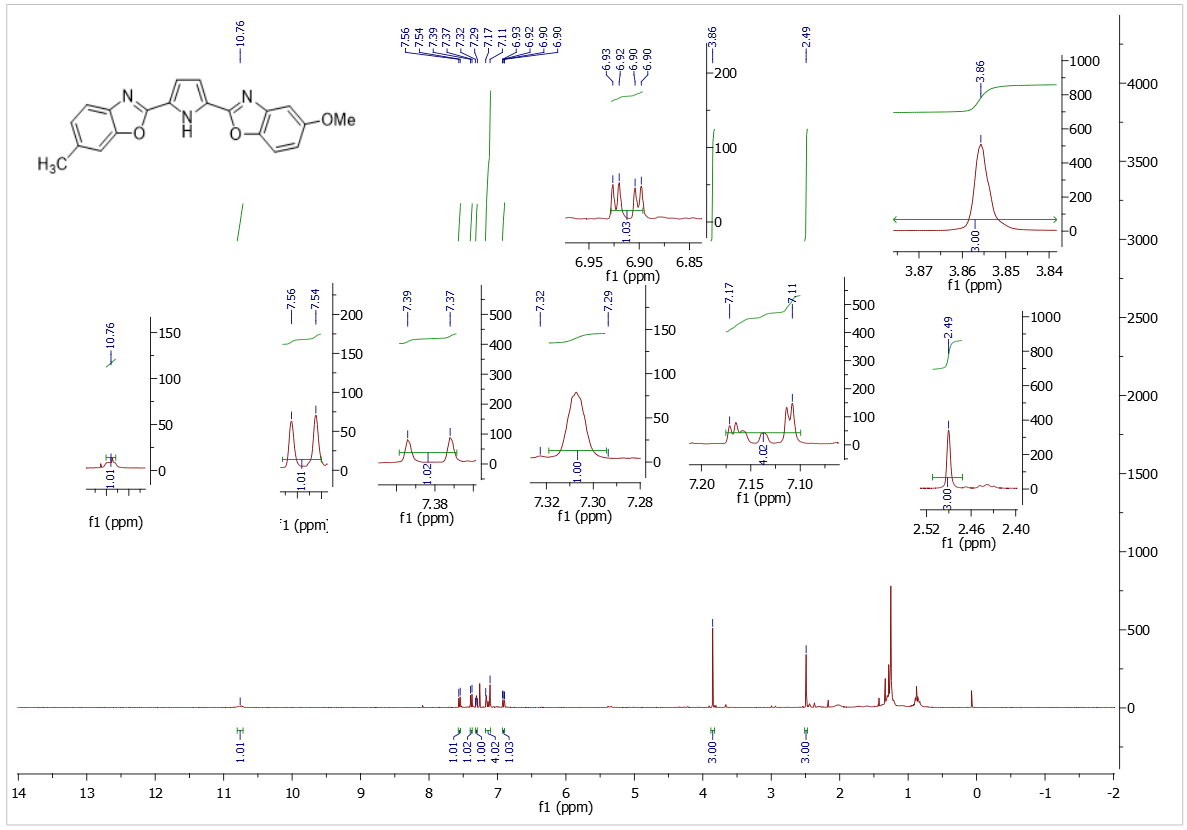


**Figure S29.** ^1^H-NMR Spectrum of compound **B15**


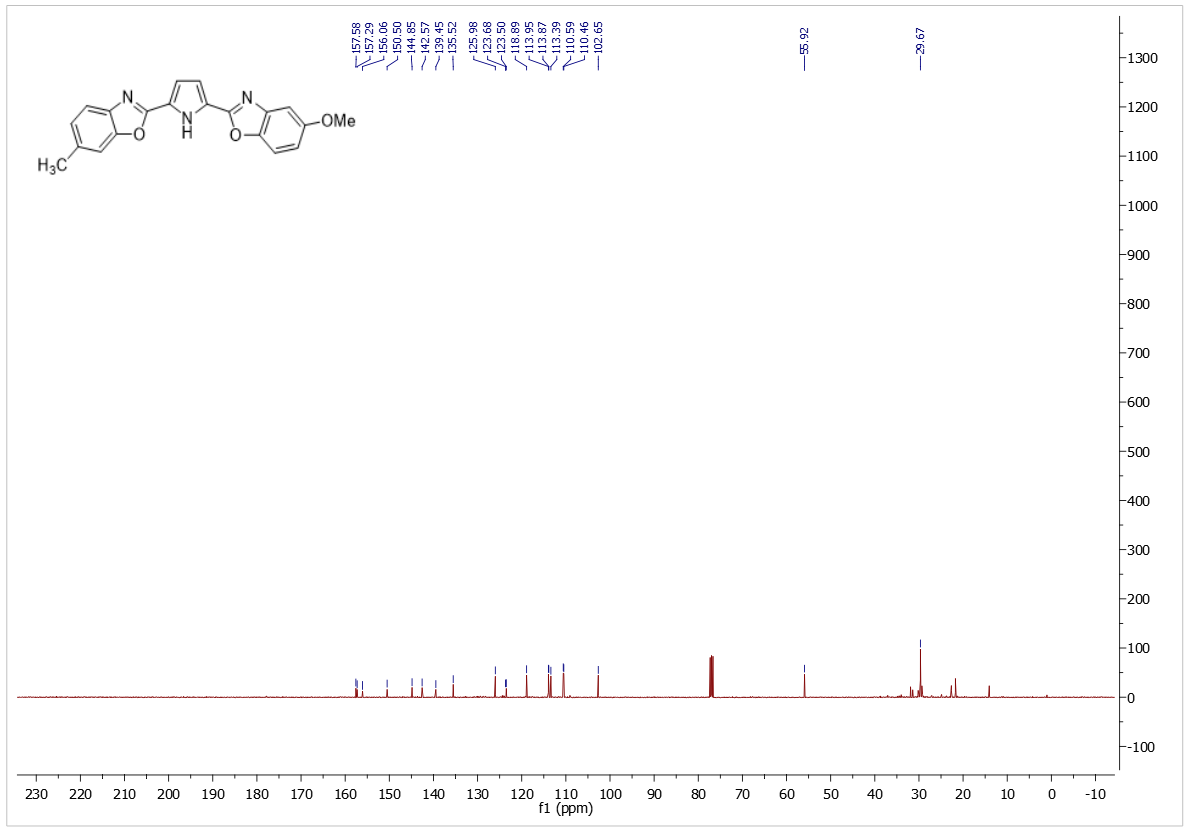


**Figure S30.** ^13^C-NMR Spectrum of compound **B15**


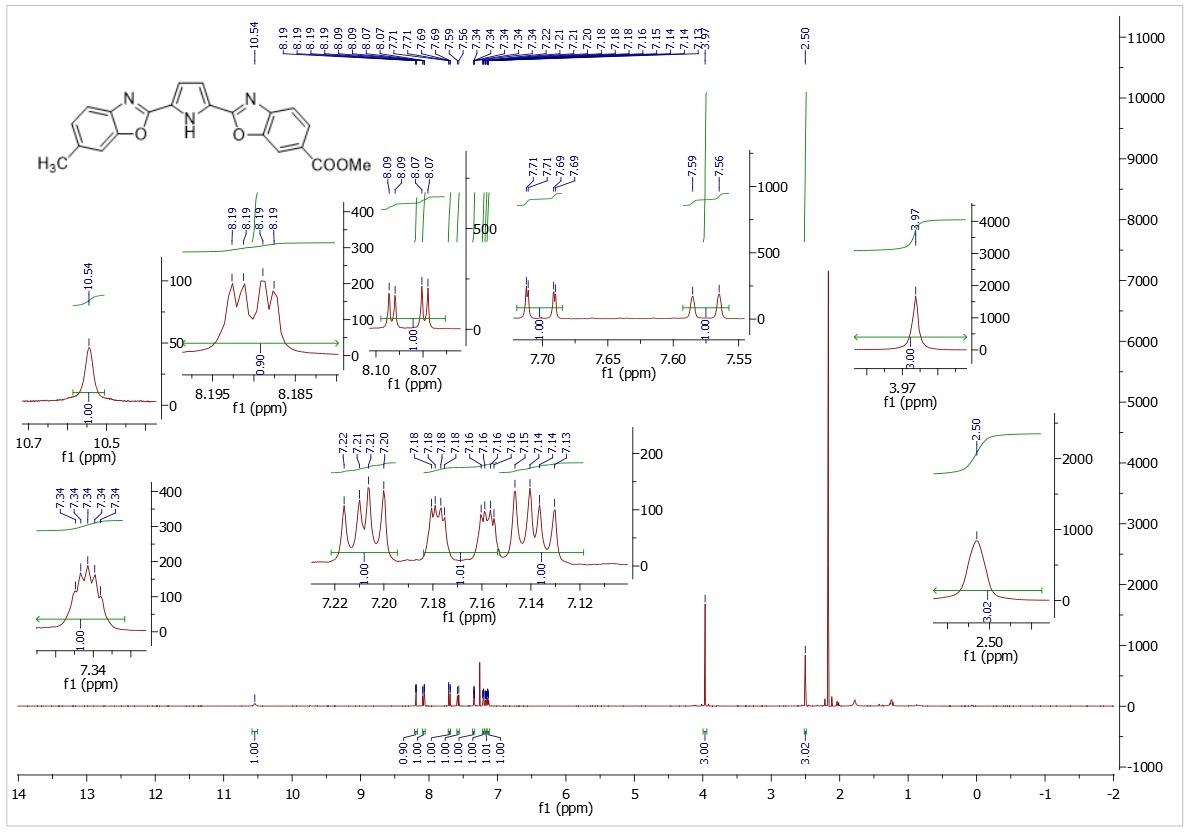


**Figure S31.** ^1^H-NMR Spectrum of compound **B16**


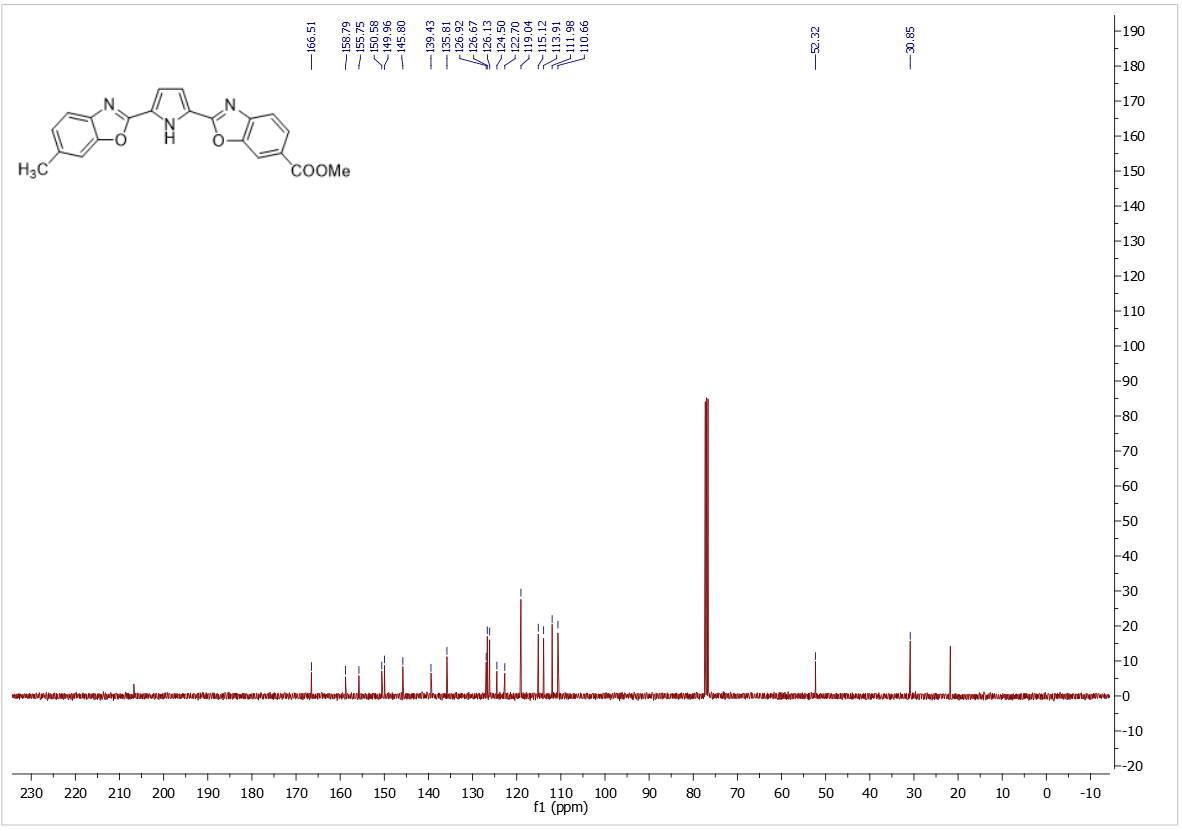


**Figure S32.** ^13^C-NMR Spectrum of compound **B16**


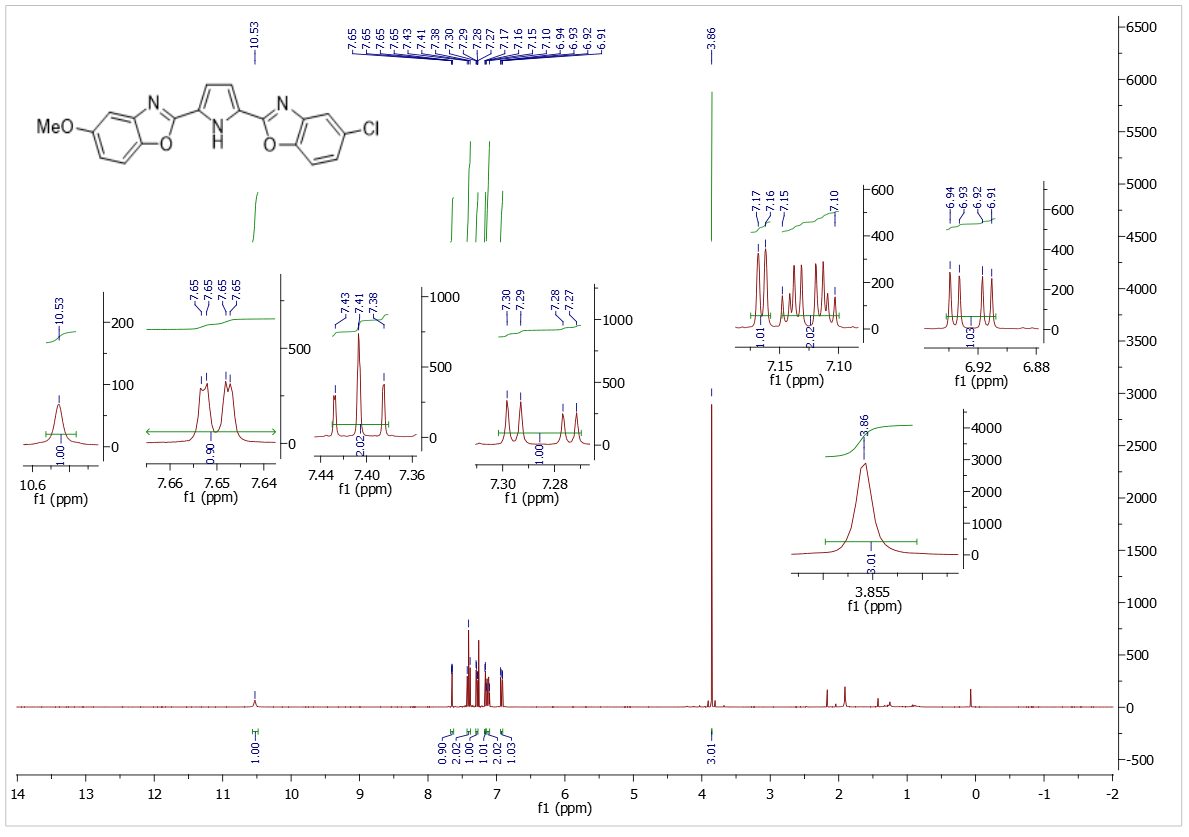


**Figure S33.** ^1^H-NMR Spectrum of compound **B17**


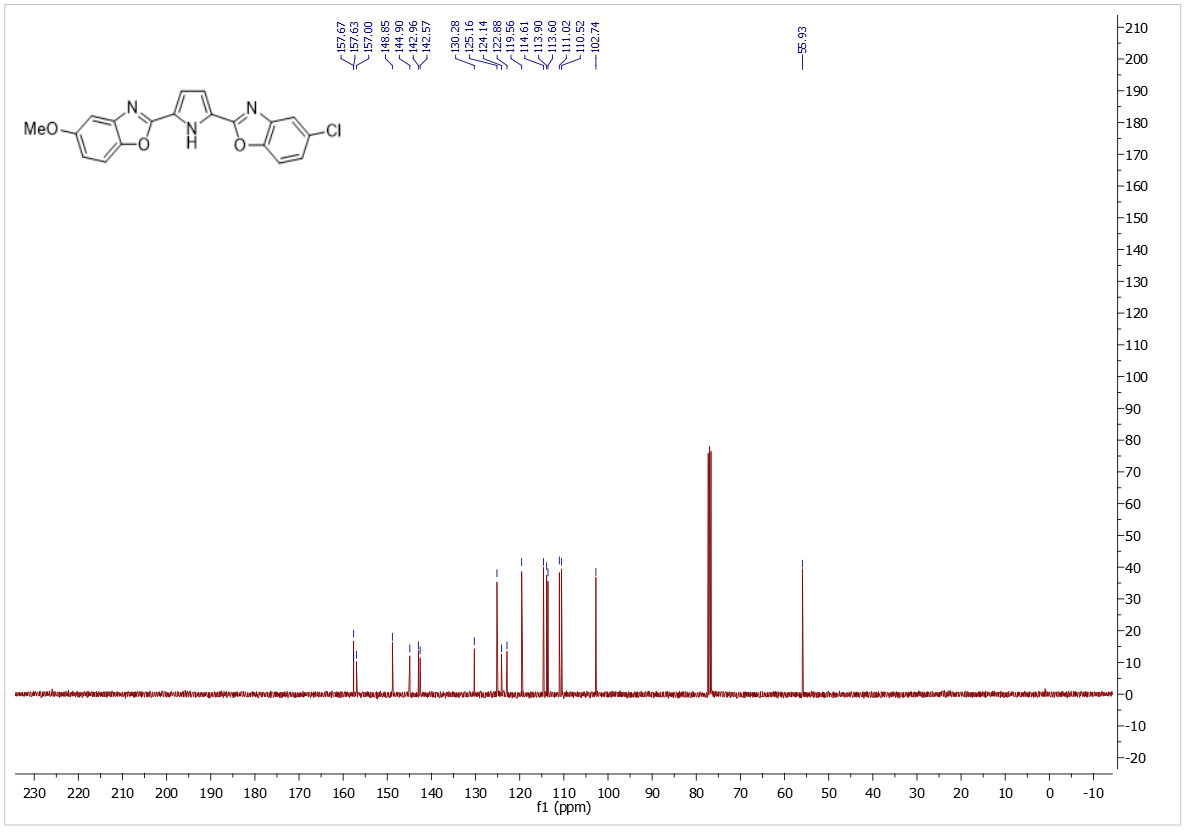


**Figure S34.** ^13^C-NMR Spectrum of compound **B17**


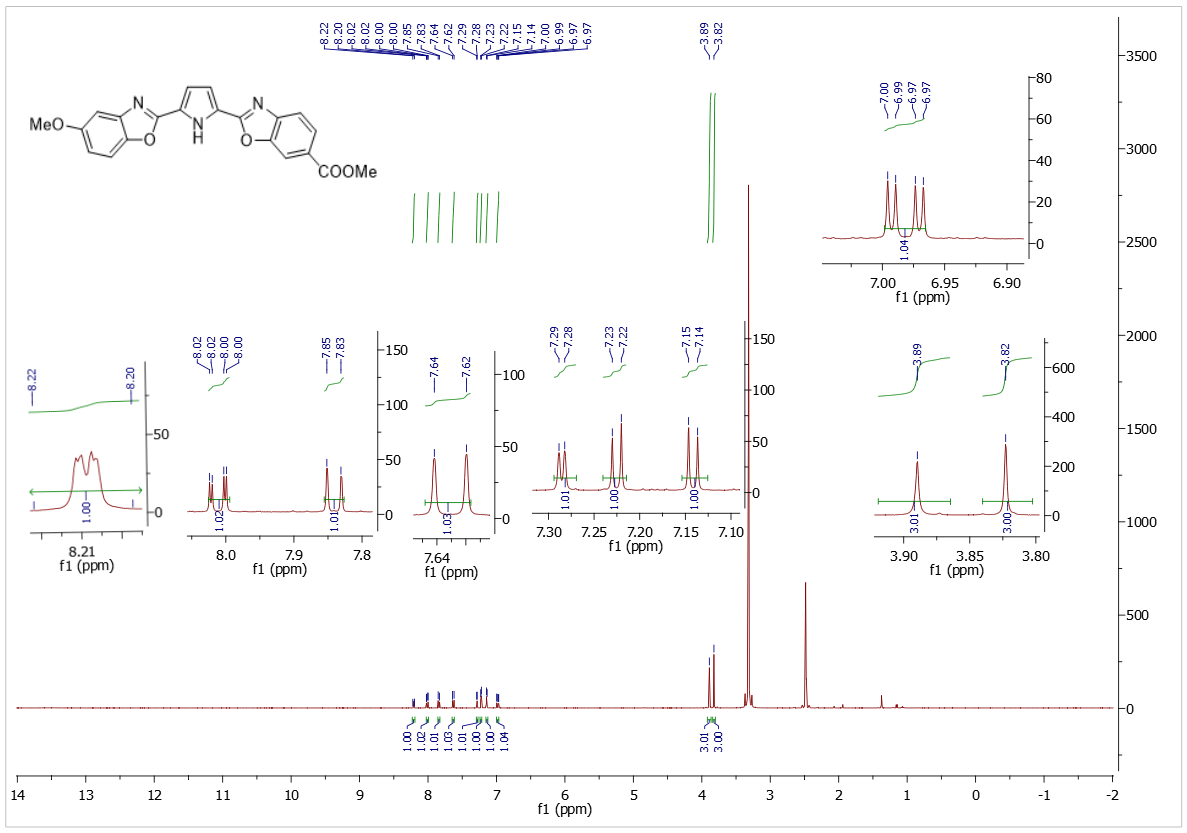


**Figure S35.** ^1^H-NMR Spectrum of compound **B18**


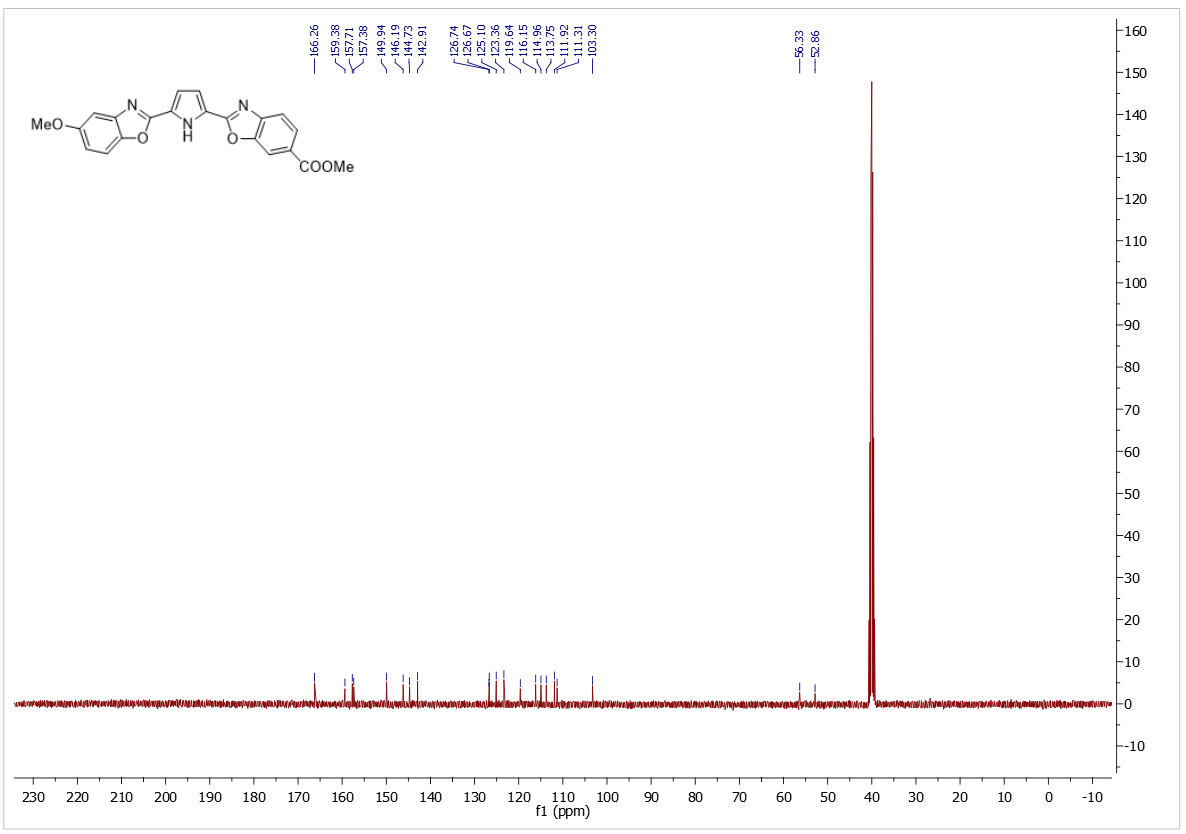


**Figure S36.** ^13^C-NMR Spectrum of compound **B18**


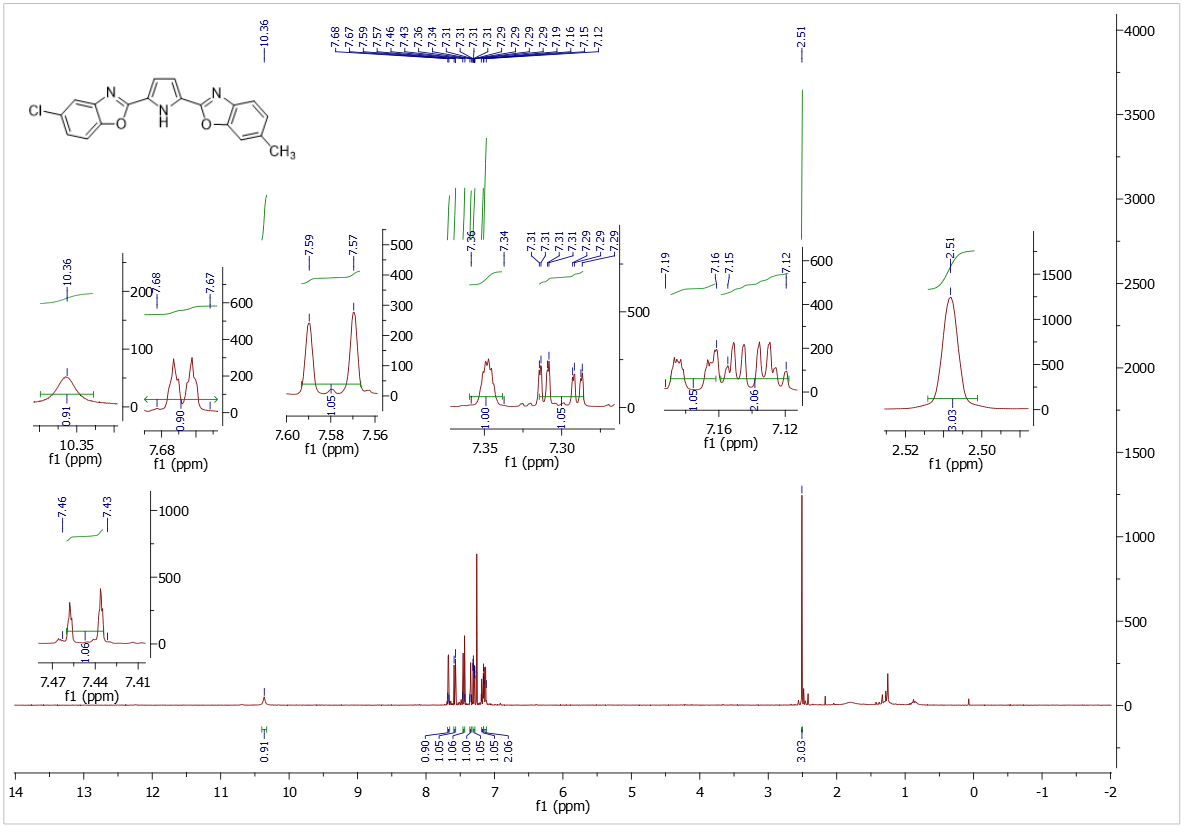


**Figure S37.** ^1^H-NMR Spectrum of compound **B19**


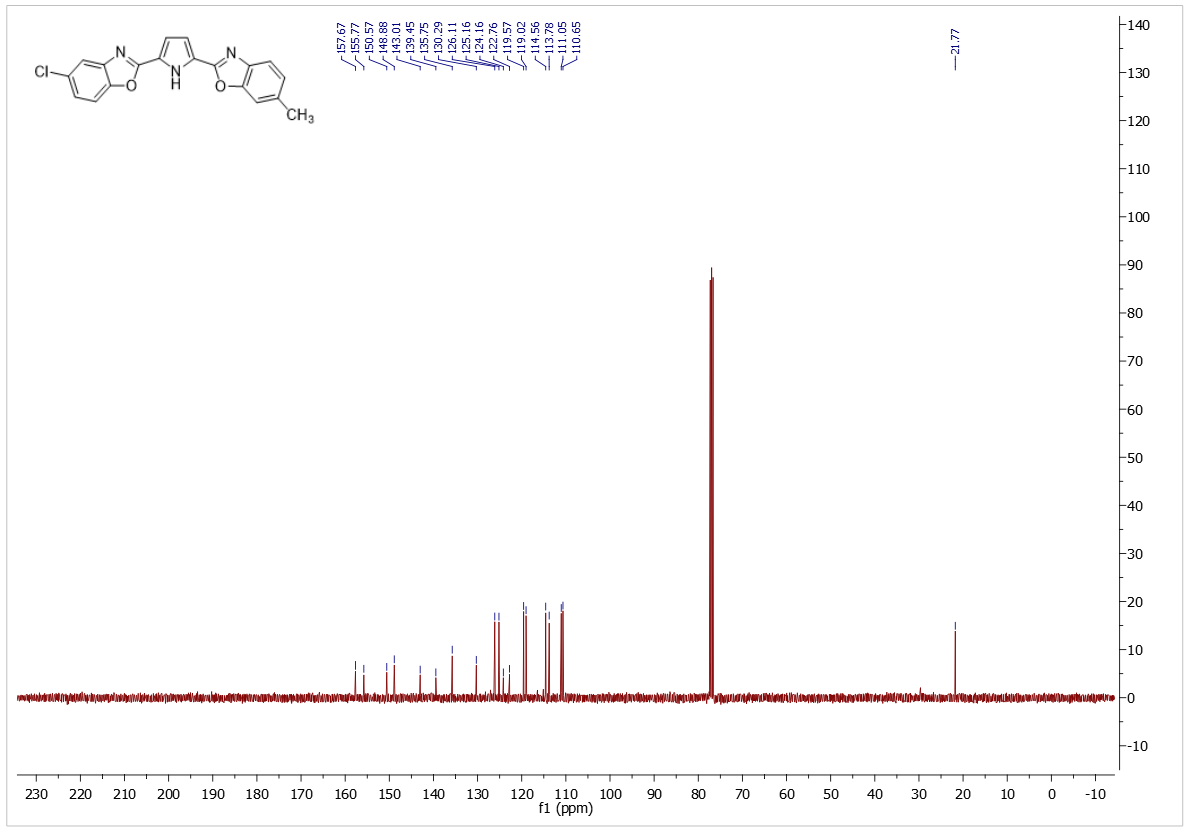


**Figure S38.** ^13^C-NMR Spectrum of compound **B19**


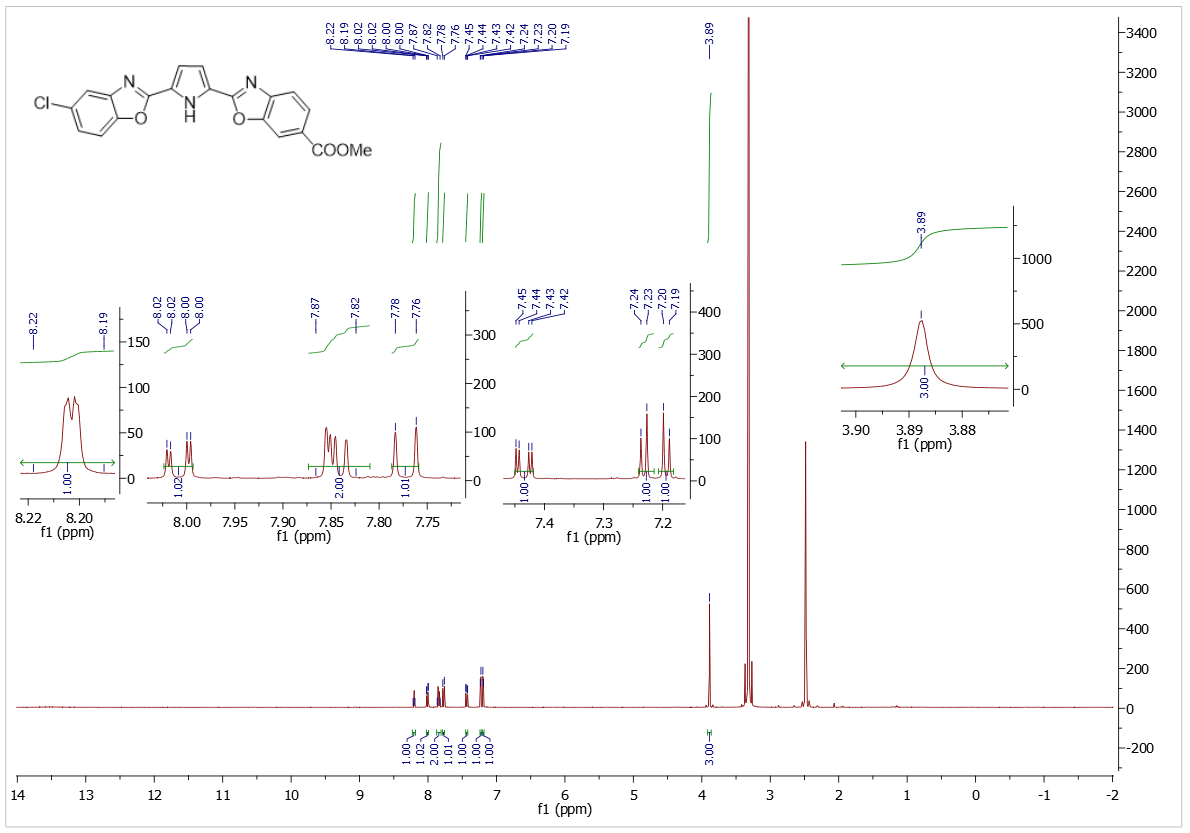


**Figure S39.** ^1^H-NMR Spectrum of compound **B20**


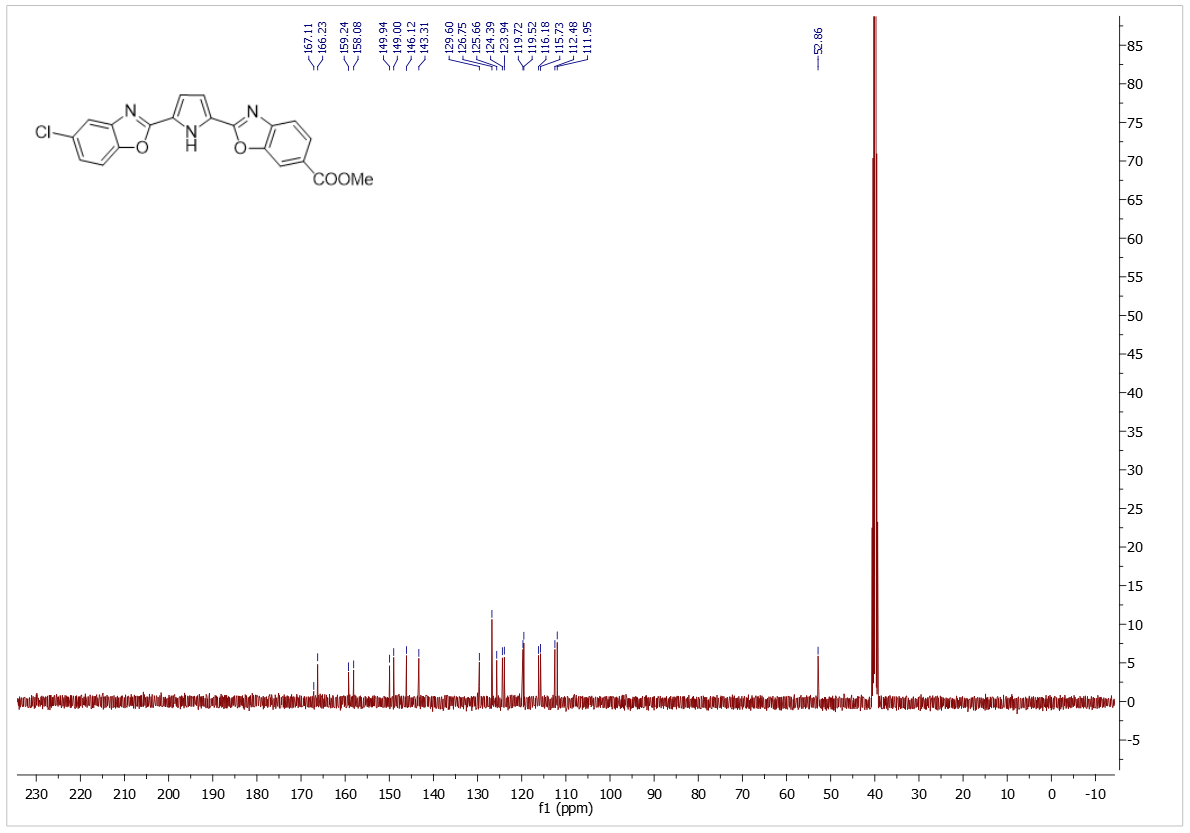


**Figure S40.** ^13^C-NMR Spectrum of compound **B20**

1. **Analysis of apoptosis and necrosis of MCF-7 and fibroblast (HDF) cells (48h)**


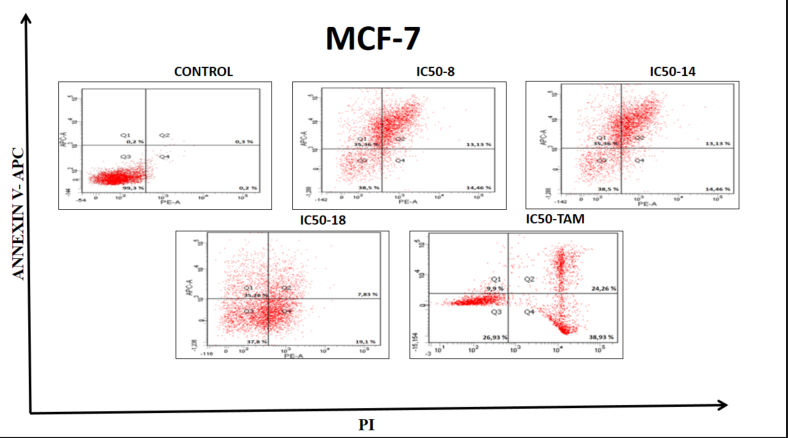


**Figure S41**. Apoptosis and necrosis analysis of MCF-7 cells was evaluated in flow cytometry, after therapy for 48 h. Apoptosis data; live cells (Q3), early apoptosis (Q1), late apoptosis (Q2) and necrosis (Q4) populations were identified by plotting Annexin V staining intensity versus PI staining intensity.


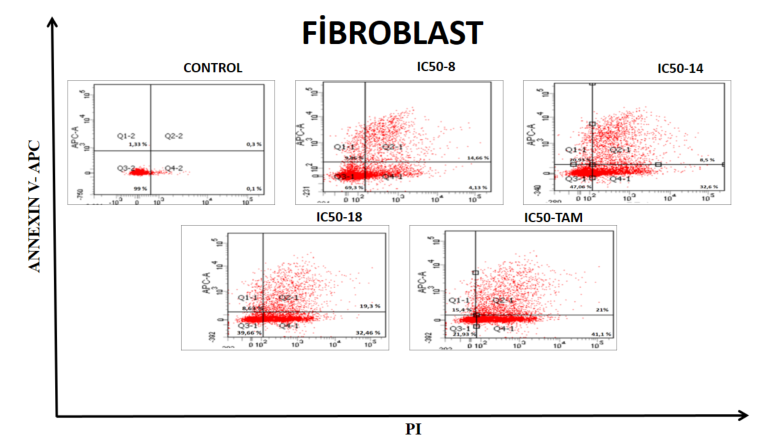


**Figure S42**. Apoptosis and necrosis analysis of Fibroblast cells (HDF) was evaluated in flow cytometry, after therapy for 48 h. Apoptosis data; live cells (Q3), early apoptosis (Q1), late apoptosis (Q2) and necrosis (Q4) populations were identified by plotting Annexin V staining intensity versus PI staining intensity.

1. **Cell-cycle analysis of MCF-7 and fibroblast (HDF) cells (48h)**


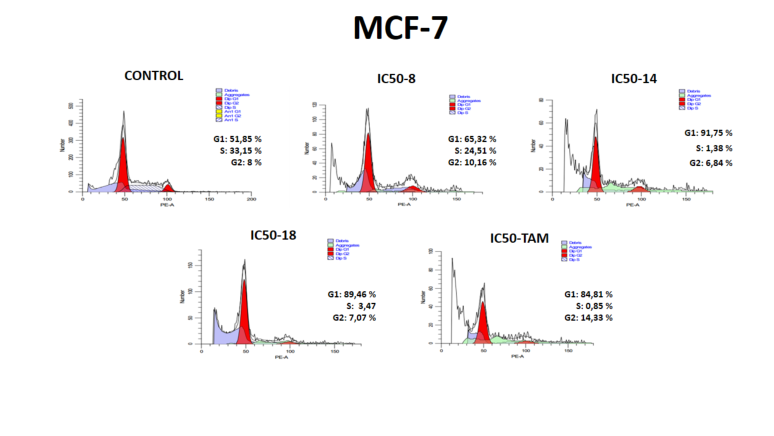


**Figure S43.** Cell-cycle analysis of MCF-7 cells was evaluated in flow cytometry, after therapy for 48 h.


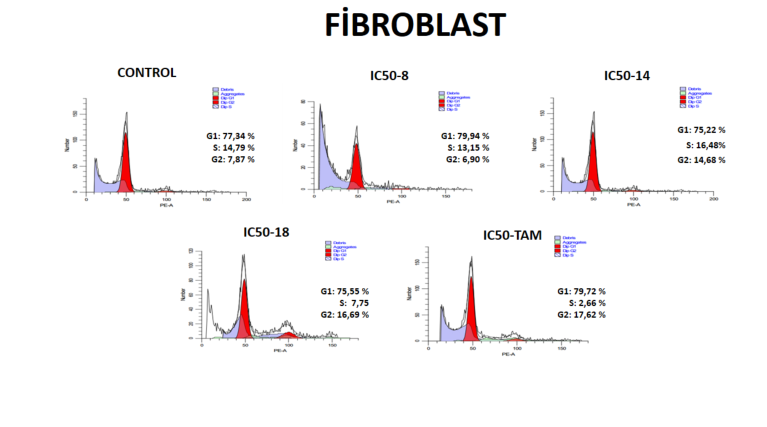


**Figure S44.** Cell-cycle analysis of Fibroblast cells was evaluated in flow cytometry, after therapy for 48 h.

1. A time-dependent graph of cell index values of B8 in MCF-7


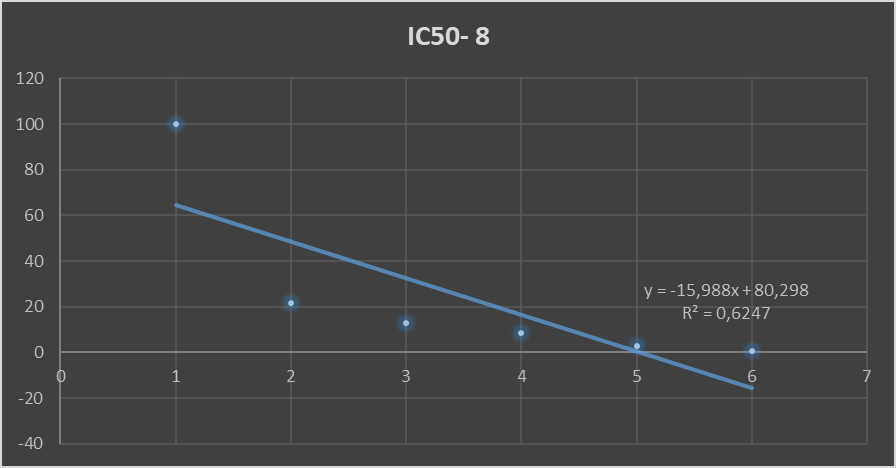


**Figure S45.** A time-dependent graph of cell index values of different concentrations in MCF-7 cells for 48 h was calculated.

1. A time-dependent plot of B8, 14, 18 and Tamoxifen IC_50_ values in HDFa cells at 72 hrs

**
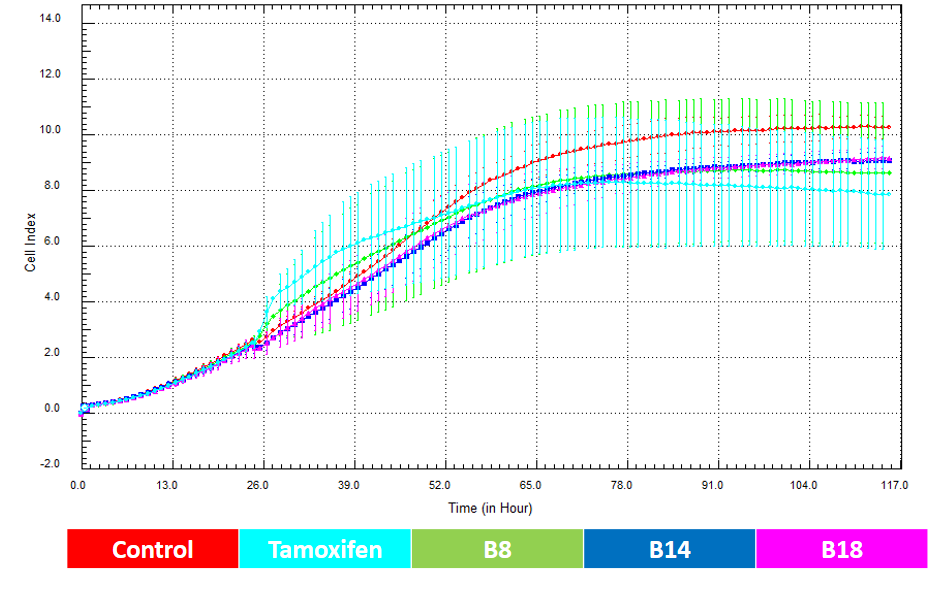
**

**Figure S46.** A time-dependent plot of B8, 14, 18 and Tamoxifen IC_50_ values in HDFa cells at 72 hours calculated in RTCA Software 1.2.1

1. **Table S1.** Forward and Reverse Primer PCR Sequences for Real-time PCR

| **Primers** |  | **Sequence of Nucleotides (nt)** | **Size (nt)** | **PCR Product length** |
| --- | --- | --- | --- | --- |
| Bax (55°C) | Forward | 5’-ATGTTTTCTGACGGCAACTTC-3’ | **21** | 133 bp |
|  | Reverse | 5’-AGTCCAATGTCCAGCCCAT-3’ | **19** |  |
| Bcl-2 (65°C) | Forward | 5’-ATGTGTGTGGAGACCGTCAA-3’ | **20** | 141 bp |
|  | Reverse | 5’-GCCGTACAGTTCCACAAAGG-3’ | **20** |  |
| p53 (60°C) | Forward | 5’-ATGTTTTGCCAACTGGCCAAG-3’ | **21** | 153 bp |
|  | Reverse | 5’-TGAGCAGCGCTCATGGTG-3’ | **18** |  |
| Caspase-9 (60°C) | Forward | 5’-CGACATGATCGAGGATATTCAG-3’ | **20** | 210 bp |
|  | Reverse | 5’-TGCCTCCCTCGAGTCTCA-3’ | **20** |  |

1. **References**
2. Kuzu, B., Sari, O., Erdem, S. S., Algul, O., & Menges, N. (2021). Synthesis of Benzoxazole‐2‐carboxylate Derivatives: Electronic‐and Position‐effect of Functional Groups and Computational Modeling of the Selectivity for Oxazole Ring. ChemistrySelect, 6(10), 2529-2538.
3. Kuzu, E., & Kuzu, B. (2023). Tandem Synthesis of Novel thiazole-substituted pyrrolo [1, 2-d][1, 2, 4] triazin-4 (3 H)-one Derivatives and their Theoretical Pharmacokinetic Profiles. *Chemistry of Heterocyclic Compounds*, *59*(1-2), 80-87.
